# Does the Pachytene Checkpoint, a Feature of Meiosis, Filter Out Mistakes in Double-Strand DNA Break Repair and as a side-Effect Strongly Promote Adaptive Speciation?

**DOI:** 10.1093/iob/obac008

**Published:** 2022-04-08

**Authors:** Victoria E Foe

**Affiliations:** Department of Biology and Friday Harbor Laboratories, University of Washington, Friday Harbor WA 98250, USA

## Abstract

This essay aims to explain two biological puzzles: why eukaryotic transcription units are composed of short segments of coding DNA interspersed with long stretches of non-coding (intron) DNA, and the near ubiquity of sexual reproduction. As is well known, alternative splicing of its coding sequences enables one transcription unit to produce multiple variants of each encoded protein. Additionally, padding transcription units with non-coding DNA (often many thousands of base pairs long) provides a readily evolvable way to set how soon in a cell cycle the various mRNAs will begin being expressed and the total amount of mRNA that each transcription unit can make during a cell cycle. This regulation complements control via the transcriptional promoter and facilitates the creation of complex eukaryotic cell types, tissues, and organisms. However, it also makes eukaryotes exceedingly vulnerable to double-strand DNA breaks, which end-joining break repair pathways can repair incorrectly. Transcription units cover such a large fraction of the genome that any mis-repair producing a reorganized chromosome has a high probability of destroying a gene. During meiosis, the synaptonemal complex aligns homologous chromosome pairs and the pachytene checkpoint detects, selectively arrests, and in many organisms actively destroys gamete-producing cells with chromosomes that cannot adequately synapse; this creates a filter favoring transmission to the next generation of chromosomes that retain the parental organization, while selectively culling those with interrupted transcription units. This same meiotic checkpoint, reacting to accidental chromosomal reorganizations inflicted by error-prone break repair, can, as a side effect, provide a mechanism for the formation of new species in sympatry. It has been a long-standing puzzle how something as seemingly maladaptive as hybrid sterility between such new species can arise. I suggest that this paradox is resolved by understanding the adaptive importance of the pachytene checkpoint, as outlined above.

## Chinese

概要: 本文旨在阐释两个生物学之谜:为什么真核基因是由短片段的编码 DNA穿插着长的非编码 (内含子) DNA 片段构成, 以及为何有性生殖如此广泛地存于真核生物之中。众所周知, 编码序列的可变剪接可以使一个基因产生多种不同蛋白质变体。此外, 用非编码 DNA (通常有数千个碱基对长) 填充转录单元提供了一种易于演化的方式, 它可以设置细胞周期中各种 mRNA 开启表达的时间以及每个基因在一个细胞周期中能够表达的 mRNA的总量。这种调节补充了通过转录启动子的调控, 并促进了复杂的真核细胞类型, 组织, 以及生物体的产生。然而, 它也使真核生物极易受到DNA双链断裂的影响, 因为通过末端连接的断裂修复有可能产生错误。转录单元覆盖基因组的长片段使得任何产生重组染色体的错误修复都很有可能毁坏基因。在减数分裂过程中, 同源染色体通过联会复合体而配对, 由粗线期监查点的检查而选择性地阻断, 而染色体不能有效配对的配子在许多生物体中也会被主动地销毁;这些途径有利于亲本染色体的组织结构能忠实地传递到下一代, 同时有选择地滤除那些转录单元被破坏的染色体。

减数分裂监查点对由于易出错的断裂修复而造成的意外染色体重组作出反应, 而作为其副作用, 可以为在同域物种 (即在同一地理区域并因此经常相互相遇的物种) 中形成新物种提供一种机制。一个存在已久的不解之谜是像杂交不育这样看似不良适应的现象是如何在这些新物种中出现的。我建议通过了解如上所述的减数分裂过程中粗线期监查点在适应性方面的重要作用来解决这个悖论。

## French

Synopsis: Cet essai vise à expliquer deux énigmes biologiques : pourquoi les unités de transcription eucaryotes sont composées de courts segments d'ADN codant entrecoupés de longues portions d'ADN non codant (intron) et la quasi-omniprésence de la reproduction sexuée. Comme nous le savons, l'épissage alternatif des séquences codantes permet à une unité de transcription de produire de multiple variant de chacune des protéines codées. De plus, remplir les unités de transcription avec de l'ADN non codant (souvent plusieurs milliers de paires de bases) fournit un moyen facilement évolutif de définir à quel moment dans un cycle cellulaire les différents ARNm commenceront à être exprimés et quelle quantité totale d'ARNm sera produite par chaque unité de transcription au cours d'un cycle cellulaire. Cette régulation s'ajoute au contrôle par le promoteur transcriptionnel et facilite la création de types cellulaires eucaryotes complexes, de tissus et d'organismes. Cependant, cela rend également les eucaryotes extrêmement vulnérables aux cassures double brin de l'ADN, que les voies de réparation par jonction des extrémités non-homologues peuvent réparer de manière inexacte. Les unités de transcription couvrent une fraction si importante du génome que toute mauvaise réparation produisant un chromosome réorganisé a une forte probabilité de détruire un gène. Au cours de la méiose, le complexe synaptonémal aligne les paires de chromosomes homologues et le point de contrôle du pachytène détecte, arrête sélectivement et dans de nombreux organismes détruit activement les cellules productrices de gamètes possédant des chromosomes qui ne peuvent pas s'apparier correctement. Cela crée un filtre favorisant la transmission à la génération suivante de chromosomes conservant l'organisation parentale, tout en éliminant sélectivement ceux dont les unités de transcription ont été interrompues. Ce même point de contrôle méiotique, réagissant aux réorganisations chromosomiques accidentelles résultantes d'erreurs lors de la réparation des cassures double-brin, peut, comme effet secondaire, fournir un mécanisme d’émergence de nouvelles espèces sympatriques. La question de comprendre comment quelque chose d'aussi apparemment inadapté que la stérilité hybride entre ces nouvelles espèces peut survenir reste un casse-tête de longue date. Je suggère que ce paradoxe soit résolu en comprenant l'importance adaptative du point de contrôle du pachytène, comme indiqué ci-dessus.

## German

Zusammenfassung: Dieser Aufsatz zielt darauf ab, zwei biologische Rätsel zu lösen: warum eukaryotische Transkriptionseinheiten aus kurzen Abschnitten kodierender DNA bestehen, die mit langen Abschnitten nichtkodierender (Intron) DNA durchsetzt sind, und die nahezu allgegenwärtige sexuelle Fortpflanzung. Bekanntlich, ermöglicht alternatives Spleißen seiner kodierenden Sequenzen, dass eine Transkriptionseinheit mehrere Varianten jedes kodierten Proteins produziert. Darüber hinaus bietet das Auffüllen von Transkriptionseinheiten mit nichtkodierender DNA (oft viele tausend Basenpaare lang) eine leicht zu entwickelnde Möglichkeit, um festzulegen, wie schnell in einem Zellzyklus die verschiedenen mRNAs exprimiert werden und wie viel mRNA jede Transkriptionseinheit aufnehmen kann während eines Zellzyklus machen. Diese Regulation ergänzt die Kontrolle über den Transkriptionspromotor und erleichtert die Bildung komplexer eukaryotischer Zelltypen, Gewebe und Organismen. Es macht Eukaryoten jedoch auch äußerst anfällig für DNA-Doppelstrangbrüchen, die durch die Non-homologous end-joining Reparaturwege falsch repariert werden können. Transkriptionseinheiten decken einen so großen Teil des Genoms ab, dass jede Fehlreparatur, die ein reorganisiertes Chromosom erzeugt, mit hoher Wahrscheinlichkeit ein Gen zerstört. Während der Meiose richtet der synaptonemaler Komplex homologe Chromosomenpaare aus, und der Pachytän- Kontrollpunkt erkennt, stoppt selektiv und zerstört in vielen Organismen aktiv Gameten-produzierende Zellen mit Chromosomen, die nicht ausreichend Synapsen bilden können; Dadurch entsteht ein Filter, der die Übertragung auf die nächste Generation von Chromosomen begünstigt, die die elterliche Organisation beibehalten, während diejenigen mit unterbrochenen Transkriptionseinheiten selektiv ausgesondert werden. Dieser gleiche meiotische Kontrollpunkt, der auf zufällige chromosomale Reorganisationen reagiert, die durch fehleranfällige Bruchreparatur verursacht werden, kann als Nebeneffekt einen Mechanismus für die Bildung neuer Arten in Sympatrie bereitstellen. Es war lange Zeit ein Rätsel, wie etwas so scheinbar Unangepasstes wie der Sterilität von Hybriden zwischen solchen neuen Arten entstehen kann. Ich schlage vor, dass dieses Paradoxon gelöst wird, indem man die adaptive Bedeutung des Pachytän-Kontrollpunkt versteht, wie oben beschrieben.

## Spanish

Rsumen: Este ensayo tiene como objetivo explicar dos enigmas biológicos: por qué las unidades de transcripción eucarióticas están compuestas de segmentos cortos de ADN codificante intercalados con largos tramos de ADN no codificante (intrones) y la práctica ubicuidad de la reproducción sexual. Como es bien sabido, el corte y empalme alternativo de sus secuencias codificantes permite que una unidad de transcripción produzca múltiples variantes de cada proteína codificada. Además, el relleno de unidades de transcripción con ADN no codificante (a menudo de muchos miles de pares de bases de largo) proporciona un mecanismo evolutivo sencillo para establecer con cuánta rapidez los diversos ARNm comenzarán a expresarse y la cantidad total de ARNm que cada unidad de transcripción puede generar durante un ciclo celular. Esta regulación complementa al control a través del promotor transcripcional y facilita la creación de tipos celulares, tejidos y organismos eucariotas complejos. Sin embargo, también hace que los eucariotas sean extremadamente vulnerables a las roturas de ADN de doble cadena, que pueden ser reparadas incorrectamente por las vías de reparación de roturas de unión de extremos. Las unidades de transcripción cubren una fracción tan grande del genoma que cualquier reparación incorrecta que produzca un cromosoma reorganizado tiene una alta probabilidad de destruir un gen. Durante la meiosis, el complejo sinaptonémico alinea pares de cromosomas homólogos y el punto de control de paquitena detecta, detiene selectivamente y, en muchos organismos, destruye activamente las células productoras de gametos con cromosomas que no pueden hacer sinapsis de manera adecuada; esto crea un filtro que favorece la transmisión a la siguiente generación de cromosomas que retienen la organización parental, al tiempo que elimina selectivamente aquellos con unidades de transcripción interrumpidas. Este mismo punto de control meiótico, que reacciona a las reorganizaciones cromosómicas accidentales infligidas por la reparación de roturas propensa a errores, puede, como efecto secundario, proporcionar un mecanismo para la formación de nuevas especies en simpatría. Durante mucho tiempo, ha sido un enigma cómo puede surgir algo tan aparentemente inadaptado como la esterilidad híbrida entre estas nuevas especies. Propongo que esta paradoja se resuelva comprendiendo la importancia adaptativa del punto de control de paquitena, como se describió anteriormente.

## Russian

Резюме: Это эссе призвано объяснить две биологические загадки: почему эукариотические единицы транскрипции состоят из коротких сегментов кодирующей ДНК, перемежающихся длинными участками некодирующей (интронной) ДНК, и почти вездесущее наличие полового размножения в жизненных циклах эукариот. Как хорошо известно, альтернативный сплайсинг кодирующих последовательностей позволяет одной единице транскрипции продуцировать несколько вариантов каждого кодируемого белка. Кроме того, наполнение единиц транскрипции некодирующей ДНК (часто длиной в несколько тысяч нуклеотидов) обеспечивает простой способ контроля времени начала экспресcии различных мРНК в клеточном цикле, а также общее количество мРНК, которое каждая единица транскрипции может произвести в течение клеточного цикла. Эта регуляционная способность дополняет контроль экспрессии через промотор транскрипции и облегчает создание сложных типов эукариотических клеток, тканей и организмов. В то же время, это также делает эукариот чрезвычайно уязвимыми к двухцепочечным разрывам ДНК, которые могут репарироваться с ошибками путём негомологичного соединения концов. Единицы транскрипции покрывают такую ​​большую часть генома, что любая ошибочная репарация приводящая к реорганизации хромосомы с высокой вероятностью может привести к разрушению гена. Во время мейоза синаптонемный комплекс выравнивает гомологичные пары хромосом, а контрольная точка пахитены обнаруживает, избирательно арестовывает и, у многих организмов, активно разрушает клетки продуцирующие гаметы с хромосомами, которые не могут адекватно синапсировать; это создает фильтр, благоприятствующий передаче следующему поколению хромосом, которые сохраняют родительскую организацию, и выборочно отбраковывающий хромосомы с прерванными единицами транскрипции. Та же самая мейотическая контрольная точка реагирующая на случайные хромосомные реорганизации вызванные склонной к ошибкам репарацией разрывов, может, как побочный эффект, обеспечить механизм образования новых видов в симпатрии. Давно было загадкой, как может возникнуть что-то настолько неадаптивное, как бесплодие гибридов между такими новыми видами. Я полагаю, что этот парадокс разрешается путем понимания адаптивной важности контрольной точки пахитены, как указано выше.

## Portuguese

Resumo: Este ensaio visa explicar dois enigmas biológicos: o porquê das unidades de transcrição eucarióticas serem compostas por segmentos curtos de DNA codificante intercalados por longos trechos de DNA não-codificante (íntron), e a quase universalidade da reprodução sexual. Como é bem conhecido, o *splicing* alternativo de sequências codificantes permite que uma unidade de transcrição produza múltiplas variantes de cada proteína codificada. Além disso, o preenchimento de unidades de transcrição com DNA não-codificante (geralmente muitos milhares de pares de bases) fornece uma maneira pronta para evoluir e determinar o quão cedo no ciclo celular os diversos mRNAs começarão a ser expressos e a quantidade total de mRNA que cada unidade de transcrição irá produzir durante um ciclo celular. Esta regulação complementa o controle através do promotor transcricional e facilita a geração de tipos complexos de células eucarióticas, tecidos e organismos. No entanto, também torna os eucariotos extremamente vulneráveis a quebras de DNA de fita dupla, dado que que os mecanismos de reparo da quebra da fita dupla podem reparar incorretamente. As unidades de transcrição cobrem uma fração tão grande do genoma que qualquer reparo incorreto que produza um cromossomo reorganizado tem uma alta probabilidade de quebrar um gene. Durante a meiose, o complexo sinaptonêmico alinha pares de cromossomos homólogos e o ponto de verificação do paquíteno detecta, interrompe seletivamente e, em muitos organismos, destrói ativamente células produtoras de gametas com cromossomos que não podem fazer sinapse adequadamente; isso cria um filtro que favorece a transmissão de cromossomos que retêm a organização parental para a próxima geração, enquanto seleciona seletivamente aqueles com unidades de transcrição interrompidas. Esse ponto de verificação meiótico, que responde a reorganizações cromossômicas acidentais infligidas por reparos de quebras propensos a erros, pode, como efeito colateral, também ser um mecanismo de formação de novas espécies em simpatria. O enigma de como algo aparentemente com tão baixo valor adaptativo quanto a esterilidade híbrida entre essas novas espécies pode surgir permanece há muito tempo. Eu proponho que esse paradoxo seja resolvido pela compreensão da importância adaptativa do ponto de verificação do paquíteno, conforme descrito acima.

## Norwegian

Abstrakt: Dette essayet tar sikte på å forklare to biologiske gåter: hvorfor eukaryote transkripsjonsenheter er sammensatt av korte segmenter av kodende DNA ispedd lange strekninger av ikke-kodende (intron) DNA og hvofor seksuell reproduksjon er neste allestedsnærværende. Det er velkjent at alternativ spleising av dens kodende sekvenser muliggjør én transkripsjonsenhet til å produsere flere varianter av hvert kodet protein. I tillegg forstørrende transkripsjonsenheter med ikke-kodende DNA (ofte mange tusenvis av basepar) en lett utvikbar måte å utpeke hvor raskt i en cellesyklus de ulike mRNAene vil begynne å bli uttrykt og den totale mengden mRNA som hver transkripsjonsenhet kan lage i løpet av en cellesyklus. Denne reguleringen samarbeider kontroll via transkripsjonspromotoren og letter dannelsen av komplekse eukaryote celletyper, vev og organismer. Imidlertid gjør det også eukaryoter ekstremt sårbare for dobbelttråds-DNA-brudd, som endesammenføyningsreparasjonsveier kan reparere feil. Transkripsjonsenheter dekker en så stor del av genomet at enhver feilreparasjon som produserer et reorganisert kromosom har stor sannsynlighet for å ødelegge et gen. Under meiose justerer det synaptonemale komplekset homologe kromosompar og pachyten-sjekkpunktet oppdager, slutter selektivt og i mange organismer ødelegger aktivt gametproduserende celler med kromosomer som ikke kan synapse tilstrekkelig; dette skaper et filter som favoriserer overføring til neste generasjon av kromosomer som beholder foreldreorganisasjonen, samtidig som de selektivt avliver de med avbrutt transkripsjonsenheter. Dette samme meiotiske sjekkpunktet, som reagerer på tilfeldige kromosomale omorganiseringer påført av feilutsatt bruddreparasjon som en bieffekt kan gi en mekanisme for sympatrisk artsdannelse. Det har lenge vært et puslespill hvordan noe så tilsynelatende mistilpasset som hybridsterilitet mellom slike nye arter kan oppstå. Jeg foreslår at dette paradokset løses ved å forstå at den pachyten-sjekkpunktet har avgjørende implikasjoner for organismers tilpassingsevne, som skissert ovenfor.

**Abbreviations**
TU: transcription unit

## Introduction and essay roadmap

The main thesis in this essay is that sexual reproduction in eukaryotes combines two critical functions that increase the probability that organisms can transmit well-adapted and complete genomes from one generation to the next. The first function, well known and extensively studied, is produced by the genetic recombination events that reshuffle genes between paired homologous chromosomes during meiosis. Rare unavoidable errors in maintaining DNA sequences will occasionally improve a gene's function, though more often base pair changes degrade gene performance. During each meiosis, recombination reassembles gene variants in new combinations, increasing the chance for at least some gametes to generate healthy and well-adapted offspring. I propose that meiosis provides a second essential function through a gamete screening process known as the pachytene checkpoint. I will argue that this checkpoint acts as a filter, selectively arresting or killing those gamete-producing cells that are the most likely to have lost entire genes due to an earlier mis-repair of double-strand DNA breaks; specifically, it is chromosomal rearrangement (inversions and translocations), which the checkpoint is selecting against. However, if inversions have captured sufficiently adaptive alleles, I explain how the pachytene checkpoint can instead drive new species formation, even within a freely interbreeding population. These arguments are laid out in the *second* half of this essay.

The *first* half of this essay describes the differences between prokaryotic and eukaryotic genomes that have made the pachytene checkpoint necessary. Roughly 2.5 billion years ago, self-splicing introns gained a foothold in the genomes of earlier life forms in enormous abundance. Although those stretches of non-coding DNA are now integral to all eukaryotic genomes and contribute to transcriptional regulation, profound cellular adaptations were required before organisms could survive and ultimately make use of them. One of those accommodations was contending with the frequent double-strand DNA breaks that pose a dire threat to organisms which, due to the inclusion of introns, often require that tens of thousands of DNA base pairs be completely transcribed to express some of their mRNAs. As necessary background, I briefly review the several eukaryotic DNA break repair pathways and the synaptonemal complex, which is the eukaryotic structure that creates the pachytene checkpoint. I argue that this meiotic checkpoint makes large eukaryotic genomes heritable by reducing the probability that those genomes that have lost genes due to low-fidelity DNA break-repair will be passed to the next generation.

Aiming to engage a cross-disciplinary audience, in both halves of this essay I shall review aspects of biological knowledge that are certain to be overly familiar to one segment of readers, but which others may be unaware of. For this and the manuscript's resulting length, I ask forbearance. My electron micrographs illustrate various key points.

## Materials and methods

The chromatin of *Drosophila* embryos was prepared for TEM viewing as described by McKnight and Miller, with attention to the details noted below. The chromatin dispersal protocol discovered by Oscar Miller and used throughout the 1970s and 1980s correctly prescribes a dispersal medium of freshly prepared distilled water adjusted to pH 8.5–9.0 with the minimum amount of borate buffer (Miller and Beatty 1969). Whether or not this was understood at the time, it turns out the reason it must be fresh is that CO_2_ readily dissolves in open containers of water and reacts to create H_2_ CO_3_, which as it dissociates lowers the solution pH. For that reason, micro-filtered water with its large amount of dissolved CO_2_ does not substitute for freshly distilled water. However, collection of hot, freshly distilled water, adjusted to pH 8.5–9.0 and stored in a capped bottle with no head of air retains indefinitely this pH and its ability to unfold chromatin.

*Drosophila* were reared using standard methods, 2–3 h egg collections were made from a single bottle of flies. Eggs were dechorionated for 1.5 min in Chlorox diluted 1:1 with fly wash (8 gm/L NaCl; 0.5 mL/L Triton X 100), collected on a screen, rinsed, and transferred into a petri dish of fly wash for sorting under a dissection scope. Five–ten embryos at nuclear cycle nine (pole bud formation; see Foe and Alberts 1983) were selected and transferred by pipette to a new petri dish of fly wash and allowed to develop in a 25°C incubator for 45–50 mins (to mid-interphase of nuclear cycle 13). Embryos were transferred to an eight-well slide, one embryo per well and monitored at room temperature (21°C) using a compound microscope to observe nuclear envelope breakdown at nuclear cycle 13 mitosis (see Foe and Alberts 1983), then timed from the first reappearance of discrete round nuclei (start of cycle 14 interphase). For lysis, embryos were transferred by pipette onto a sheet of Parafilm under a dissecting microscope, rinsed with distilled water and macerated with forceps in the pH 8.5–9 dispersal medium (1 embryo per 100µl). Micrograph figure legends give *Drosophila* embryo age at lysis. The anaphase chromosome micrograph is from a syncytial blastoderm-stage *Oncopeltus fasciatus* (milkweed bug) embryo (about 19 h post-oviposition; 21°C), prepared similarly to the *Drosophila* interphase chromatin, with the small modifications described by Foe et al. (1976).

**Fig. 1:**
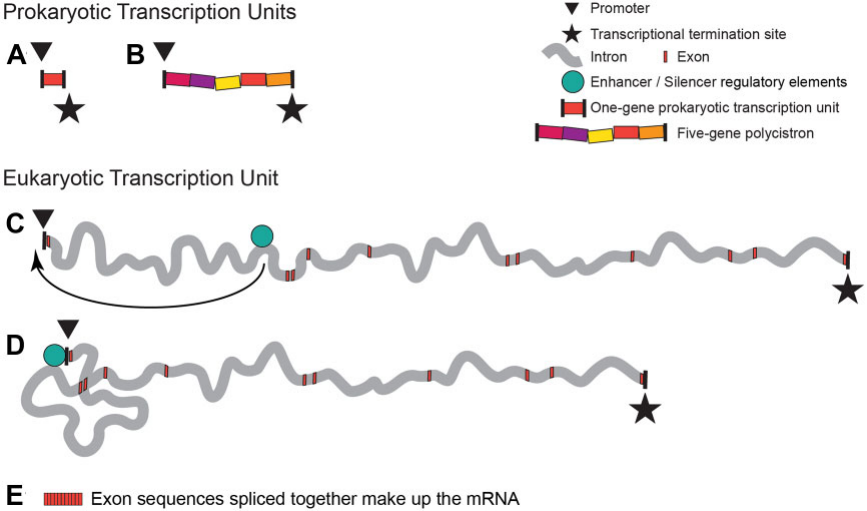
Diagram illustrating the different organization of transcription units in prokaryotes and eukaryotes. A triangle and a star indicate the transcriptional promoter and termination site which demarcate individual TUs. A–E are drawn to the same scale. A depicts the length of DNA equivalent to an average-length, 1000 bp bacterial TU; B the 6500 bp trp operon—a large polycistron that E. coli uses to encode the five enzymes with which this bacterium synthesizes the amino acid tryptophan; and C depicts the mean length of TUs in Homo sapiens—66,646 bp—composed of 11 exons (red) and 10 longer but varied-length (grey) introns (Piovesan et al. 2019). Exons are depicted as if identical in length, whereas in actuality they tend to be short but are not uniform. In C, an enhancer/silencer site is shown in the first intron where such sites are often located, with the blue circle representing bound regulatory elements. D depicts these elements binding immediately upstream of the promoter to regulate Pol II loading. E indicates that eukaryotic mRNA is comprised exons spliced together. Due to untranslated 5’ and 3’ sequences, eukaryotic mRNA is about twice as long as would be needed just to encode a protein. The untranslated 5’ region of the mRNA is encoded in the first exon(s), the middle exons encode amino acids, and the last exon encodes the nontranslated 3’ sequence of the mRNA. The untranslated sequence at each end of the mRNA molecule regulates where within a cell the mRNA will localize, its stability, how many times it is to be translated by a ribosome etc.; this contributes to a usually greater length for eukaryotic vs prokaryotic mRNAs (e.g., A vs. E).

Support films were pure carbon on 200 mesh copper grids (Ted Pella Inc cat # 01840). Prior to use, grids were cleaned by glow discharge for 6 mins in a Denton DV-502 vacuum evaporator. The rest of the chromatin preparation protocol was as described previously (McKnight and Miller 1979). PTA-stained grids were lightly rotary shadowed with platinum/palladium (Ted Pella Inc cat # 24-2) at a low angle—between 6.5° and 7°, in a Denton DV-502 vacuum evaporator. Imaging was with a Phillips CM10 transmission electron microscope at 21,000 or 28,500 X. Grids were scanned using an AMT Advantage 1-megapixel side-mounted camera. Final images were captured by a bottom-mounted SIA L5C 8-megapixel camera. To show large fields of view, images were montaged together using Hugin free software (http://hugin.sourceforge.net/download/).

## Results and discussion

### Regulated DNA transcription is common to all life forms on Earth

During the past 75 years we have gradually learned how, similarly in all three domains of life—the Eubacteria, the Archaea, and the Eukarya—genes encode proteins and the amino acid composition of proteins endows cells with their legion of properties. We have learned that many eukaryotic genes have counterparts in bacteria and archaea, and that many species differ from one another less by the specific proteins their genomes encode than by when, where, and how much of each protein they express. Crucially, it is the precisely timed transcription of different genes in prescribed amounts that guides cells to adopt their different forms and functions. Even single-celled eukaryotes express just a subset of their genomes at any given moment, for example, switching genes on or off depending on available food sources. Every multicellular eukaryote begins life as a single-cell zygote and develops by round after round of cell division during which different genes turn on in different cells in set temporal sequence and amount to build each part of the organism. A large part of the difference between hummingbirds and whales, both vertebrates, is due to differences in the timings and amounts by which highly similar genes are deployed in individual cells. Plainly, the regulation of gene expression is decisive for producing Earth's myriad different living organisms.

So, how *is* gene expression regulated? Historically, a gene was defined as the length of DNA, comprising a specific sequence of nucleotides, that encodes one kind of protein (Beadle and Tatum 1941). Later it was discovered that genes, as thus defined, exist within transcription units (TUs), and that in eukaryotes, these TUs can be vastly longer than their protein-encoding component (Gilbert 1978; Neugebauer and Roth 1997). A transcription unit (TU) is defined as that stretch of DNA bounded by a DNA sequence specifying transcriptional initiation and a second DNA sequence specifying transcriptional termination. This essay explores the very far-reaching consequences of the peculiar organization and the frequently enormous lengths of the many thousands of TUs that encode proteins in eukaryotes.

The most fundamental level of transcriptional regulation in Eubacteria, Archaea, and Eukarya is similar. It is directed by molecules (proteins and RNAs) that—by binding to a promoter DNA sequence, or to molecules already bound to such a sequence—determine whether and how effectively RNA polymerases attach to DNA and initiate transcription (Harley and Reynolds 1987; Kanhere and Bansal 2005; Lenhard et al. 2012; Weingarten-Gabbay and Segal 2014). Fig. 1 shows the basic layout of bacterial TUs (1A and 1B) and eukaryotic TUs (1C and 1D), with the promoter located immediately upstream of the transcriptional start site. Cells may additionally employ secondary regulatory sites, enhancers, and silencers. These sites, often situated far from the promoter they regulate, are loci where large numbers of macromolecules (proteins and RNAs) can attach, interact, and integrate complex regulatory information (Bagga et al. 1998). DNA folding allows such regulator-encrusted enhancers and/or silencers to contact and modulate the effects of molecules already bound to the promoter. 1C makes the point that a TU enhancer is located on the same DNA molecule as the TU it regulates, and 1D illustrates this enhancer contacting a promoter. Although these outboard sites supply critical informational input, what is being controlled is still productive RNA polymerase binding to the transcriptional initiation site, thereby determining each TU's rate of transcriptional initiation.

Once firmly bound, each RNA polymerase pries open the DNA double helix and moves along the DNA, synthesizing a complementary RNA copy of one strand of the double helix (Cosma, 2002; Hahn 2004). It transcribes the DNA processively (i.e., without releasing the DNA substrate) until reaching a termination sequence. Downstream of this transcriptional termination site, RNA polymerase lets go of the DNA and releases the RNA transcript that it has made (Kuehner et al. 2011). Different modifications may be added to the two ends of each transcript to convert it into a functional messenger RNA molecule (mRNA), and in eukaryotes this mRNA must be exported out of the nucleus into the cytoplasm. Each mRNA's unique linear sequence of nucleotides then recruits, via transfer-RNA adaptors, a unique linear sequence of amino acids, which the ribosome links together to produce the specific protein that is encoded by one TU. This is the “Central Dogma” of molecular biology: the genetic information hard-wired into DNA is transcribed to produce individual transportable cassettes—messenger RNAs—each of which directs the synthesis of a specific type of protein molecule (Crick 1958).

### Eukaryotic and prokaryotic transcription units are organized very differently

In prokaryotes (Eubacteria and Archaea), a TU that encodes one protein is not much larger than the DNA needed to specify that protein's amino acids (Fig. 1A). Sometimes several functionally-related proteins are encoded one right after the other (Fig. 1B), with the several genes comprising these “polycistronic” TUs being transcribed from a single promoter to create one mRNA molecule (Mao et al. 2015). Yet even these multi-gene prokaryotic TUs contain little DNA beyond what codes for proteins. The situation in the Eukarya is different. First of all, eukaryotic mRNAs are longer than prokaryotic ones. They include *untranslated* sequences at the mRNA 3’ and 5’ ends that regulate translation. Additionally, the median length of the *translated* portion of eukaryotic mRNAs is about a third longer than the mRNAs of orthologous prokaryotic proteins, due to encoding interaction domains that help eukaryotic proteins assemble into multi-protein complexes (Brocchieri, 2005). But, more significantly, eukaryotic TUs can be enormously long due to an inclusion of DNA whose sequence will *not* be included in the mRNAs, *even though it is transcribed*. For example, in *Homo sapiens* the mean length of the protein-encoding sequence is 1,652 bp, whereas the mean length of human TUs is nearly 67,000 bp, most of which results from the transcription of non-coding DNA sequences (Piovesan et al. 2019). Fig. 1 illustrates the dramatically different lengths of a 67,000 bp long TU (D and E), as compared to TUs of the bacterium, *E. coli* (A and B).

Not all eukaryotes have similarly long TUs (Deutsch and Long 1999). For example, although the budding and fission yeasts, *Saccharomyces cerevisiae* and *S. pombe*, encode proteins of the same average size as human proteins (Brocchieri 2005), their longest TU is only about twice as long as its protein-encoding sequence (Kupfer et al. 2004). However, short TUs, as we will see, are almost certainly a secondary adaptation and not representative of the TU organization of ancestral proto-eukaryotes.

**Fig. 2:**
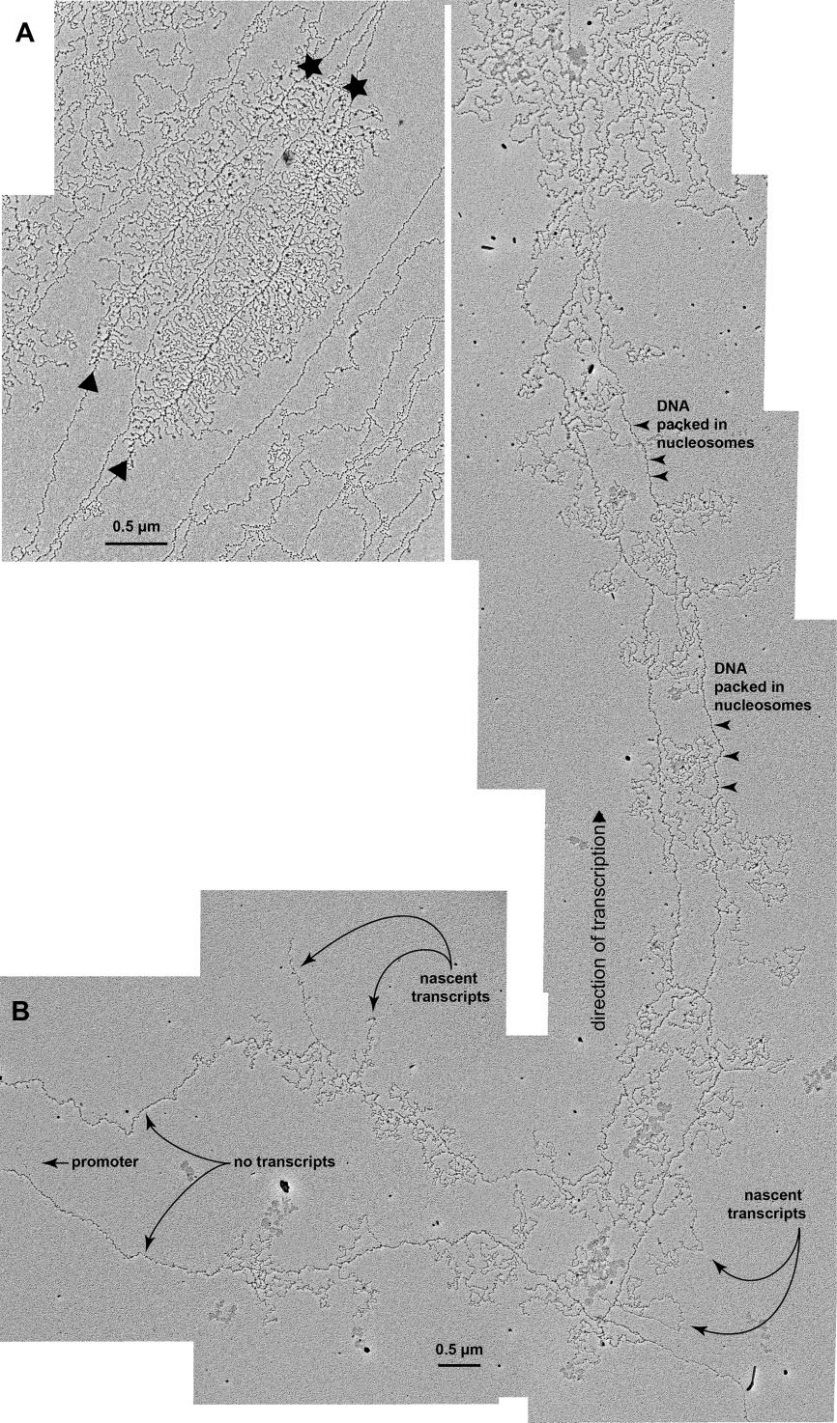
Drosophila transcription units can be very much longer than needed just to encode proteins. Miller spreads of chromatin from Drosophila embryos 19 mins (A) and 120 mins (B) after cycle 14 interphase begins (21°C). Transcription is from left to right. Replicated TUs appear as side-by-side paired arrays of transcripts. In A the sites of transcriptional initiation and termination are marked with a triangle and a star, respectively; this densely-transcribed TU corresponds to six microns of DNA (17,500 bp); at about 55 and 85% of TU length spliceosomes are visible as small black specks on the nascent transcripts and splicing is manifested by transcript length discontinuities in the Christmas tree-shaped array of nascent transcripts. In B, Pol II-transcribed chromatin is visibly packaged into nucleosomes (arrowheads). In non-transcribed beads-on-a-string nucleosome-containing chromatin the mean DNA/chromatin packing ratio is 2.3 vs.1.6 in densely transcribed chromatin (A); this packing ratio is 1.9 in sparsely transcribed chromatin (B)—see Foe et al. 1976. Thus, in B the 25.5 microns of chromatin associated with nascent transcripts corresponds to about 75,000 bp. However, in this array the entire TU is even longer than 75,000 bp because transcriptional initiation occurred an unknown distance upstream of the first transcript in this array. In Homo sapiens the median and mean lengths of protein coding sequence per TU are 1290 bp and 1550 bp respectively (Piovesan et al. 2019). These values are likely to be similar in Drosophila. Micron bars are 0.5 um—equivalent to 1500 bp of B-form DNA (enough to encode a 500 amino acid protein).

The first images of just how much longer eukaryotic TUs can be than prokaryotic TUs came from electron micrographs of chromatin dispersed using the Oscar Miller protocol (Foe et al. 1976; Laird and Chooi 1976; McKnight and Miller 1979). Fig. 2 presents examples of this kind of image, capturing TUs of the fruit fly, *Drosophila melanogaster*, being actively-transcribed. The TUs shown (2A and 2B), prepared from nuclear cycle 14 *Drosophila* embryos, occur as side-by-side pairs because DNA synthesis has already occurred and sister chromatids remain in proximity.

TUs range greatly in size, but even in the fruit fly's relatively small genome, the length of individual TUs can be striking. 2A shows a very densely-transcribed, ordinary-length *Drosophila* TU. At 17,000 bp it is 10 times longer than is required to encode an average-size human or *Drosophila* protein (indicated by the 0.5 micron scale bar).

In *Drosophila's* 14th embryonic cell cycle, the longest transcript arrays are only seen in late interphase, together with shorter TUs. This means that some TUs must be sufficiently long that RNA polymerase II (Pol II) does not reach transcriptional termination sites until late in that cell cycle, if then. A TU from this developmental period is shown in 2B; here the nascent transcripts are spaced far enough apart that it is apparent the TU chromatin is packaged into nucleosomes (arrowheads in 2B). Were its nucleosomal chromatin unfolded into B-form DNA for direct comparison with the length of DNA required to encode an average-size protein (indicated by the 0.5 micron scale bar), the TU's DNA length would be almost double its chromatin length (see DNA/chromatin packing ratio details in legend). Moreover, the transcriptional initiation site lies outside of the field of view, so the entire length of this TU—between transcriptional initiation and termination—spans considerably more than the 75,000 bp here visibly associated with nascent transcripts.

**Fig. 3:**
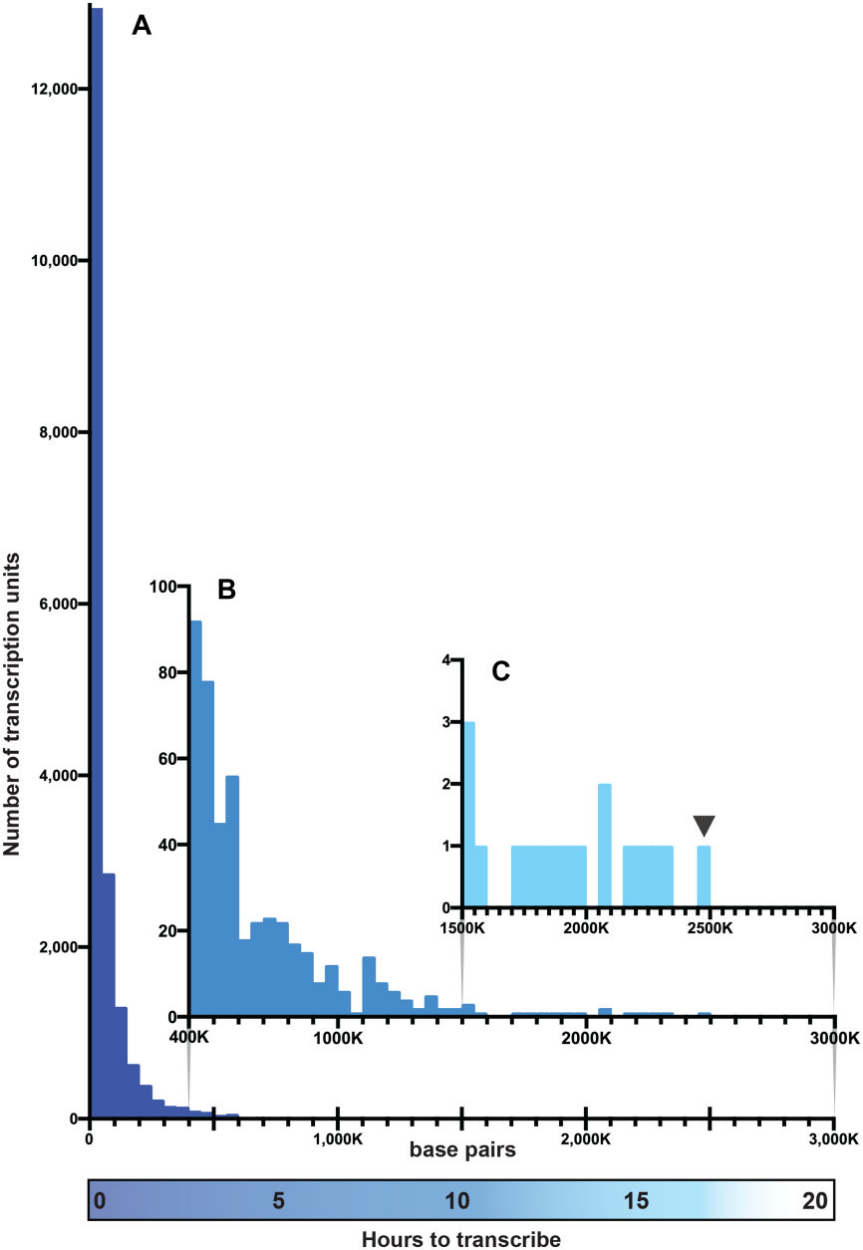
The relative abundance of the different size classes of human TUs. The lengths in base pairs of protein-encoding TUs (X-axis) are plotted against the number of TUs in each size class (Y-axis). TUs are grouped by size into 50,000 bp bins. For the Y-axis three scales are used to display the large range in abundance: thousands of TUs in A; tens of TUs in B; single TUs in C. The X-axis also includes an estimate of the minimum time required to transcribe the various length TUs, assuming Pol II transcribes 2.5 Kb per min (this makes no allowance for transcriptional obstacles and is virtually certain to be a significant underestimate for long TUs). The smallest human gene, KRTAP6-2 (189 bp, chromosome 21) could be transcribed in 5 seconds; the largest RBFOX1 (2,473,592 bp, chromosome 16; indicated with an arrowhead) would take at least 17h. The mean TU length is 66,646 bp and the median length is 26,018 bp. The data of validated genes binned by size were assembled and provided courtesy of Allison Piovesan (Piovesan et al. 2019).

Fig. 3 shows the length distribution of the TUs in the human genome, grouped into bins of increasing size, each bin including lengths up to 50,000 bp larger than the previous bin. A TU of the size shown in 2A would be in the most numerous first bin (0–50,000 bp), and that in 2B in the second bin (51,000–100,000 bp). 17 percent of human TUs are longer than 100,000 bp, that is, longer than the *Drosophila* TU shown in 2B. In fact, 67 of human TUs are between 10 and 20 times that length (Piovesan et al. 2019). The longest human TU is 2.5 million bp (Piovesan et al. 2019) and the longest *Drosophila* TU is 4.3 million bp (Fingerhut et al. 2019).

This essay focuses on some of the consequences of the transcription by Pol II of such enormous lengths of eukaryotic DNA. Both the transcribed and non-transcribed DNA that lacks protein-encoding information has been called “junk DNA” (Ohno 1972; Doolittle and Brunet 2017). I too use this name for emphasis, although I hope to convince the reader that much of the *transcribed junk* is critical to eukaryotic gene regulation. I will argue as well that the existence of so much transcribed junk has had profound consequences for the evolution of the eukaryotic cell from its prokaryotic predecessor, and for the rise of complex multicellular organisms.

### Eukaryotic TU's are not only longer than bacterial genes, but also have a most peculiar organization

The protein-encoding component of the TU, its so-called “**exons**” that will be **ex**pressed by translation into protein, exist as short *discontinuous* segments. **In**serted between consecutive exons are 10 to 100 times longer stretches of “junk” DNA, termed “**introns**” (Gilbert 1978). In the human genome the mean number of introns per protein-encoding TU is 10, and the mean number of exons is 11, as diagrammed in Fig. 1C (Piovesan et al. 2019). As Pol II traverses long TU's, RNA/protein complexes called spliceosomes assemble on the nascent transcripts, snip out, and release the non-coding RNA transcribed from the stretches of junk DNA, and ligate together the small stretches of amino acid-encoding RNA sequence transcribed from the exons (Sharp 1994).

**Fig. 4:**
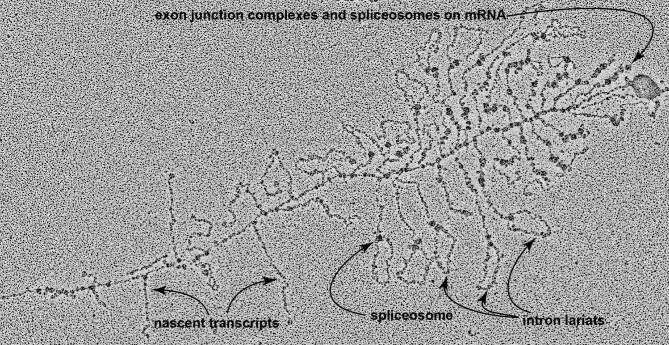
Nascent transcripts undergo splicing during transcription. A four micron-long non-ribosomal TU from a Drosophila embryo 30 min into interphase 14. Spliceosomes assemble at the intron-exon junctions and lariats are evident where introns are being clipped out of the nascent transcripts. Direction of transcription is from left to right; transcripts get longer the further they are from the transcriptional initiation site until spliceosomes begin shortening transcripts by removing introns. In many transcripts the base of the lariat has two bound particles—probably one spliceosome and one exon junction complex. At the distal (right hand) end of the TU, the mRNA, with these enormous multi-molecular complexes still attached, has been shortened by intron removal to 0.5 microns.

All eukaryotic introns have three sequences that spliceosomes recognize: the 5’ splice site, the 3’ splice site, and a nucleotide sequence near the intron's 3' end. During the first step in intron removal, this third sequence is covalently linked to the intron's 5’ splice site, transiently making a “lariat” of the junk RNA (Padgett et al. 1986; Guthrie and Patterson 1988). This lariat intermediate is subsequently cleaved at the 3' splice site as part of a reaction that joins the two adjacent exon sequences into a continuous stretch of coding sequence; this also removes the lariat of junk RNA, which is broken down and its nucleotides recycled. Fig. 4 presents an electron micrograph of an actively transcribed *Drosophila* TU, showing spliceosomes, intron lariats, and newly spliced transcripts. Only after an RNA polymerase with attached nascent RNA has transcribed the most promoter-distal of its exons, and all of the intervening introns have been removed, is the final mRNA formed, composed of the sum of the TU's exons (as indicated in Fig. 1E).

In Eubacteria and Archaea, genes are typically arrayed serially around a single circular chromosome. DNA replication initiates from a one fixed site on the chromosome and the transcription of each TU (or polycistron) is controlled individually (O'Donnell et al. 2013). Prokaryotic cells lack a nuclear envelope, so replication, transcription, and translation take place concurrently in the same cellular compartment.

**Fig. 5:**
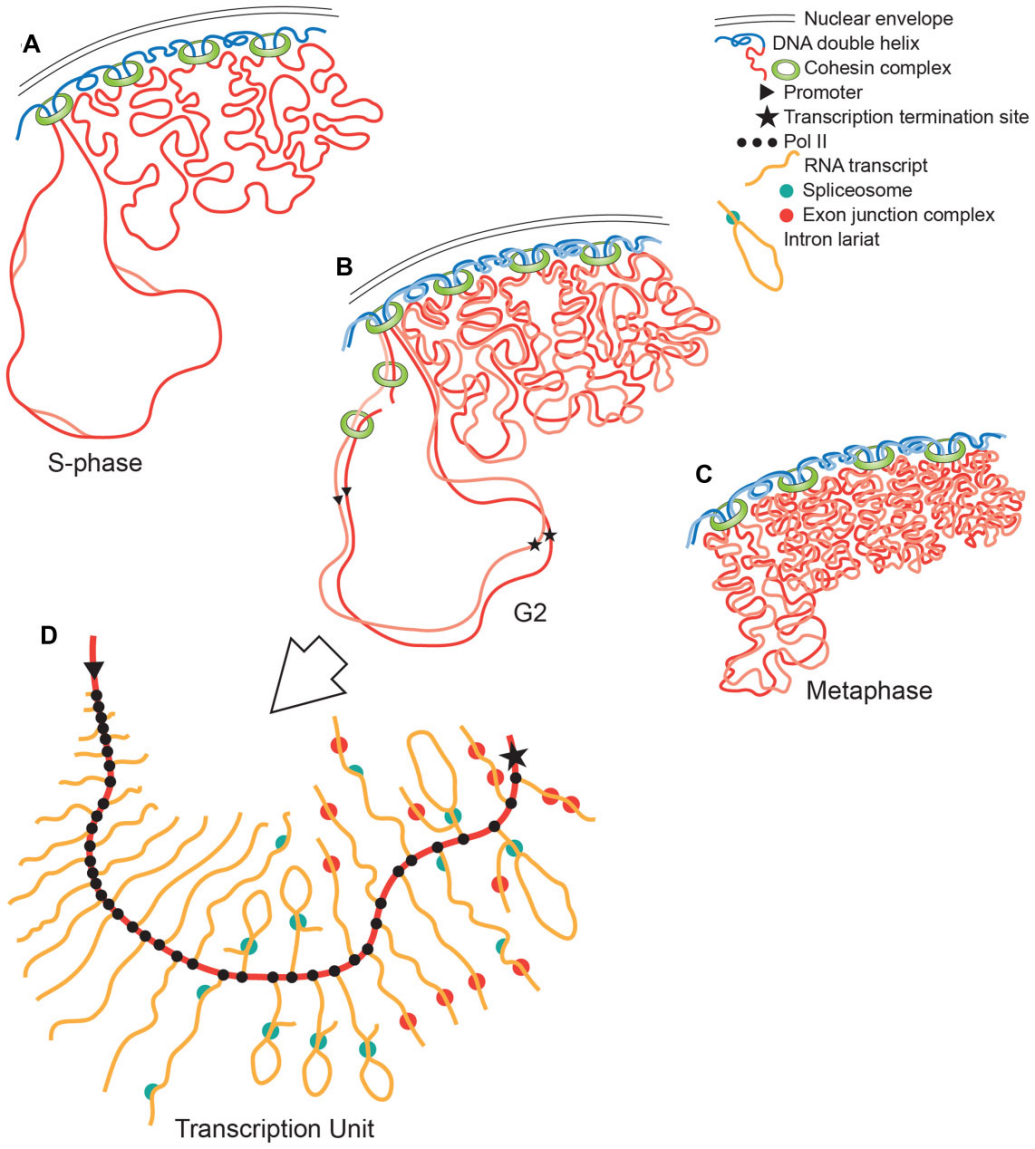
Diagram of eukaryotic chromosomes and TU organization. A, B, and C depict a small section of a chromosome during S-phase, G2 and metaphase, respectively. The nuclear envelope is represented by double black lines. Analysis of the Homo sapiens genome (Yuen and Gerton 2018) indicates that the cohesin protein complex (green) clamps the linear DNA molecule at specific DNA sequences to create looped DNA domains (red lines). In A and B the left loop is drawn extended to better show DNA replication and DNA breaks. Replication can initiate at multiple sites per loop (note replication bubbles in A and duplicated chromatids in B and C). B and C depict post-replication chromosomes, with sister chromatids indicated in different intensities of red. As indicated in B, cohesin also binds where DNA double-strand breaks occur (Caron et al. 2012). Electron micrographs of lightly-dispersed interphase chromatin (Supplemental Fig. S1) suggest that, during interphase, the chromatin strands are randomly folded, and they are depicted thus. In Miller spreads, paired sister chromatids, produced by DNA replication, remain in proximity (Fig. 2) and are so diagrammed here. C depicts metaphase. The nuclear envelope has broken down and the chromatin loops become more compacted than during interphase (cf. Fig. 6 vs. Supplemental Fig. S1). D diagrams transcription and RNA splicing of a two-intron TU whose promoter and transcriptional termination site are marked in B and D by a triangle and a star. Nascent transcripts are drawn in orange, the spliceosomes in turquoise and Pol II as small black dots. Transcripts elongate continuously as Pol II transcribes the underlying DNA, whilst spliceosomal components assemble at intron/exon junctions, and when two splice junctions come together, they clip out a “lariat” of intervening intronic RNA. As each intron is removed, another complex—the exon junction complex (shown as a red circle)—binds a little upstream of each exon–exon join. New transcripts are polyadenylated on their 3’ ends after release by Pol II, exported from the nucleus, and surveilled on the ribosome by the nonsense-mediated mRNA decay system to ensure that only transcripts without introns survive to be translated into protein.

**Fig. 6:**
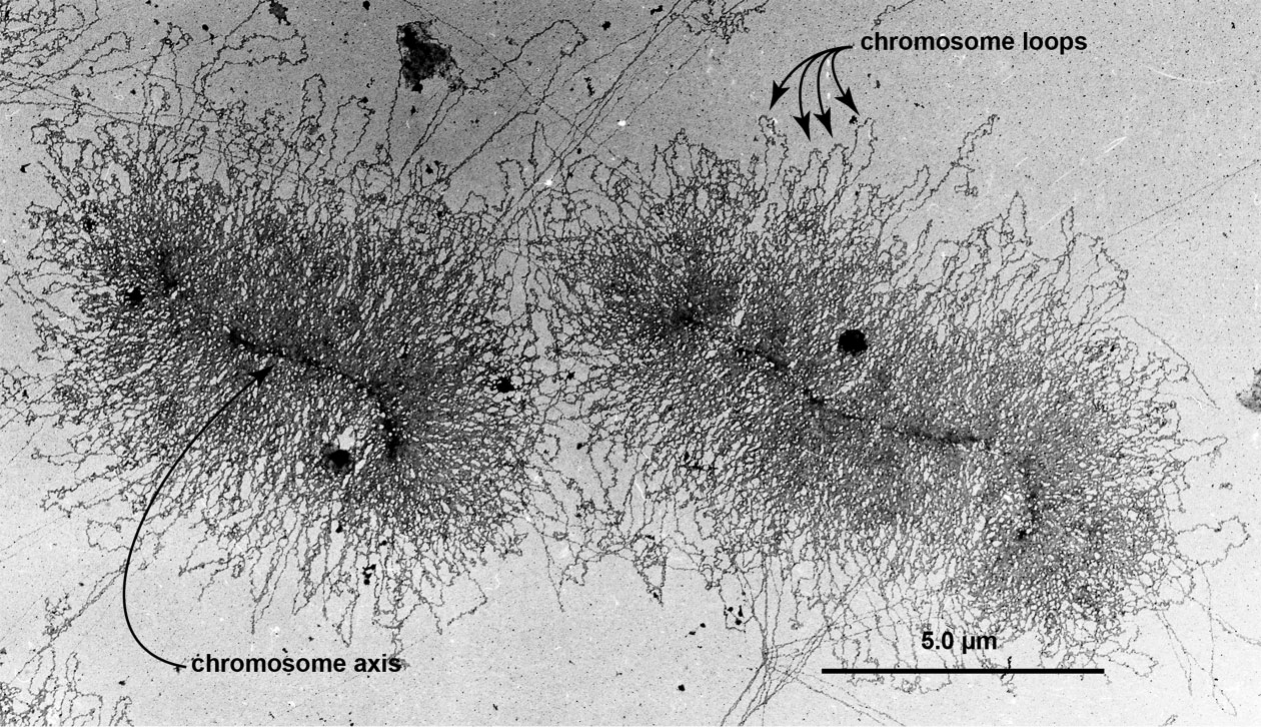
Condensed mitotic chromosomes showing loop domains and chromosomal axes. Electron micrographs of two anaphase holocentric chromosomes prepared from milkweed bug (Oncopeltus fasciatus) embryos. Chromosome loops protrude from the central axis, which stains densely with phosphotungstic acid. In Drosophila, humans and most eukaryotes, the spindle microtubules that at anaphase separate sister chromatids, attach to chromosomes at a single locus where kinetochore proteins assemble. In holocentric chromosomes, by contrast, kinetochore proteins are distributed along the entire chromosome axis. This provides multiple purchase points for spindle microtubule attachment, allowing even fragmented chromosomes to segregate during mitosis. Holocentric chromosomes have arisen multiple times in plants and animals, perhaps as an adaptation for surviving toxins, desiccation etc. that cause double-strand DNA breaks (Escudero et al. 2016), but they require special adaptations to segregate during meiosis—specifically, they reverse the order of meiosis I and II (Lenormand et al. 2016). Their protein-reinforced axes make holocentric chromosomes easier to prepare by the Miller protocol than, for example, Drosophila chromosomes, which tend to snarl during the centrifugation step. Unfortunately, this electron micrograph, taken 45 years ago, is at too low a magnification to allow a detailed view of the chromatin organization in the chromosome loops, which are thicker and more twisted than the beads-on-a-string 10 nm chromatin strands. Bar shows 5 micron.

The vastly larger eukaryotic genomes are partitioned into many chromosomes, with every chromosome being a single exceedingly long, linear molecule of DNA gathered into many large looped domains (Yuen and Gerton 2018). Fig. 5 summarizes the organization of the eukaryotic chromosome. The nuclear envelope from which eukaryotes take their name—eukaryote meaning “with a true nucleus”—prevents non-spliced RNA from premature exposure to the ribosomes in the cytoplasm, where the translation of mRNA into protein takes place. When eukaryotic cells exit interphase to divide, the DNA in the loops assumes a more condensed chromatin organization, transcription stops as Pol II dissociates from the chromatin, and in most eukaryotic species the nuclear envelope is temporarily disassembled (Fig. 5C). Fig. 5D illustrates diagrammatically the transcription, and transcript splicing, of a hypothetical two-intron TU, on a chromosome loop. Fig. 6 presents an electron micrograph of mitotic chromosomes showing the condensed looped domains, the chromosomal axis, and the absence of nascent transcripts on the condensed chromosome loops.

Using the estimate of 3.2 billion bp for the haploid human genome, the 19,116 TUs via which human proteins are encoded amount to 40 percent of our genome (Piovesan et al. 2019). At minimum, another five percent of the human genome is transcribed by Pol II into RNA transcripts *not* destined for translation into proteins (at least 4849 verified TUs; mean length 34,506 bp; Piovesan et al. 2019). The transcripts of some of these “RNA TUs” play roles in regulating gene expression, but the function of many others is still unknown (Statello et al. 2021). The entire 3.2 billion bp human genome is organized into about 10,000 heterogeneously-sized looped domains, partitioned between 23 unique chromosomes, and replicated from 30,000 to 50,000 replication origins (Méchali, 2010; Piovesan et al. 2019). *D. melanogaster's* genome is similarly organized, but is more compact, with 13,601 TUs in a genome about seven percent the size of the human genome (Adams et al. 2000; Hjelmen et al. 2019). Yeast genomes are even more compact—5–6,000 TUs in a genome just 0.4 percent the size of our own (Kupfer et al. 2004).

### The origin of eukaryotic introns

Where did the junk DNA that is now a feature of all eukaryotic genomes come from? Introns are believed to have evolved from Group II retrotransposons, which are RNA parasites found today in mitochondria, chloroplasts, and in some prokaryotes (Lambowitz and Belfort 2015). These virus-like entities encode in their tiny mRNA-like genomes information sufficient to ensure their own propagation. This includes a gene for reverse transcriptase, which enables the retrotransposon to violate molecular biology's Central Dogma and transcribe its RNA genome back into DNA. The retrotransposon presents itself for translation by the host's ribosome, and the reverse transcriptase enzyme that is made copies the retrotransposon RNA into DNA and pastes this DNA into the host genome. The host's RNA polymerases, in transcribing the host's own genes, may then inadvertently transcribe the inserted retrotransposon DNA. Critically important is the ability of the Group II retrotransposon RNA to fold into a complex three-dimensional configuration with a catalytic activity that precisely clips new copies of itself out of the host's transcripts. So long as a retrotransposon's inserted coding sequences remain intact, there is a good chance that the retrotransposon will cleanly excise itself from its host's transcripts, leaving behind functional host mRNA and a live host.

Unlike a classical virus, retrotransposons lack the protective capsid that helps a virus move between and infect new individuals. Thus, Group II retrotransposons are largely restricted to copying themselves into new DNA sites within the nuclear or organellar genomes of the cells they inhabit. When the host cell replicates its DNA, the cell's descendants are automatically infected.

There is a striking resemblance between Group II retrotransposons and the spliceosome, that nuclear organelle upon which so much of the eukaryotic transcriptional scheme rests (Lambowitz and Belfort 2015; Novikova and Belfort 2017; Vosseberg and Snel 2017). Eukaryotic spliceosomes include five small RNAs, which together form a three-dimensional structure similar to the retrotransposon's folded-up self-splicing RNA sequence; a catalytic Mg^2+^ sits at the core of both the retrotransposon and the spliceosomal RNA; spliceosomes and Group II retrotransposons use similar recognition sites and the same sequential esterification reactions to create the lariat intermediate, cut out the intervening (intron) sequence and rejoin the flanking (exon) sequences. In addition, a key spliceosomal protein (Prp8) and a homologous region in the Group II retrotransposon's reverse transcriptase form similar structures in their respective active sites. These similarities support the idea that eukaryotic introns originated from a genome-wide infestation of a eukaryotic ancestor by Group II retrotransposons (Koonin 2006).

Over time random mutations inevitably degrade encoded retrotransposon information, converting each integrated copy of retrotransposon DNA into a stretch of abandoned junk sequence, that is, into an intron. Parasitic DNA that has integrated into a genome and lost its self-splicing ability is not easily cast out. At some point, by appropriating a copy of the retrotransposon's RNA scissors and adapting them for independent use in *trans*, some pre-eukaryotic ancestor must have freed itself from having to depend on its parasites to excise themselves, one at a time, from the host's invaded RNA transcripts. Thenceforth, in its descendants, the processes that Fig. 4 illustrates—the precise excision of retrotransposon RNA and the suturing together of the host's own coding sequences back into functional mRNAs—were under host control. As a consequence, host survival became tied to maintaining efficient RNA splicing and effective splice-site recognition. To this end, it is hypothesized, those ancient cells recruited additional proteins and RNA molecules to stabilize and improve on the self-splicing catalytic RNA captured from the retrotransposon, cobbling together the huge and complex molecular machine that is the modern spliceosome (Collins and Penny 2005; Vosseberg and Snel 2017). Although present-day spliceosomes show some phylogenetic diversification, their overall similarity implies that the ancestor of all eukaryotes likely contained a spliceosome about as complex as that in existence today (Collins and Penny 2005).

When initially integrated into a host cell genome, each Group II retrotransposon with its self-splicing secondary RNA structure and an encoded reverse transcriptase would have been at least 3000 bp long. Modeling implies that the last common ancestor of fungi, animals, and plants carried between 3.7 and 5.1 of these insertions per 1000 bp of coding DNA—a formidable parasitic burden (Csuros et al. 2011). In present-day eukaryotes, different TUs differ greatly in length, often by orders of magnitude; this is almost entirely due to differences in the number and length of the intronic DNA sequences that each TU contains. Unequal crossing over during DNA repair, DNA replication slippage, and DNA transposition by mobile elements can alter intron lengths, move, and even completely eliminate introns. Such changes, occurring in reproductive cells, can be passed on, leaving the individuals carrying them subject to natural selection. Thus, junk DNA (introns) left behind from a long-ago Group II retrotransposon infestation came to be an integral part of eukaryotic genomes, providing a valuable tool for regulating transcription, as will be described below. However, two additional inventions were apparently needed first:

1. A nuclear envelope, which physically separates transcription and transcript splicing from translation. Retrotransposon invasion and proliferation may well have been the provocation that made it advantageous for the host cell to wall off newly transcribed RNA within a membrane-bounded nuclear compartment and away from ribosomes. Initially this barrier may have served just to allow time for excision of the retrotransposon's RNA before the host attempted to translate its mRNAs (Martin and Koonin 2006). The components of the nuclear pore complex and nuclear envelope appear to have come at least in part from repurposed prokaryotic molecules (Mans et al. 2004).
2. A nonsense-mediated mRNA decay system, which causes transcripts that have escaped the nucleus without being fully spliced to be destroyed rather than be translated. This system and the spliceosome may have evolved together during the transition to host cell control of RNA splicing (Lynch and Kewalramani 2003). The surveillance of intron removal is performed by a large multimolecular machine—the exon junction complex—which the spliceosome deposits on nascent transcripts during the process of splicing (Schlautmann and Gehring 2020). The exon junction complex binds 20–24 nucleotides upstream of wherever splicing creates an exon–exon join, and both it and the spliceosome remain bound to the elongating transcripts (visible in the Fig. 4 electron micrograph and diagrammed in Fig. 5D). The exon junction complex interacts with the nuclear pores to help draw spliced transcripts out of the nucleus, and as the RNA exits through a nuclear pore the bound complex promotes mRNA loading onto a ribosome. In these ways, intron processing stimulates mRNA expression. Most importantly, the ribosome uses the exon junction complex to detect and target for destruction those transcripts containing unspliced introns (Boehm and Gehring 2016). In a correctly-spliced transcript, each exon–exon join will be marked with an exon junction complex a little upstream of each splice site, and a single nonsense codon signifying translational termination will be located *distal* to the final splice site. Splicing failures result in mRNAs that include stretches of non-coding intronic sequence, which the ribosome detects by the presence of “premature” nonsense codons *upstream* of the last bound exon junction complex. These suspect transcripts are targeted for rapid destruction by nucleases while still on the ribosome (Lloyd 2018).

Since all eukaryotes possess these attributes, the last common ancestor of the eukaryotes most likely had introns, spliceosomes, a nuclear envelope, and the nonsense-mediated mRNA decay system, setting the stage for the spectacular achievements of its descendants.

I note that in addition to spliceosomal introns, eukaryotic genomes are rife with stretches of junk DNA left behind from successive waves of infestation by a variety of other self-propagating mobile genetic elements. These freeloaders or free agents do not appear to have played as foundational a role in eukaryogenesis as Group II retrotransposons have. However, depending on where they insert themselves into their host's genome, they can be evolutionarily consequential, adding length to intronic DNA, altering exons or regulatory DNA sequences, increasing the spacing between TUs, and even moving sequences from place to place within genomes (Burns 2020). Moreover, the presence of hundreds, or even thousands, of copies of such elements in a genome increases the likelihood of repair and replication mistakes, as I discuss below. Mobile genetic elements of all classes amount to about 20% of the *D. melanogaster* genome (Mérel et al. 2020). They add up to well above 50% of human genomic sequence; just one repetitive sequence known as the *Alu* element, with a copy number of over a million, comprises 10% of our genome and is present in at least 30% of human TUs, often in introns (de Koning et al. 2011; Daniel et al. 2015).

### Introns provide a versatile tool for regulating mRNA production

Almost from the moment introns were discovered, it was understood that a selective use of alternative splice sites provides a way for one TU to encode many variants of a single protein (Gilbert 1978). That is, by evolving mechanisms that allow one type of cell to omit from a TU's mRNA one or more exons included in the mRNA produced from the same TU by another cell type, organisms can fine-tune proteins for better performance in different tissues, organs, and circumstances (Graveley 2001). For example, different splice variants of the gene encoding alpha-tropomyosin regulate contraction in smooth *vs.* striated muscle (Ruiz-Opazo and Nadal-Ginard 1987). The TU encoding the Down's syndrome cell adhesion molecule (DSCAM) provides an extreme demonstration of the capability of this system. DSCAM encodes cell surface receptors critical for axon guidance during nervous system development in animals as diverse as fruit flies and humans. The combinatorial use of a very large number of alternative splice sites makes it possible for one TU to generate over 38,000 distinct cell-specific DSCAM homodimers, well over twice the total number of unique genes encoded in the entire *Drosophila* genome (Schmucker et al. 2000; Hattori et al. 2008). Even *S. pombe*, a unicellular yeast with only short introns, uses regulated alternative splicing to create protein variants (Awan et al. 2013). In extant eukaryotes alternative splicing is controlled by a system of trans-acting regulatory proteins (Chaudhary et al. 2019; Jordan et al. 2019; Shenasa and Hertel 2019; Ule and Blencowe 2019).

Much less widely appreciated is the important regulatory consequences of TU length *per se.* Since, when eukaryotic cells pass from interphase into mitosis, RNA polymerase II (Pol II) dissociates from DNA, the transcription of each TU must be initiated anew at the start of each interphase (Shermoen and O'Farrell 1991; Hartl et al. 1993; Gottesfeld 1997). As a result, the total length of a TU (introns plus exons) determines the minimum time required for that TU to produce its first mRNA molecule, thence protein. The requirement that Pol II transcribe long stretches of junk DNA therefore serves as a *de facto* timing fuse for gene expression during each cell cycle ( Hogness et al. 1985; Gubb, 1986; Thummel 1992). RNA elongation rates have been measured at 1–3 kb/min in *Drosophila*, and 1.3–4.3 kb/min in humans. (That large range is likely a consequence of non-uniform distributions of transcriptional obstacles—bound proteins and hard-to-open stretches of DNA sequence, and their cumulative effect on Pol II progress.) TUs with short introns can be transcribed in well under a min. On the other hand, it takes 80–90h to transcribe the 4,300,000 bp long-TU that encodes *Drosophila* male fertility factor kl-3 to produce kl-3 mRNA (Fingerhut et al. 2019). Male fertility factor kl-3 encodes dynein heavy chain, an essential component of the sperm tail motor complex, which is encoded by 14,000 bp of that enormously longer transcript. As a reference, the *Drosophila* TU shown in Fig. 2B would take at least an hour to transcribe. The x-axis in Fig. 3 shows the *minimum* time required to transcribe the variously-sized *human* TUs.

During short cell cycles, the fact that Pol II must read through lengthy stretches of intervening junk DNA reduces the total *amount* of mRNA that a long TU can produce, sometimes to none at all. Suppose that, beginning at the same moment, and continuing throughout interphase, RNA polymerases load onto different-length TUs at the same rate. When mitosis brings transcription to an end, many more full-length mRNA copies will have been made from short TUs than from otherwise identical long TUs. Even in long-duration cell cycles, when there is enough time for many complete passages of Pol II along a lengthy TU from transcriptional initiation to termination, an inverse relationship will exist between TU length and the number of mRNAs produced. This relationship will continue until well after the longest active TU has been transcribed. Only in cells with a sufficiently long interphase will a steady state be reached where the transcription rate of each TU is instead set by transcriptional initiation.

It is therefore not surprising that the most abundantly transcribed TUs—presumably belonging to genes whose products are needed in large amounts—are those with short or no introns (Castillo-Davis et al. 2002; Eisenberg and Levanon 2003; Jeffares et al. 2008). On the other hand, sequence analysis of the 5’ and 3’ ends of the transcripts produced in early *Drosophila* embryos shows that, in general, it is the *inability* to completely transcribe long TUs, rather than an absence of transcriptional initiation, that restricts early embryonic mRNA expression to short TUs (Artieri and Fraser 2014). Study of three other *Drosophila* species, which diverged from *D. melanogaster* roughly 12, 45, and 63 million years ago, show TU length playing this same role—*preventing* TUs from expressing their mRNAs during early embryogenesis (Artieri and Fraser 2014).’

The redundant gap genes, *knirps (kni)* and *knirps-related (knrl)* are required for abdominal segmentation in *Drosophila.* These, and an intron-less transgene for *knirps-related*, demonstrate the relation between TU length and expression timing (Rothe et al. 1992). The TUs for *knirps* and *knirps-related* are 3 kb and 23 kb long, respectively. The knirps protein is expressed during the *Drosophila* embryo's 13th nuclear cycle, but *knirps-related* is too long to be completely transcribed during this cycle's approximately 12 min long interphase, making the shorter, but otherwise redundant, *knirps* gene essential. However, a short intron-less transgene for *knirps-related* can substitute for a deletion of *knirps* (Rothe et al. 1992).

In *Drosophila* all six of the male fertility factors (*kl-1; ks-1; kl-2; ks-2; kl-3; and kl-5*) share an analogous TU structure in containing enormous introns composed of repetitive DNAs (Gatti and Pimpinelli 1983). Their lengths, orders of magnitude longer than the average TU ensure that mRNA production from these particular TUs is withheld until late in the atypically long prophase that characterizes meiosis I, which precedes spermatid differentiation when the proteins that these TUs encode are needed (Fingerhut et al. 2019).

The multicellular bodies of eukaryotes are built by complex gene networks, where the relative timing of protein expression in gene activation cascades is often critical. For example, as Gubb and Hogness were the first to point out, the large sizes of homeobox TUs (e.g., *Ultrabithorax*—76,000 bp and *Antennapedia*—100,000 bp) delay the production of homeobox proteins until they can impose segment identity on a fruit fly embryo already partitioned into segments by the smaller, earlier-expressed TUs of the gap, pair rule and segment polarity gene networks (Hogness et al. 1985; Gubb 1986). Pol II takes over an hour to transcribe the *Ultrabithorax* TU (Shermoen and O'Farrell 1991), which encodes a 1500 amino acid transcriptional regulatory protein. Ultrabithorax mRNA does not begin to be expressed until late in the embryo's 14th interphase, after cellularization of the previously syncytial embryo is complete, in the newly formed cells of the presumptive third thoracic and first abdominal segments. It controls in a cell-specific manner whether adult flies will develop wings or halteres (Akam and Martinez-Arias 1985).

In TUs with identical promoters, the inclusion of different-length timing fuses allows a single control molecule to activate a cross-regulatory gene expression cascade. Consider the *Drosophila* genes, *E74A* and *E74B*, whose promoters are activated simultaneously in the larva by a systemic pulse of ecdysone. The mRNA for each gene appears in a burst, but offset in time, and with delays expected from the time needed to transcribe their respective 20 kb and 60 kb long TUs (Karim and Thummel 1992; Thummel 1992). These two early-expressed members of the ecdysone gene network then activate downstream targets, and they are eventually turned off by the product of yet another ecdysone-activated TU, one whose expression timing is almost certainly set by the length of its own timing fuse. Yet for nearly an hour after its promoter shuts off, *E74A* continues producing transcripts, as expected given its 60 kb length (Karim and Thummel 1992). In Fig. 2B that same circumstance is visible: the replicated TU lacks upstream transcripts, indicating that transcriptional initiation has ceased, while many transcripts have been left to continue their long journey towards the termination site.

Sometimes the expression level of a gene is controlled by a direct negative feedback mechanism in which an increased concentration of the gene's own protein product feeds back to reduce, or stop, transcriptional initiation of the TU that produced it. The previously loaded RNA polymerases will then continue producing mRNA long after the promoter shuts down, introducing a time delay (proportional to the length of the TU) into this type of negative feedback. Delays of appropriate duration can produce temporal oscillations in protein concentration, and Takashima et al. (2011) demonstrated that precisely such a mechanism regulates mesoderm segmentation in mice. In that case, Hes7 protein represses transcription from the *Hes7* promoter, which in turn down-regulates *Hes7* mRNA and Hes7 protein levels; in mouse embryos this auto-inhibitory feedback produces oscillations of Hes7 protein concentration with a two-hour periodicity. Deletion of introns in the *Hes7* TU abolishes this oscillation, and causes severe defects in somite segmentation (Takashima et al. 2011).

As another gene network example, sequence analysis of the RNA transcripts produced in serum-starved human fibroblasts in an immediate response to serum stimulation has an effect on about 1000 TUs, 4/5ths being activated and 1/5th deactivated, with 12% of the newly activated TUs being transcriptional regulators (Kirkconnell et al. 2016, 2017). The serum-activated TUs that encode transcriptional regulators differ in length such that their respective mRNAs appear over two or more h. Such length differences, together with cross-regulatory interactions analogous to those described above for ecdysone-activated TUs, allows the single triggering event of serum exposure to unleash a complex and long-lasting cascade of patterned protein expression.

Using alternative splice sites to generate multiple protein variants from a single TU seems clever and is metabolically frugal. By contrast, it might seem inelegant and bizarrely wasteful to use transcription of enormous lengths of junk DNA as a regulatory device. However, as we have seen, junk DNA length easily and robustly provides fine-scale regulation of the *timing* of mRNA expression *within a cell cycle*. It may be much more difficult for interactions between proteins and nucleic acid molecules to produce so subtle and precise a clock. Moreover, transposon insertions, as well as replication and repair mistakes, provide a constant source of intron length variation for natural selection to try out, so that fine-tuning the within-cycle timing of gene expression by intron length change can be readily accomplished. Furthermore, using transcription itself as a regulatory device means that, despite changes in temperature, ATP levels, RNA precursor abundance etc., the *relative* timings and amounts of different mRNA species with respect to one another will remain constant.

In summary, TU activation and the rate of transcriptional initiation are governed at the promoter, in eukaryotes and prokaryotes alike. The addition of introns gave eukaryotes both alternative splicing and a simple way to regulate the timing of gene expression within cell cycles—two devices that can facilitate the construction of elaborate genetic circuitry. As the examples provided show, these tools have been deployed to create complex multicellular bodies. Clearly, the Eukarya have long since thoroughly incorporated their ancient genetic parasites, and natural selection, ever the inventive opportunist, has put their left-behind DNA carcasses to ingenious use.

### The remarkable intron-position conservation in transcription units

Probabilistic Markov modeling of the intron/exon layout of 245 orthologous TUs (i.e., TUs evolved by descent from a single ancestral TU), in 99 extant eukaryotes, indicates that the genome of the last common eukaryotic ancestor must have been intron-rich, with an intron density *higher* than many current-day eukaryotes (Stajich et al. 2007; Csuros et al. 2011). Further intron gains occurred, some coinciding with the emergence of major plant and animal groups, most notably with the appearance of multicellular animals—the Metazoa (Carmel et al. 2007). But more generally, lineage-specific intron *losses* have predominated and many eukaryotes, unicellular eukaryotes in particular, are now relatively intron-poor (Roy 2006; Csuros et al. 2011a; Rogozin et al. 2012).

The long-ago Group II retrotransposon invasion presumably left introns in DNA positions of no particular value to their hosts. Even after the host cells acquired the ability to clip retrotransposon RNA out of their own transcripts, purifying selection would have gradually eliminated lineages where integrated retrotransposons caused inappropriate mRNA production. By the same token, when introns happened to be in positions that set the timing of individual gene expression in an advantageous way, such lineages would have prospered. Nonetheless, it is astonishing how many introns now occupy positions that appear to have remained unchanged for 1.5—2 billion years (Carmel et al. 2007). Pairwise alignment of 30 TUs with a conserved intron/exon structure in extant eukaryotes (i.e., 30 TUs for which 44% of introns in plants and animals are in the same position) shows 39% of these positions are shared by extant fungi too (Fedorov et al. 2002). A larger study analyzed 684 orthologous TUs (and 21,434 introns) from eight sequenced eukaryotes—*Homo sapiens*, two insects, a nematode worm, a plant, two yeasts and the malaria-causing protozoan, *Plasmodium falciparum* (Rogozin et al. 2003). Many intron losses and some gains are plainly evident. But 25–30% of intron positions in the plant and the vertebrate orthologs match, as if they had been inherited from their last common ancestor. Although *Plasmodium* has a low intron density, a third of its introns occupy TU positions shared with humans. Another study comparing 1590 orthologs in humans and mice revealed that over the course of 90 million years no intron gains occurred and just 5 out of the 10,020 introns examined had been lost (Roy et al. 2003). The intron losses (all in mice) were exact and the exons flanking the lost introns remained intact.

Analyses by Koonin and colleagues of orthologous TUs in 19 eukaryotic species concluded that the vast majority of shared intron positions result from genuine evolutionary conservation. “An intron present in the last common ancestor of the Metazoa has a probability of 0.83 to be retained in humans, whereas an intron present in the last common ancestor of multicellular life has a probability of 0.57 to be retained in extant plants.” (Carmel et al. 2007).

Why, in orthologous TUs, would so many introns have remained in the same position during hundreds of millions of years of evolution? In the simplest cases of intron excision, spliceosomes join all of a TU's exons in the same order in which they occur in the TU, whilst in TUs that undergo alternative splicing, specific splice-sites may simply be skipped over in certain circumstances, thereby excluding specific exons from the final mRNA. Either way the resultant mRNA nucleotide sequence depends on the sequential order of exons in the TU. Thus, the conservation of intron/exon positions almost certainly reflects the importance of preserving similar mRNA sequences so as to encode analogous proteins.

DNA addition or removal, confined to the junk DNA of the introns, expands or contracts TU lengths, introducing variations in timing and levels of mRNA production that natural selection can act upon. Note also that whereas, in principle, a single long intron in a TU suffices to correctly set the timing and level of gene expression, if the same total length of junk DNA is interspersed between multiple exons, it is less vulnerable to accidental loss since that would require multiple independent accidents. In a competition run for billions of years, during which losing the ability to correctly regulate gene expression disqualifies the contestant, the “genes-in-pieces” organization appears to have been especially adept at staying in the race.

### Intron lengths change in response to selection

Comparison of TUs in *Homo sapiens*, *D. melanogaster* and *C. elegans* reveals systematically longer introns in humans than in the two invertebrates (see figures on page 333 in Alberts et al. 2015). This implies that intron *lengths* are sufficiently consequential that natural selection tunes them, although the selective forces at work almost certainly vary by species.

Studies of TU structure in mice and humans show a high degree of conservation of *exon* length and base sequence, and as just explained, of the positions of introns within orthologous TUs. Additionally, there is a striking tendency for the *relative lengths* of orthologous TUs in these two mammals to be conserved. For example, 68% of human TUs are about 1.5 times longer than their mouse counterparts (Batzoglou et al. 2000; Kirkconnell et al. 2017). Conserved length ratios between orthologous TUs may result from natural selection acting to preserve *relative* gene expression timings in analogous gene networks, when the organisms in question have different mean cell cycle lengths.

Current-day unicellular yeasts have far shorter and fewer introns than what has been inferred for ancestral fungal taxa (Deutsch and Long 1999; Csuros et al. 2011). In these unicellular eukaryotes, selection against numerous and long introns was likely driven by the advantages of genomic streamlining to facilitate rapid proliferation. In Appendix II, I discuss bdelloid rotifer genomes, where, in closely-related species, a greater exposure to DNA breakage seems to have resulted in genomes with dramatically shorter TU lengths (Nowell et al. 2018).

Fugu, the smooth pufferfish (*Takifugu rubripes*) has the most compact vertebrate genome known (365 million bp)—less than half the size of that of its relative, the spiny pufferfish *(Diodon holocanthus*), from which it has been diverging for 50–70 million years (Guo et al. 2010). Their different genome sizes result from both intron and intergenic DNA length differences, with addition and deletion of multiple varieties of transposable elements accounting for the differences (Guo et al. 2010). What has driven the differences between these two types of pufferfish remains mysterious. But in general, transposon invasions will tend to drive genome expansions, which may be selected against due to any of several species-specific constraints such as cell-cycle length, gene regulatory tasks, and life-style or habitat limitations.

### Introns create a great vulnerability

Regardless of how, or even whether, eukaryotes make use of their transcribed junk DNA, the existence of long TUs is indisputable (e.g., Fig. 3). Their length, their interspersed exon/intron organization, and the use of one TU to encode several alternatively-spliced variants, make eukaryotic TUs highly vulnerable to double-strand breaks (breaks where both strands of the DNA double helix are severed). An unrepaired break *anywhere in a TU* prevents processive RNA polymerases from reaching downstream exons, so the longer a TU the more vulnerable is its mRNA. To make matters still worse, the probability of a break occurring also increases with TU size: the longer a TU, the larger a target it is for ionizing radiation, attack by free radicals, a destructive collision between DNA and RNA polymerases, the leading strand DNA polymerase reading across a single-strand nick at a replication fork, and the many other commonplace and largely unavoidable events that can sever a DNA molecule (Mehta and Haber 2014). The afore-mentioned nonsense-mediated mRNA decay system, present in all eukaryotes, targets for destruction improperly terminated transcripts, usually eliminating the truncated transcripts that broken TUs produce (Chang et al. 2007; Hug et al. 2016; Nickless et al. 2017). Yet whether truncated transcripts read from severed TUs are destroyed by nonsense-mediated mRNA decay, or persist to be translated into incomplete and nonfunctional proteins, the result is that a TU break, unless repaired correctly, is likely to render a TU incapable of making its intended mRNA, hence protein.

Why focus on double-strand breaks given that TUs are also damaged by mutations (changes in base sequence caused by the intrinsic chemical instability of nucleotides, exposure to carcinogens, DNA replication and repair mistakes etc.)? The answer stems from quantitative considerations. The complete sequencing of trios (mother, father, and child) reveals that each human baby is born with between 50 and 100 new DNA base pair changes, with half contributed by each parental gamete (Sasani et al. 2019). Thus, the haploid human genome, consisting of 3.2 billion bp, is acquiring new base changes in germline cells at a rate of less than two per year. The vast majority of these will have no effect because they will occur in noncoding DNA, and only a small fraction of random mutations (less than 1 percent in humans) will occur in the coding DNA. Even within the coding DNA, because of the redundancy of the genetic code, many will not alter amino acids. Of those that do, 27–29% of base changes have been found to have no effect on the function of the resulting protein, 30–42% are only mildly deleterious, and just 30–45% fall into the highly deleterious to lethal class (Boyko et al. 2008). Thus, the point mutations that arise *de novo* during an organism's lifetime only rarely change an encoded protein enough to impair its function.

The situation with double-strand breaks is strikingly different. In human somatic cells from 10 to as many as 50 double-strand breaks occur *every cell cycle* (Vilenchik and Knudson 2003). In the 40% of the human genome that is devoted to protein-encoding TUs (Piovesan et al. 2019), any unrepaired double-strand break will ruin an encoded protein, and in TUs with alternative splicing, *all variants of said protein*. The additional five percent of the human genome that encodes long non-translated RNA molecules (Piovesan et al. 2019) is presumably equally vulnerable to breaks. Moreover, a break anywhere in a TU's non-coding DNA is exactly as disruptive of mRNA production as if the break had occurred in the most critical exon. For the Eukarya to have added enormous lengths of junk DNA to their TUs, however, useful those additions may be, seems therefore phenomenally dangerous. The remainder of this essay explores how eukaryotes appear to have solved this conundrum and the very far-reaching implications of their solutions.

### Redundancy is an antidote to entropic information loss

Continuation of each life form depends critically on preserving and passing on its treasury of encoded genetic information, and minimizing entropic deterioration of the genome is a major cell occupation. The elegance of the double helix as a repository for information lies in allowing the many accidental lesions that afflict only one strand of the double helix to be excised and returned to their former state by using the redundant information encoded in the complementary strand to guide elaborate sets of DNA repair enzymes (Chatterjee and Walker 2017). DNA breaks that completely sever the double helix present cells with a much more difficult repair challenge. Yet, left unrepaired, double-strand breaks pose enormous problems for the eukaryotic chromosome during cell division, as I now explain.

Fig. 5 depicts a tiny segment of a eukaryotic chromosome. Immediately prior to the start of each new interphase, the evolutionarily-conserved cohesins (green rings), organize anew the very long DNA molecule that is each chromosome (*red* lines) into a series of looped domains (Li et al. 2020). Cohesins, together with the interloop DNA (*blue* lines), form the axis or backbone of each chromosome. Any break in the axis DNA will fragment the chromosome. A chromosome fragment cut free from the chromosomal centromere by a DNA break cannot segregate normally at mitosis, which failure produces daughter cells with either supernumerary and/or missing chromosome pieces, with cell death being the usual outcome for such cells. On the other hand, a double-strand break in a looped DNA domain (*red* lines), if it occurs in a TU, will abolish mRNA production from that one TU. In dividing cells, after sister chromosomes segregate at anaphase, the cohesins form an ATP-driven DNA-encircling sliding clamp and reassemble the loops from linear DNA molecules by extrusion. This means that breaks anywhere in the DNA of what will become a looped domain, are also likely to produce fragmented chromosomes, with the same deadly consequence as axis breaks. At best, they may merely do so one cell cycle later. It is not surprising then that somatic cells have evolved mechanisms sensitive enough to detect even a single DNA break and to arrest cell cycle progression, allowing time for repair (Lydall et al. 1996; Lee et al. 2000, 1998; Abraham 2001). Eukaryotes have one pathway—homologous recombination—that can *accurately* repair double-strand breaks. In addition, they have three end-joining repair pathways that can restore chromosomal integrity (and that in that way are highly beneficial rescue mechanisms), but which may fail to return chromosomes, and TUs, to their original layouts (Mehta and Haber 2014; Iliakis et al. 2019; Zhao et al. 2020; Stinson and Loparo 2021).

*Homologous recombination* can execute seamless, error-free repair of double-strand breaks, but to enable the DNA fragments to rejoin correctly, this pathway requires a nearby duplicate chromosome copy to use as a template (Haber 2018). This requirement relies on the ability of cells to create from the DNA on either side of the break a probe that can actively search the welter of nearby DNA double helix for potential sequence complementarity—a miraculous feat if there ever was one (Bell and Kowalczykowski 2016; Haber 2018). First, nuclease enzymes produce a stretch of single-stranded DNA (more than 100 bp) at the end of each broken piece of DNA. Next a RecA-type protein found in every life form in which it has been sought, intertwines and coats the single-stranded DNA (RecA is the protein's name in the bacterium, *E. coli*; Rad 51 is the usual name of the eukaryotic RecA ortholog in somatic cells; and Dmc1 is the eukaryote's meiotic ortholog). Stable base pairing between the filaments of RecA-coated single-stranded DNA and a complementary strand of intact double-stranded DNA then aligns the two DNA fragments with the intact template, allowing DNA polymerase to synthesize new complementary strands that seamlessly and accurately patch the break or gap (Mehta and Haber 2014).

In general, accurate repair by homologous recombination is cell cycle dependent. Dividing cells progress through three successive phases of interphase: G1, S, and G2, before division. **G1** is a cell's variable length **firstgrowth** phase; once cells attain a certain size, they trigger DNA **synthesis** (**S**-phase). During S-phase, DNA synthesis creates a duplicate copy of every chromosome. After S-phase, cells enter a shorter **secondgrowth** phase (**G2)**, before they undergo **mitosis** (**M**-phase), when they divide. Terminally differentiated cells cease dividing and arrest in a modified G1 known as G0. The accurate repair of double-strand breaks by homologous recombination is effectively restricted to between late S-phase and when sister chromatids separate during M-phase, that is, to the time when identical sister chromatids are present for use as repair templates (Johnson and Jasin 2000; Kass and Jasin 2010; Mazón et al. 2010).

Miller spreads of cell cycle 14 *Drosophila* embryonic chromatin prepared anytime during an approximately 1.5 h long G2 (21°C) reveal identical sister chromatids usually lying near one another (Fig. 2; also McKnight and Miller 1979). Their proximity means that in the event of a double-strand break, a RecA-coated probe should be able to discover the homologous template quickly. Furthermore, cohesin has been shown to assemble *de novo* on chromatin abutting a double-strand break (Caron et al. 2012), and depleting cohesin promotes tumorigenesis (Leiserson et al. 2015; Woodward et al. 2016). This DNA clamp may aid accurate break repair by helping to hold broken chromosomes together, as suggested by Fig. 5B.

In organisms with diploid somatic cells, the two homologous chromosomes, one inherited from each parent, could in principle also serve as repair templates for one another. However, yeast studies show that recombination between homologs during mitotic cell cycles is inefficient due to the homolog often being too far away (Lee et al. 2016; Haber 2018). In Miller spreads of embryonic *Drosophila* chromatin, one almost never sees four identical TUs in proximity (a rare exception is shown in Supplemental Fig. S2). This suggests that in *Drosophila*, homologous alleles are unlikely to be reliably close enough for a RecA homology search to find them, at least during brief embryonic cell cycles.

### Double-strand breaks must often be repaired using pathways that can alter chromosomal organization

When a break occurs during G1, before DNA replication, cells cannot readily use homologous recombination for repair because there is no sister chromatid to serve as a template. As G1 is the longest duration phase of the normal cell cycle, and also because most terminally-differentiated somatic cells arrest in G0 (i.e., before DNA replication), eukaryotes must frequently resort to one of several less precise end-joining repair pathways to salvage broken chromosomes.

*Nonhomologous DNA end-joining* is the predominant eukaryotic break repair pathway. It is much faster than homologous recombination and operates throughout the cell cycle, although it is down-regulated during and after DNA replication (Symington and Gautier 2011; Chapman et al. 2012; Zhao et al. 2020; Stinson and Loparo 2021). This is a catch-as-catch-can method of patching, which ligates broken DNA ends back together directly, with no or very little sequence homology requirement. If the two ends of a break have not diffused apart, non-homologous end-joining is likely to rejoin broken chromosomes quickly and correctly, although this pathway usually adds or deletes a few bases in squaring up the ends for ligation (Zhao et al. 2020; Stinson and Loparo 2021). Mutations in the non-homologous DNA end-joining genes are associated with tumorigenesis, presumably because the fallback is break repair by more error-prone pathways, to be described next (Sishc and Davis 2017). However, in cells where neither of those alternative end-joining repair pathways is available, non-homologous end-joining itself can be tumorigenic. This is probably because, if multiple breaks are present at the same time, this pathway occasionally rejoins sequences that had not been contiguous, causing chromosomal reorganizations.

Bringing ends back together to be rejoined, once they have diffused apart, poses a major difficulty for cells. The two other end-joining repair pathways, which must be used when sister chromatids are not available, rely on direct annealing between complementary sequences (not RecA-type homology searching) to align broken strands (Iliakis et al. 2019; Zhao et al. 2020). *Microhomology-mediated end-joining* chews back one strand of the DNA flanking either side of the break to produce short (less than 20 bp) single-stranded DNA ends. *Single Strand Annealing* creates somewhat longer stretches of single-stranded DNA (50–100 bp). Microhomology-mediated end-joining and Single Strand Annealing both use their single-stranded ends as bait to find complementary sequences to which to reconnect, but complete the process of ligation via different pathways. In Single Strand Annealing, the DNA between the region of homology and the break site, sometimes many thousands of base pairs long, is simply cut out and discarded (Symington and Gautier 2011; Decottignies 2013). Microhomology-mediated end-joining repair takes over when homologous recombination and non-homologous end-joining are suppressed and it is favored during DNA replication (Leeman et al. 2019). It is unclear whether the single-stranded ends anneal only to other already broken ends, or also to unbroken single-stranded DNA made accessible by DNA replication, which would make S-phase an especially dangerous time to undertake repair. Regardless, because genomes are littered with short direct and inverted sequence repeats, double-stranded break repair pathways that rely on direct annealing can easily reorganize genomes. Indeed, repetitive sequences and microhomologies commonly mark sites of break mis-repair (Bentley 2004; Leeman et al. 2019).

When two breaks are present simultaneously, end-joining repair may flip the orientation of a piece of one chromosome (an inversion), or switch chromosome pieces between chromosomes (a translocation), and/or eliminate a stretch of a chromosome (a deletion). Yet, so long as every chromosome ends up with two telomeres and one centromere, the ability of chromosomes to replicate and to segregate during mitosis has been restored. Thus, even when it fails to recreate the original chromosome organization, and it eliminates one or several genes, end-joining break repair is usually far less damaging to somatic cells than leaving breaks unrepaired. However, occasionally end-joining repair does create chromosomes with no centromere at all and chromosomes with two centromeres. In dividing cells, the former leads to gene dosage problems and usually to cell death. The latter can beget breakage-fusion-bridge cycles, which inflict new genome wreckage with new each round of cell division. This sort of genomic instability predisposes cells to malignancy, so not surprisingly the low-fidelity end-joining repair of double-strand breaks is a well-established risk factor for cancer (Bunting and Nussenzweig 2013).

### Chromosome rearrangements tend to destroy transcription units

If a DNA breakpoint happens to fall within a TU, *any end-joining process that produces a chromosomal rearrangement will in most circumstances destroy that TU* by separating its promoter-proximal and promoter-distal halves. Such a “repair” will have made it impossible for processive Pol II to read the entire TU so as to produce the intended mRNA, and it will therefore deprive that cell and its descendants of the protein this TU encodes (including also all splice variants thereof).

The background level of double-strand breakage in non-dividing primary cultures of human fibroblasts is about 10 breaks per day. Following exposure to ionizing irradiation, 40–60% of newly induced breaks were found to be repaired within the first 30 min, over at least a 100-fold range of irradiation dosages (Rothkamm and Lobrich 2003). Those breaks that are mended rapidly are probably those where the broken ends have not diffused apart and where ligation will restore the original chromosomal organization. However, 2 h later 15–20% of irradiation-induced breaks had still not found a broken end to ligate to, although most *will* eventually be repaired (Rothkamm and Lobrich 2003). Should another break occur in the same nucleus before the ends have found their original and correct partner, these unrepaired breaks have the potential to create chromosomal inversions and translocations. With 10 random new breaks per day—one on average every 2–3 h—the slow-healing breaks would seem to have a substantial chance of rejoining in other than their original position. Breakpoint analysis of 18 large balanced non-tumorigenic inversions in human subjects showed that 62% of those had resulted from non-homologous end-joining, confirming that this pathway does in fact create chromosomal rearrangements (Pettersson et al. 2020).

Study of the rate at which chromosomal rearrangements accumulate in the kidney cells of living mice as they age, and of the rate at which breaks appear in primary cultures of mouse or human fibroblast cells, led to the calculation that by age 70, *every cell* in a human body will have undergone on average 2,300 double-strand break repairs made by some pathway *other than accurate homologous recombination* (Lieber and Karanjawala 2004). This was estimated to be equivalent to one in every 430 genes in every diploid cell having suffered a low-fidelity repair. That 1/430 estimate was based on the assumption that the *diploid* human genome contained 50,000 genes, that what was potentially susceptible to break damage was just the coding DNA, and that this amounted to 5% of the genome. Using the updated knowledge that not 5, but 45% of the human genome is vulnerable to break damage (Piovesan et al. 2019), and that there are, not 50,000, but 2 × 19,116 = 38,232 TUs per diploid genome, a revised estimate is that by age 70 one in every 37 genes will have undergone low-fidelity repair. Assume for the moment that non-homologous end-joining produces no chromosomal rearrangements but only alters sequences around the break site. In the 99 percent of the human genome that is non-coding this should have negligible consequences. But, base-changes in the one percent of the human genome that corresponds to exons can potentially ruin encoded proteins; given the organization of the human genome, by age 70, on average ten exons in every diploid cell will have been altered in this way. Moreover, in dividing cells, this damage may well be orders of magnitude greater (see box 2 in Lieber and Karanjawala 2004).

However, a far more serious threat to genomes is end-joining repair that causes chromosomal rearrangements, which can occur when two unrepaired breaks are present simultaneously. As already explained, inversions and translocations with break points within a TU completely wreck the ability of that TU to produce its intended mRNA. If these correspond to even one percent of the breaks that fail to re-ligate rapidly (Rothkamm and Lobrich 2003), in a typical human cell these would produce a chromosomal rearrangement at least once every hundred days, a substantial fraction of which would be expected to have permanently destroyed a TU. This is an estimate. The actual rate at which translocations and inversions form, especially in dividing cells, is a critical issue that is now amenable to experimental determination with recently developed DNA sequencing techniques.

While no DNA-based life form can be immune to double-strand breaks, many things affect an organism's vulnerability to them: among these are genetic specifics such as the collective length of TUs in that organism's genome, the abundance of repeat sequences, and the level of cell ploidy, as well as species particulars such as the number of cells per organism, the dominant break repair pathway, the level of exposure to DNA-damaging environments, and whether the organism has rigid cell walls (as plants, algae, and fungi do), or tissues prone to damage by metastasis (as metazoans do). As examples, note that spontaneous double-strand breaks in yeast arise at about the same rate per mega base of DNA as in mammals, but in yeast with its small genome that translates into just 1 per 8 cell divisions (Haber 2018). Using rates of cancer as a proxy for rates of double-stranded breaks, environmental effects are illustrated by age-standardized rates of cancer in Australia being nearly 1.5 times those in Great Britain, due to exposure of a non-native light-skinned population of predominantly British origin to solar UV (Bray et al. 2018); UV damage is repaired by a pathway that tends to lead on to double-strand breaks. Furthermore, because the chances of a DNA strand breaking increase with its length, it is not surprising that a correlation exists between especially long TUs and several multigenic human diseases, including cancer (Sahakyan and Balasubramanian 2016). Regardless of these details, the key message of this section is that eukaryotic TUs are subject to destruction by end-joining mis-repair, and most especially to mis-repair that produces chromosomal rearrangements.

In purely somatic cells, chromosomal rearrangements that ruin individual TUs may put the survival of individual organisms at risk, but they do not become part of their species’ gene pool. By contrast, the same repair mistakes in germline cells can be passed from one generation to the next, potentially posing a cumulative, species-level existential danger. Given the large fraction of a eukaryotic genome that is devoted to TUs, and the incidence of breaks and unavoidable mis-repair, it is staggering to imagine the irrevocable TU ruination after ten thousand, or half a million years of cumulative damage transmitted through the germline. Gene destruction progressing at anywhere near the rate seen in somatic cells, and accruing generation upon generation, would soon demolish all prospects of maintaining functional organisms.

To consider how eukaryotes may have solved the erosive problem of DNA breakage, and the mis-repair thereof, we turn next to sexual reproduction.

### The great enigma of sexual reproduction

Why sexual reproduction is adaptive has been an abiding puzzle to biologists (see, for example, Williams 1975; Maynard Smith 1978; Bell 1982; Weismann 1889; Barton and Charlesworth 1998; Otto 2009;Lenormand et al. 2016). Considering the Eukarya in their entirety, including the huge numbers of unicellular individuals, most instances of reproduction probably occur *asexually*, by mitosis. However, the majority of eukaryotic species that routinely reproduce asexually do resort to sex, they just do so infrequently. Conversely, in multicellular species that usually procreate sexually, when mates or opposite-mating type individuals are unavailable, or if an opportunity for especially rapid population increase presents itself, quite a few can temporarily turn to asexual reproduction. Yet, notwithstanding the very considerable additional cost and complexity it adds to life histories, remarkably few eukaryotes have abandoned sex altogether.

It is notable too that over evolutionary timespans most asexual species dwindle and vanish sooner than sexual species (e.g., Beck et al. 2011). On the Tree of Life, virtually all asexual taxa sit at the tips of twigs, having not given rise to new branches (Bell 1982). Sexual reproduction must be fundamentally important for the continuance of life, and also for the formation of new species. But why and how?

I begin by briefly reviewing two non-exclusive hypotheses discussed in the literature for the adaptive value of sexual reproduction. The first I believe is correct but incomplete. The second I believe correctly identifies what the first misses, but as I will explain, is not a solution that eukaryotes can use because of the structure of their chromosomes.

1. As explained earlier, mutations appear very slowly, but when occurring in germline cells they can be passed on. As a consequence, over time, eukaryotic populations come to have in circulation many slightly different variants of their genes—“alleles”. Lethal and highly deleterious alleles are removed from a species’ gene pool when the individuals carrying them perish or fail to thrive. However, mildly deleterious or suboptimal alleles often cannot be eliminated in this way, and Muller's Ratchet is the name given to the generation-on-generation accumulation of low-quality alleles, which, unless they can somehow be gotten rid of, inevitably degrade chromosomes, genomes, and organisms (Muller 1964; Kondrashov 1988). In organisms that reproduce sexually, recombination between homologous chromosomes during meiosis creates hybrid chromosomes (part maternal/part paternal). Since maternal and paternal homologs often carry different alleles, recombination, followed by random segregation of each member of a homologous chromosome pair, and then random segregation of chromatids, means gametes receive thoroughly reshuffled genomes. Every new diploid zygote then inherits—via gametes from its two parents—a never-before-seen mix of alleles. By chance, some zygotes will be handed an excess of defective alleles which in sum cripple their prospects. Others may find themselves by luck dealt a hand ideally suited to the circumstance into which they are born. Those fortunate ones have temporarily slipped from Muller's snare, even as their sibs carry their sets of maladaptive alleles to the grave. Thus sex, by continually reshuffling the alleles that a species has in circulation, ensures genetic variation, and this is critical for exposing deleterious alleles in new combinations to purifying selection (Felsenstein 1974; Kondrashov 1988; Rice 2002). It also improves the odds that some offspring will inherit alleles especially suited to the unpredictable and heterogeneous world in which each new generation finds itself (Bell 1982). Think of a species’ gene pool as a swarm of interacting genes, flying through time, constantly shedding deleterious alleles but retaining newly mutated ones when these enhance survival, always in flux, adapting dynamically to the environment through which the swarm is passing by its shifting allelic composition. That species-level outcome emerges from individuals doling out different allele combinations to offspring. Without doubt this bet-hedging and constant adjusting is one benefit of sexual reproduction, and it plays an essential role in evolution.
2. I have emphasized how vulnerable eukaryotic TUs and chromosomes are to double-strand DNA breaks. This issue forms the basis for an altogether different explanation for the persistence of sex, one laid out by the Bernsteins and their colleagues in a succession of papers beginning in the 1980s (Bernstein et al. 1985, 1987). They argued that the forerunner of eukaryotic sexual reproduction is “transformation”, that energy-requiring process during which a bacterial cell *in extremis* actively takes in exogenous DNA and incorporates stretches of the acquired DNA into its own genome (Bernstein et al. 2012, 2018). Using the homology-locating ability of RecA and homologous recombination, bacterial survival is increased under circumstances that cause double-strand breaks. Archaea use transformation to overcome break damage too, and some even actively recruit conjugal partners. For example, hyper-thermophilic species of the order Sulfolobales have a UV inducible system of filament formation that promotes species-specific cell aggregation and DNA transfer, while at the same time increasing recombination rates by as much as three orders of magnitude (Fröls et al. 2008; Ajon et al. 2011; Bernstein and Bernstein 2017). I have already described how, in the somatic cells of eukaryotes, homologous recombination can seamlessly repair double-strand breaks when sister chromatids are available to serve as repair templates. Bernstein and colleagues proposed that in an analogous manner, during meiosis, homologous chromosomes serve as repair templates for one another. They saw recombination between homologs as indispensable for break repair, and the reshuffling of alleles that this may produce as an occasionally-beneficial side-effect (Bernstein et al. 1988). The crux of their argument was that double-strand breaks are such an existential problem for DNA-based life that virtually all eukaryotes must at some point resort to meiosis or else risk not being able to leave viable offspring, and that sexual reproduction has been retained since the dawn of the Eukarya primarily for DNA break repair (Bernstein et al. 1988, 2011, 2012, 2018).

What I hope to convince the reader of is that to solve the Eukarya's very serious double-strand DNA break problem, meiosis is required for doing something other than what Bernstein and colleagues propose, something almost more mechanistically astonishing than homologous recombination. Redundancy is still key, but this time not solely to guide repair. To understand my proposal, I first briefly describe meiosis.

### The courtship, very complicated marriage, and separation of homologous chromosomes

Meiosis is the evolutionarily-conserved heart of sexual reproduction. It encompasses the standard textbook process whereby a diploid cell, with two sets of replicated homologous chromosomes (one of maternal and one of paternal origin) produces haploid gametes, each with a single set of chromosomes. Meiosis requires two consecutive nuclear divisions: meiosis I aligns and partitions homologous chromosomes, while meiosis II separates and partitions sister chromatids. It is on the intricate prelude to the first of these nuclear divisions that I wish to fix attention.

During interphase of meiosis I, gamete precursor cells—meiocytes—replicate their DNA and then in a protracted prophase bring their duplicated homologous chromosomes into side-by-side alignment. Whereas DNA replication automatically produces perfectly aligned, side-by-side sister chromatids (Fig. 5A and B), homolog alignment is a feat that can take days in animals and weeks in plants (Zickler and Kleckner 1999). Homologs are helped to locate one another by an assortment of different species-specific cytological behaviors. For example, meiocytes in many plants, animals, and yeasts slosh, jiggle, jerk, or wave their chromosomes about early in prophase of meiosis I, increasing the odds that repetitive sequences in centromeres, nucleoli, telomeres, or special-purpose pairing centers will touch and anneal. Ciliates, on the other hand, force their homologs into proximity by squeezing their meiotic nuclei into long snake-like cylinders, while keeping the ends of their chromosomes anchored to opposite poles of the elongating cylinder (Zickler and Kleckner 1998; Alleva and Smolikove 2017).

A precise side-by-side alignment of the homologs is subsequently brought about as a conserved meiotic protein (Spo11) inflicts round after round of double-strand DNA breaks on the prophase chromosomes (Keeney 2008). Depending on the organism, anywhere from several hundred to several thousand such lesions may be produced per nucleus (Page and Hawley 2004). An intimate alignment is then driven by RecA's meiotic orthologs (Rad51 or Dmc1), which create probes from the ends of the broken DNA strands that search nearby chromosomes for complementary nucleotide sequences (Cole et al. 2010; Harrison et al. 2010; Zickler and Kleckner 2015).

**Fig. 7:**
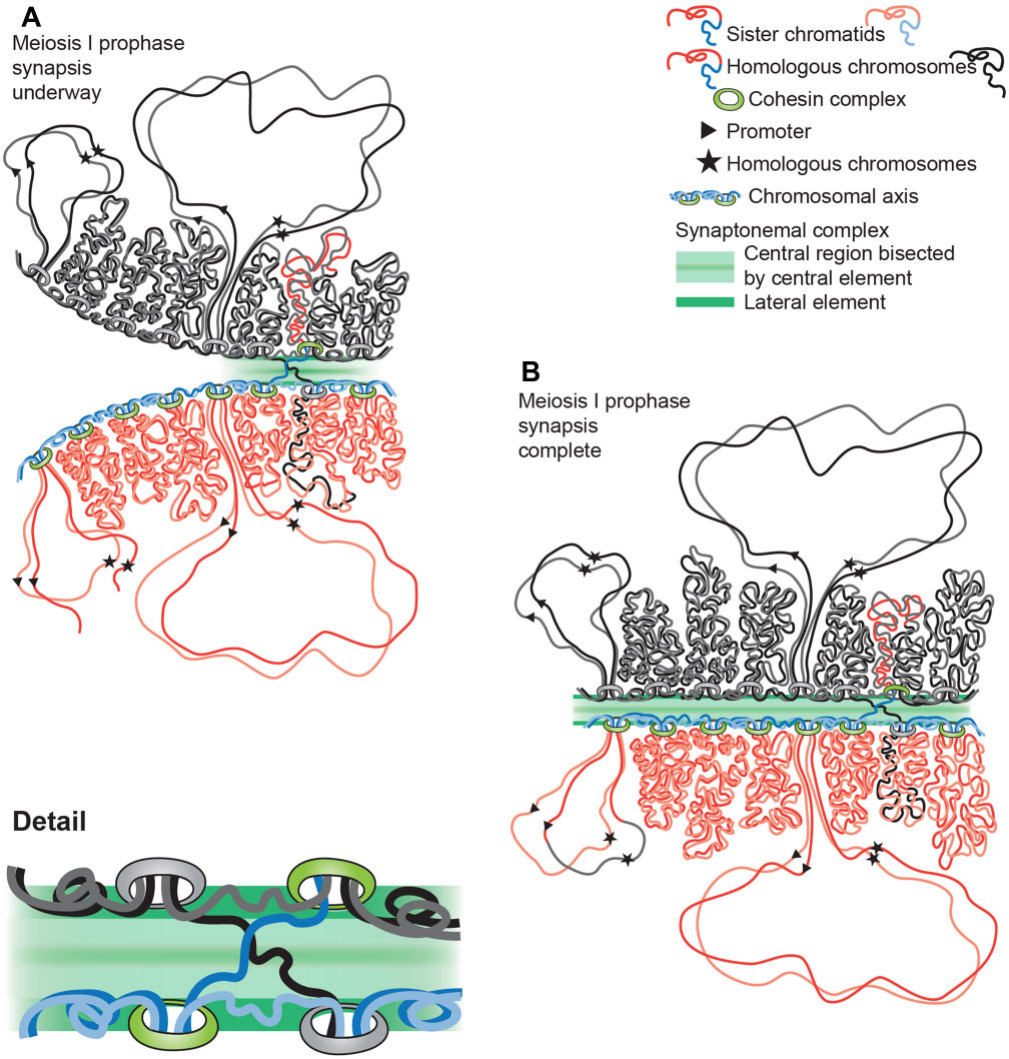
Synaptonemal complex formation. Chromosomes are represented as in Fig. 5; the two chromatids of one homolog are depicted in black & grey, and those of the opposite homolog in two red tones. The stars and triangles in A and B signify that during meiosis I some chromosome loops may be transcriptionally active. For example, primary spermatocytes in humans and fruit flies, primary oocytes in amphibians and some plants all have chromatin loops bristling with transcripts, hence the name—lamp brush loops. Indeed, it is in primary spermatocytes that Drosophila's longest TUs are transcribed (e.g., the several million bp male fertility factors; Fingerhut et al. 2019). Homolog synapsis is slowly brought about as, over a lengthy period, each meiocyte inflicts double stand breaks upon its own chromosomes and these are slowly repaired by homologous recombination, using the homologs as reciprocal templates. The DNA break shown in the lower left-most loop in A is depicted in B as having been repaired by gene conversion. In A and B, a break that has already been repaired and which was resolved as a crossover between homologs is depicted on the right-hand arms of the paired homologs. As breaks are occurring and being repaired, the synaptonemal complex (shown in tones of green) is forming between the paired homolog axes (A), gradually welding the two homologs together along their entire lengths (B). See main text for a description of the synaptonemal complex structure, depicted in the Detail.

For break repair to align homologs requires an accumulation on the chromosomal axes of meiotic HORMA domain-containing protein(s). The HORMADs, which take their name from three members of that protein family—**Ho**p1p, **R**ev7p, and **MAD**2—function as signal-responsive adaptors that undergo a major conformational change to mediate protein-protein interactions (Rosenberg and Corbett 2015; Vader 2015). During prophase of meiosis I, the HORMAD(s) bind cohesins (which define the base of the chromosomal loops—see Fig. 7) to other meiosis-specific proteins, and this assemblage on the chromosomal axes focuses the Rad51/Dmc1 homology search on homologous chromosomes (as opposed to sister chromatids). As a consequence, the DNA homology-based repair of the Spo11-inflicted double-strand breaks gradually brings homologous chromosome pairs into sequence defined, side-by-side alignment (species-specific reviews in Kim et al. 2014; Subramanian an Hochwagen 2014; Hong et al. 2019; Fujiwara et al. 2020; Grey and de Massy 2021; West et al. 2018).

**Fig. 8:**
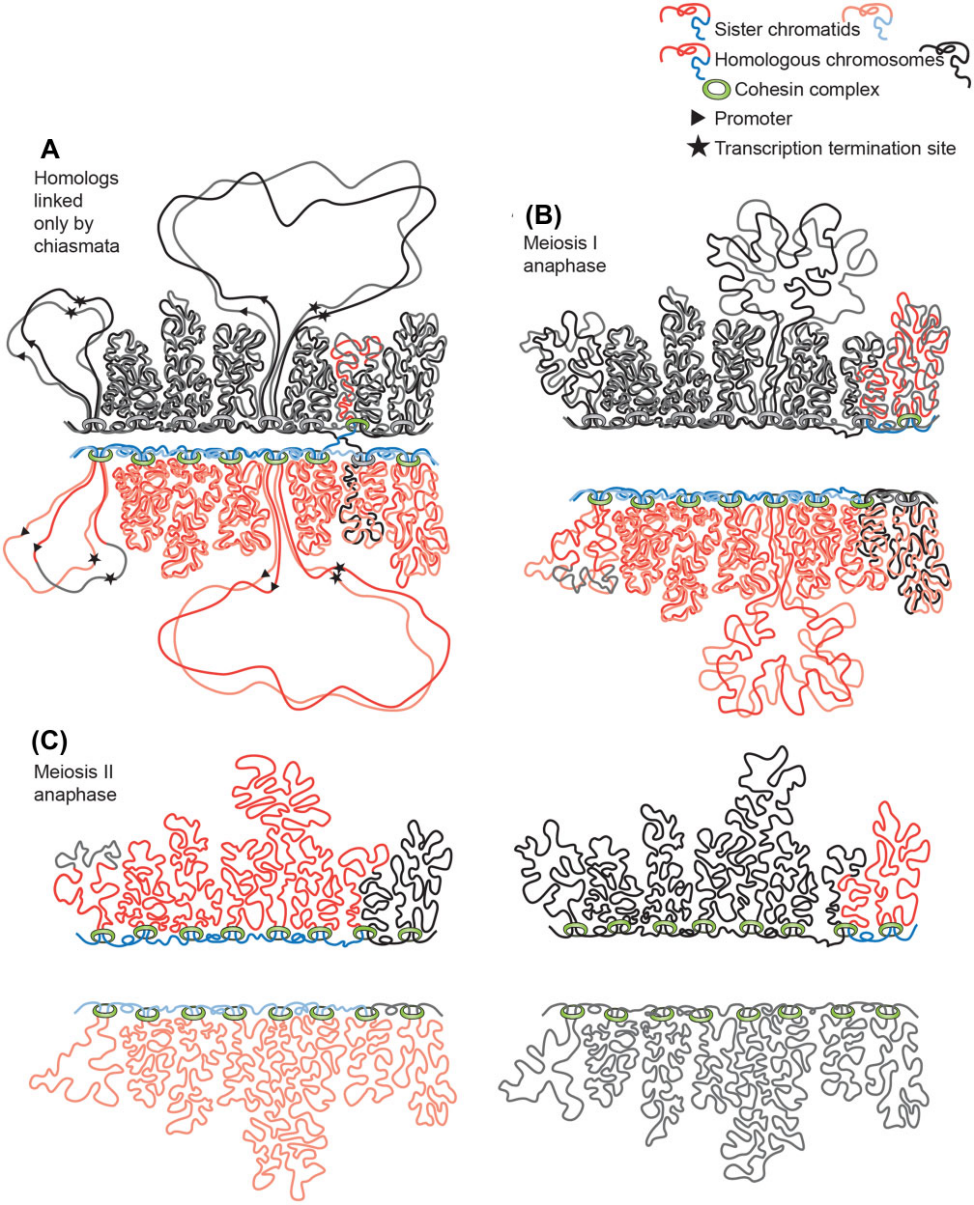
Genome partitioning during meiosis I and II. Chromosomes are represented as in Fig. 7. After the synaptonemal complex depolymerizes (A), and until anaphase of meiosis I, homolog pairs are held together by chiasmata (the physical crossover between homologs). When, at anaphase, homologs do move apart, each crossover resolves into a reciprocal exchange of a subset of looped domains (B). During anaphase of the second meiotic division the cohesin rings “open” (not shown), allowing the sister chromatids to separate from one another; immediately thereafter, cohesins create the looped domains anew (C). Due to the gene conversion and reciprocal crossovers that occurred during prophase of meiosis I (7A and B), individual chromatids now carry reshuffled combinations of alleles (8C).

The majority of the Spo11-induced double-strand breaks are repaired in such a way as to leave small patches of newly synthesized DNA copied non-reciprocally by DNA polymerase from the opposite homolog, a process known as “gene conversion”. However, at least one break per chromosome, is always resolved so as to create a reciprocal exchange between segments of the paternal and maternal homolog—a crossover (Page and Hawley 2004; Zickler and Kleckner 2016; Haber 2018). This obligatory crossover temporarily locks the homologs physically together, creating a linkage that is essential for the orientation and segregation of the two homologs away from one another at metaphase/anaphase of meiosis I. Fig. 7 illustrates the two alternative outcomes of break repair: gene conversion and reciprocal crossover. Fig. 8 diagrams the consequences of these two types of repair for the genetic makeup of the gametes that meiosis II will produce.

As the homologous chromosome pairs are slowly being aligned by breakage and repair, a singular meiotic structure—the synaptonemal complex—gradually forms between the pairing homologs (Page and Hawley 2004; Zickler and Kleckner 2015; Cahoon and Hawley 2016). The synaptonemal complex *per se* consists of a three-layered protein structure (represented in shades of green in Fig. 7 detail). As homologs are brought into alignment, two “lateral elements” form in contact with the chromosome axes and become interconnected by a ladder of transverse filaments that span halfway across the complex to overlap, zipper-like, in the electron-dense mid-region known as the central element (Page and Hawley 2004; Zickler and Kleckner 2015; Dubois et al. 2019; West et al. 2019). The looped DNA domains of each homolog protrude laterally from opposite sides of this synaptonemal complex (Fig. 7A and B).

Once all of the homolog pairs are aligned and synapsed, and the homolog crossovers are completed, the HORMADs proceed to dissociate from the chromosomal axes, triggering synaptonemal complex disassembly. Unlocking a HORMAD from the paired homologs requires an AAA-ATPase, the **p**achytene **ch**eckpoint factor (PCH-2 in *C. elegans*, PCH2 in plants and *Drosophila*, Pch2 in *S. cerevisiae*, and TRIP13 in mammals). The displacement of the HORMADs from the chromosomal axes provides critical information to the cell cycle regulatory machinery, and licenses the meiocytes to exit pachytene and resume cell cycle progression (Joyce and McKim 2010; Deshong et al. 2014; Subramanian and Hochwagen 2014; Argunhan et al. 2017; Tsubouchi et al. 2018; West et al. 2018; Roelens et al. 2019; [Bibr bib12][Bibr bib244]). As I discuss shortly, this key cell cycle checkpoint, which depends on both the meiotic HORMAD(s) and Pch2/PCH2/PCH-2/Trip13, is central to the proposals in this essay.

Depolymerization of the synaptonemal complex leaves the homologs linked only by the crossovers that recombination created (Fig. 8A), while freeing their chromatids to serve as templates for sister–sister double-strand break repair (as they do normally). The crossovers mature into “chiasmata” that will continue holding the homolog pairs together through metaphase of meiosis I, a length of time that in the oocytes of long-lived female mammals (such as humans) can be as long as 50 years.

During anaphase of meiosis I, the two homologs separate, each having incorporated a stretch of chromosome from the opposite homolog (8B). Subsequently, at anaphase of meiosis II, the sister chromatids separate (8C). Due to crossover recombination, the random segregation of individual members of each homolog pair at meiosis I, and then random segregation of sisters at meiosis II, the gametes that are produced inherit well-shuffled sets of alleles. Thus are the cards dealt for that high stakes, once-in-a-lifetime game of chance described earlier—from which each new zygote draws a mediocre, terrible, or extraordinary hand of alleles—and natural selection (i.e., real life) decides winners.

The synaptonemal complex is a multi-tasking molecular machine that—like the spliceosome, the nuclear envelope, and the exon junction complex—is an ancient invention dating from eukaryogenesis. Its current-day functions include setting the relative abundance of the two different products of inter-homolog recombination (crossovers *vs.* gene conversions), controlling the number and distribution of crossovers along each chromosome, DNA base mismatch detection and repair, and conveying the state of homolog synapsis to the cell cycle machinery. In many organisms these functions are mechanistically linked, so that mutants that affect one of these processes often affect the others (e.g., Roeder and Bailis 2000; Page and Hawley 2004; Joyce and McKim 2009; [Bibr bib78][Bibr bib279]).

Although the synaptonemal complex's tripartite organization is a conserved feature, in different taxa this complex can be constructed from quite different proteins that contain conserved functional domains (Fraune et al. 2012, 2013; Grishaeva and Bogdanov 2014; West et al. 2019). Given the great antiquity of this structure, this divergence is not particularly surprising. Nor is it surprising that in different species the basic functions outlined above may be carried out in slightly different ways, or that they have become integrated with different species-specific or sex-specific molecular pathways.

### The synaptonemal complex creates the pachytene checkpoint

The quality surveillance mechanism known as the *pachytene checkpoint* is made possible by the formation and subsequent dissolution of the synaptonemal complex*.* This proof-reading checkpoint slows or blocks exit from the pachytene stage of meiotic prophase when meiotic recombination or chromosome synapsis are incomplete, or when chromosomal rearrangements are present as heterozygotes (San-Segundo and Roeder 1999; Roeder and Bailis 2000; Bhalla and Dernburg 2005; Mitra and Roeder 2007; Joyce and McKim 2009, 2010; Subramanian and Hochwagen 2014; Bohr et al 2016; Cahoon and Hawley 2016; Tsubouchi et al. 2018). For example, if one homolog has deletions, duplications, translocations or inversions that the other homolog does not have, the homolog pairs may fail to synapse fully. The checkpoint then arrests cell cycle progression at the pachytene stage of meiosis I, and in many organisms this checkpoint goes on to trigger a programmed cell death (apoptosis) of the arrested meiocytes. In mice, for example, a reciprocal X-autosome translocation causes synapsis failure during prophase of meiosis I, and the pachytene checkpoint causes the translocation heterozygotes to be efficiently culled (Odorisio et al. 1998).

A second, distinct and critically-important meiotic checkpoint ensures that the DNA breaks introduced to align the homologs are not transmitted to the gametes. Meiocytes with unrepaired DNA breaks are prevented from progressing to metaphase of meiosis I (Bhalla and Dernburg 2005; Wu and Burgess 2006; Bolcun-Filas et al. 2014). The meiotic DNA-damage checkpoint involves many of the same proteins that create the canonical DNA-damage checkpoint that operates in mitotic cells. However, whereas the mitotic checkpoint merely causes a lengthy cell cycle delay (Lee et al. 1998, 2000), the meiotic DNA damage checkpoint induces meiocyte death.

The remainder of this essay concerns the pachytene checkpoint and its relevance to mis-repaired double-strand DNA breaks, with a focus on the checkpoint's organismal and species-level consequences.

Meiocytes that give rise to eggs are known as oocytes. Primary oocytes are in meiosis I, secondary oocytes in meiosis II. In *C. elegans*, primary oocytes in pachytene—the stage when the synaptonemal complex is fully formed—are hyper-resistant to DNA breakage by X-rays. This is not true of the same primary oocytes before synapsis, nor of oocytes after the synaptonemal complex dissolves, nor of early embryonic cells (Takanami et al. 2000). *C. elegans* encodes only one ortholog of RecA (Ce-rad-51), which is expressed at high levels in primary oocytes and is up-regulated after X-irradiation. Resistance to X-rays is lost when Ce-rad-51 is silenced by RNAi (Takanami et al. 2000), implicating the RecA pathway in double-strand break-repair during pachytene. As in mice, the pachytene checkpoint arrests and preferentially destroys by apoptosis primary oocytes in which synapsis of every homologous chromosome pair has not occurred (Bhalla and Dernburg 2005; Bohr et al. 2016). Using a different apoptosis-inducing pathway, the meiotic DNA damage checkpoint subsequently destroys primary oocytes with residual DNA double-strand breaks, preventing them from continuing on to meiosis II (Bhalla and Dernburg 2005).

As outlined previously, Harris Bernstein and colleagues have long argued that the primary adaptive function of sexual reproduction is repair of DNA breaks by homologous recombination using maternal and paternal homologs as mutual templates for repair (Bernstein et al. 1985). As the experiments of Takanami and colleagues in *C. elegans* beautifully confirm, extant breaks, whether self-inflicted by Spo11, or caused by exogenous agents, can indeed be readily repaired during meiosis (Takanami et al. 2000). Moreover, during synaptonemal complex formation, homologous recombination switches from using sister chromatids to using homologous chromosomes as repair templates. So, Bernstein et al. are correct about the *capabilities* of meiocytes. However, most, if not all, of the breaks undergoing repair during meiosis were produced by Spo11 during a preparatory step in homolog synapsis. It seems highly unlikely that double-strand breaks caused by the normal wear and tear of cellular life can persist unrepaired through multiple mitotic cycles and reach meiosis as breaks. This is because the structure of eukaryotic chromosomes is such that unrepaired breaks put cells at risk of losing chromosome pieces during mitotic cell division, a loss apt to cause cell death. As previously explained, mitotically-dividing cells arrest cell cycle progress when a break is detected, and they devote four different break repair pathways to ensuring that breaks do not go unrepaired. When repair by homologous recombination is not feasible, and when broken DNA ends have diffused apart, thus eluding rapid and correct non-homologous end-joining, multiple breaks can accumulate. When this occurs, eventual repair may reorganize chromosomes. Therefore, germline cells are unlikely to reach meiosis with unrepaired DNA breaks, but they may well present with chromosomal inversions, translocations, and sizable deletions due to inaccurate repair.

### The synaptonemal complex, by assessing whether homologous chromosomes are laid out identically, makes it possible for organisms to selectively eliminate those gametes most likely to have lost genes due to faulty break repair

What is the adaptive value of a dedicated checkpoint that arrests the development of, and in many cases proceeds to actively kill, meiocytes with defects in recombination, synapsis, or with chromosomal rearrangements present as heterozygotes? One of the things that the synaptonemal complex regulates is inter-homolog crossovers, and a failure to produce at least one crossover between every homolog pair leads to nondisjunction, and therefore to aneuploid gametes. Thus, the standard explanation is that the pachytene checkpoint, by winnowing out meiocytes with improperly paired and recombined homologs, reduces the creation of aneuploid progeny (Bhalla and Dernburg 2008; Joyce and McKim 2010; Subramanian and Hochwagen 2014; Zickler and Kleckner 2015; Cahoon and Hawley 2016; Dubois et al. 2019; Pyatnitskaya et al. 2019). However, building something as elaborate as the synaptonemal complex with its pachytene checkpoint for this purpose alone seems excessively extravagant, since individual organisms that are missing large portions of their genomes, or that conversely carry either supernumerary chromosomes or chromosome pieces, are likely to die promptly in any case. This is especially true of haploid organisms, which is what early eukaryotes are thought to have been.

I suggest that there is something else important, something requiring much more finesse, that the pachytene checkpoint is also doing to protect genomes for the long-term success of each type of organism: ***itisselectivelyeliminatingthosemeiocytesinwhichamis-repaireddouble-strandbreakislikelytohavedestroyedatranscriptionunit****.* In this way, the pachytene checkpoint helps ensure that intact genomes are preferentially propagated from one organismal generation to the next.

As explained earlier, large deletions, and chromosome rearrangements—inversions and translocations—are the molecular signature of prior double-strand breaks that have been “repaired” by end-joining that failed to return chromosomes to their original layout. Inversions and translocations destroy TUs by separating what had been one continuous TU into disconnected promoter-proximal and a promoter-distal pieces. Central to my proposal is the fact that—as laid out in an early part of this essay—the cumulative lengths of eukaryotic TUs mean that, for an organism like a human, at least 45% of this class of mis-repairs will have wrecked a TU. Inversion and translocation heterozygotes therefore flag meiocytes in which a mis-repaired double-strand break has a significant chance of having deprived that gamete-producing cell of at least one specific type of mRNA, hence protein.

Depending on species, gamete-producing cells embarking on meiosis may be newly recruited from an undifferentiated cell lineage (as, for example, in many plants and algae), or come from a dedicated germline (as in many animals including *Drosophila* and *H. sapiens*). Break mis-repairs may be newly inflicted (such as by ionizing radiation during a cancer treatment), or have been accumulating over many consecutive life cycles (as occurs in dividing yeast cells or in intermittently parthenogenetic organisms, such as aphids). Regardless, during prophase of meiosis I, in each gamete-producing cell, the organization of two juxtaposed homologs, procured from two different individuals, are compared. If complete synapsis, indicative of matching homolog organization, cannot be achieved, the pachytene checkpoint can safely “conclude” that one of that cell's chromosomes at some prior time lost its original organization, implying that one of its many TUs may have been destroyed due to DNA breakage followed by faulty repair. Rather than risk creating gametes likely to lack one or more genes, the pachytene checkpoint preferentially prevents such meiocytes from creating gametes.

To summarize, in eukaryotes, DNA breaks are the most dangerous form of information loss, are exceedingly common, and their occasional mis-repair is unavoidable. The failure of a homolog pair to fully synapse during meiosis is a way to detect chromosomal rearrangements, and is therefore an indirect means by which to identify those gamete-producing cells likely to have lost genes to inaccurate double-strand break repair. A cell has no way to know which member of a homolog pair is carrying the incorrectly mended TU, so it blocks the further development of, or kills outright, meiocytes with mis-matched homologs.

I submit that the synaptonemal complex, and the pachytene checkpoint it creates, constitute a filter that lets meiocytes whose chromosomes retain their original structural layout contribute to gamete production, while screening out those that bear the hallmark of gene-destroying mis-repair. This helps ensure that long, intron-laden, and easy-to-break eukaryotic TUs can be faithfully passed on. Without the pachytene checkpoint, eukaryotes could not have made such an expansive use of the introns that our ancestors filched almost 2 billion years ago from retrotransposons—a use that has contributed greatly to the evolution of complexity in eukaryotes. Minimizing chromosomal rearrangements has the added benefit of preserving for future generations the constitutive heterochromatin/euchromatin boundaries that in many current-day eukaryotes are important for local transcriptional silencing or readout (see Yashuhara and Wakimoto 2008, and references therein.)

It is important to note that I am *not* suggesting that the pachytene checkpoint is completely effective at detecting unmatched homologs and eradicating meiocytes carrying rearranged chromosomes. Instead, the data indicate that this checkpoint filter merely acts to increase the probability that gametes with the unaltered parental genome organization will produce the next generation. The synaptonemal complex, both in creating the pachytene checkpoint filter, and in fostering allele-shuffling recombination, merely opens windows of opportunity that give viable genomes a chance to pass into the future.

### The pachytene checkpoint in unicellular organisms: to be, or not to be, that is the question.

In a unicellular organism, pachytene checkpoint-induced apoptosis would seem to be a hard trait to faithfully pass on, and of questionable value. The yeasts, *S. cerevisiae* and *S. pombe*, were the first model organisms studied for cell cycle regulation. They belong to the enormously diverse, one-billion-year-old clade of fungi, represented today by between 2.2 and 3.8 million species (Hawksworth and Lücking 2017). These two yeast species have been diverging from one another for 320 to 420 million years. Both have stream-lined genomes with short and relatively few introns, likely adaptions for economical and rapid proliferation (0.1 and 1.0 introns per 1000 kb in *S. cerevisiae* and *S. pombe*, respectively (Csuros et al. 2011). However, analysis indicates that the first fungal ancestor had around 4.7 introns per 1000 bp, and that random, lineage-specific intron *loss* has shaped the various fungal genomes (Csuros et al. 2011). If the pachytene checkpoint arose during eukaryogenesis as a device to filter out gamete-producing cells with those slapdash break repairs that wreck TU continuity, then, given their intron-dense past, fungi should have inherited that checkpoint. In the many fungi with fruiting heads that produce abundant spores this may well be the case (although I know of no study of this issue). However, a filter to cull defective gamete-producing cells in favor of those with unadulterated genomes can only make sense when there are multiple meiocytes to select amongst. A mated unicellular *S. cerevisiae* or *S. pombe* cell triggered to sporulate is *de facto* both the parent and the one and only gamete-producing cell. For a unicellular organism to commit suicide to avoid passing on a flawed genome might improve its species’ pedigree, but a proclivity to suicide seems like a trait more easily selected against and lost, than selected for. So, what does the pachytene checkpoint do in yeast cells?

In *S. cerevisiae* a synaptonemal complex does form in sporulating cells (Roeder and Bailis 2000). Mutant cells that cannot synapse during meiosis I, or that have initiated but cannot complete recombination, enter a long-lasting pachytene arrest, but remain viable (Mitra and Roeder 2007). Certain mutations in the Pch2 gene allow cells that have been returned to a suitably nutritious medium to dissolve synapsis and resume *mitotic* cell division (Zenvirth et al. 1997). This second run at life is possible because Spo11-induced double-strand breaks are eventually repaired by recombination between sister chromatids (Zenvirth et al. 1997), and because in this budding yeast the pachytene checkpoint, although it can induce arrest, does not trigger apoptosis (Roeder and Bailis 2000).

In *S. pombe*, homologs do not synapse, no synaptonemal complex forms, and there is no Pch2 homolog (Wu and Burgess 2006). During meiotic prophase, this fission yeast builds instead “linear elements”, which are interpreted to be degenerate synaptonemal structures. These structures contain a subset of the synaptonemal complex proteins, including a meiotic HORMAD (Hop1), and *S. pombe* performs a subset of the meiotic functions that *S. cerevisiae* performs. This includes regulated recombination and mismatch repair (Roeder and Bailis 2000; Lorenz et al. 2004). Crosses between cells with reciprocal translocations and relative inversions are sterile, as would be expected if *S. pombe* did have a pachytene checkpoint. However, this appears to be due to meiotic drive genes and a failure of recombinational repair (Zanders et al. 2014). Ionizing-radiation of sporulating *S. pombe*, instead of triggering meiotic arrest of the gamete-producing mother cell, as occurs in *S. cerevisiae*, produces gametes that die of irreparable breaks, aneuploidy and chromosome fragmentation (Illner and Scherthan 2013). For the fission yeast, death seems to come directly from the slings and arrows of outrageous fortune, rather than by letting the pachytene checkpoint take arms against that sea of troubles, and by opposing, end them.

### Meiosis, the errant Y, and the plight of the single chromosome

Primary spermatocytes, although they come into being by a different developmental pathway, have all the same break-repair capabilities and use the same checkpoints that primary oocytes use (Lane and Kauppi 2019). However, in animals where sex determination is controlled by differentiated sex chromosomes, heterogametic individuals (e.g., XY males in mammals and flies; WZ females in birds, butterflies and moths) must contend with a meiotic difficulty that the homogametic sex does not face. For example, in the primary oocytes of mammalian females, the two X chromosomes are a homologous pair and can synapse during meiosis. By contrast, in mammalian males, each Y chromosome, which carries genes specific to male development, cohabits the primary spermatocyte with an X chromosome companion with whom it shares only a small region of homology (Handel 2004). Recombination occurs between these short regions of homology; this locks XY pairs together by a chiasma, and orients X and Y chromosomes towards opposite spindle poles, allowing the two sex chromosomes to segregate to different cells at the end of meiosis I. However, the majority of the Y chromosome's DNA shares no homology with the X and thus the pachytene checkpoint cannot monitor Y chromosomes for reorganizational mistakes. If the XY pair were subjected to the same strictures as other homolog pairs, incomplete synapsis would trip the pachytene checkpoint and condemn *every* primary spermatocyte to apoptotic death. Instead, the incompletely paired XYs are shielded from pachytene checkpoint surveillance by a process that involves histone modification, transcriptional silencing, and compaction of the heterogametic sex chromosome pair (Turner et al. 2006; Turner 2007; Checchi and Engebrecht 2011; Hirota et al. 2018).

The phenomenon known as Haldane's Rule likely results from interspecies incompatibilities that arise in creating the above shielding mechanism. Haldane's Rule stipulates that when in the hybrid offspring of a cross between individuals of closely related species, one sex is sterile, that sex will be the heterogametic sex (Haldane 1922; Dobzhansky 1936). Haldane's Rule pertains whether the heterogametic sex is the male or the female. This single-sex sterility highlights the existence of two distinct mechanisms for suppressing the pachytene checkpoint: full homolog synapsis or the histone modifications that shield heterogametic sex chromosome pairs. In crosses exhibiting Haldane's rule, it is the shielding mechanism that has presumably broken down, allowing the pachytene checkpoint to block gamete formation in the heterogametic sex.

Yet even when XY shielding from the pachytene checkpoint is fully operational, the incomplete XY homology has genetic consequences. This is evident, for example, by comparing the genomes of *Homo sapiens* with those of chimpanzees. Sequence comparisons reveal that since that time 6 million years ago when these species began diverging, their Y chromosomes have been diverging 50 times faster than their other chromosomes (Hughes et al. 2010). Genomic studies have revealed how sex chromosomes form *de novo* and change over time (reviewed in Graves 2006): the two sex chromosomes begin as homologs, with one member acquiring a sex-determining gene (e.g., the SRY gene in the male of placental mammals). Gradually other sex-advantage alleles accumulate on the same chromosome, due to the adaptive benefits of segregating together. What is notable is that, as the former homologs diverge and lose their ability to synapse, the heteromorphic chromosome undergoes progressive and rapid degeneration. For example, the Y chromosomes of both *H. sapiens* and the chimpanzee have acquired deletions, chromosomal rearrangements, inserted stretches of meaningless direct and inverted repeat sequence, and have suffered gene loss and transcriptional silencing by heterochromatinization. That the Y undergoes this dramatic genetic change has been blamed on Muller's Ratchet and the absence of XY recombination (e.g., Rice 1996; Charlesworth and Charlesworth 2000; Hough et al. 2014). This explanation seems inadequate, since no recombination should just lead to an accumulation of sublethal deleterious mutations. I suggest that the large-scale chromosomal rearrangements seen in the Y are instead the inevitable consequence of the Y chromosome's exclusion from a once-per-generation surveillance by the meiotic pachytene checkpoint. Without a proper homolog to serve as a standard of comparison during pachytene, inversions, deletions, and translocations arising in the Y from break-repair errors cannot be detected and the meiocytes with such defects cannot be culled out. Instead, the defects are passed on and rapidly accumulate. The same pattern of Y chromosome deterioration is seen in other species where male sex is determined by an unpaired Y chromosome (Checchi and Engebrecht 2011)

As already explained, chromosomal rearrangements tend to destroy TUs, so it is not surprising that whereas the primate X contains about 1000 TUs, the primate Y has lost all but 45 unique protein-encoding TUs (reviewed in Graves 2006). Heterochromatinization of the Y may be a protective adaptation to give dead genes a fitting burial and prevent them from being transcribed to no good purpose during mitotic cell cycles.

But what about the X? While residing in a spermatocyte and physically paired with a Y, anomalies arising in X chromosomes cannot be detected and culled out either. However, in a subsequent generation the X chromosome (or strictly-speaking its descendants) will be recycled through a homogametic individual. Any laxity the X may have enjoyed while passing a generation paired up with the undisciplined Y can be detected via synapsis with another X and dealt with appropriately then—perhaps one reason why during human fetal development two thirds of primary oocytes are culled (Hunter 2017). Analogous issues apply to the W and Z chromosomes in birds, moths, and butterflies (where ZZ is male and WZ is female).

In brachycerous Diptera such as *Drosophila*, sex is determined not by a heteromorphic sex chromosome, but by males having only one X chromosome and females having two. In *Drosophila* this is combined with the peculiarity of there being no crossing over during male meiosis, which means the pachytene checkpoint can only exist in female *Drosophila*. This fails to challenge my proposal that the pachytene checkpoint is necessary to head off chromosomal degradation, because *all* the chromosomes in male dipterans (including the X) are continually being circulated through females, where crossing over and a pachytene checkpoint arrest of abnormal chromosomes can operate. The absence of pachytene surveillance in males is thus inconsequential. It is possible too that suppressing synaptonemal complex formation in male *Drosophila* evolved as another way to prevent an unpaired X chromosome from triggering arrest in male gamete-producing cells.

Sex chromosomes are not only found in animals. *Ectocarpus* is a genus of brown marine algae with haploid and diploid phases in its life cycle, and with two sexes during the haploid phase (Coelho et al. 2019). In *Ectocarpus*, the V and U sex chromosomes determine the male or female identity of the haploid organisms (and their gametes). Like sex chromosomes in general, these two contain both pairing regions and sex-specific regions. During meiosis, the meiocytes contain both a female U and a male V chromosome, whose sex-determining regions are incapable of synapsis. Compared to the *Ectocarpus* autosomes, the sex chromosomes are found to contain higher levels of transposable elements, a lower gene density, and to exhibit signs of accelerated evolution (Luthringer et al. 2015). The *average* recombination rates between the U and V are *not* different than the recombination rates between autosomal homologs (Luthringer et al. 2015), providing direct evidence that reduced recombination is not the explanation for sex chromosome degradation. But sex chromosome deterioration *is* expected if UV chromosome pairs, in a manner analogous to XY chromosome pairs, are excluded from pachytene checkpoint surveillance.

In summary, the key ideas of the preceding several sections are these: (1) an unavoidable level of DNA break mis-repair will produce chromosome rearrangements; (2) the pachytene checkpoint acts to eliminate meiocytes with unmatched homologs, which selectively disposes of gamete-producing cells with reorganized chromosomes; (3) this process has the beneficial consequence of eliminating those gametes that have lost functional TUs; and (4) heteromorphic sex chromosomes are excluded from this surveillance process and therefore evolve much faster than autosomes, often losing genes other than those essential for sex determination and without which survival would be impossible. I suggest that the fate of heteromorphic sex chromosomes provides a glimpse of the future that all chromosomes would face without sexual reproduction and the pachytene checkpoint.

### Sexual reproduction is a conglomeration of genome-preserving functions

Every type of organism is engaged in a relay race across time, and the continuance of its particular life form depends critically on handing off to the next generation a genome that largely reproduces the parental phenotype. By making incremental adaptive changes, over the course of about two billion years, the eukaryotes have evolved to occupy virtually every habitat on this planet and have explored a vast miscellany of different life styles: (1) unicellular and multicellular; (2) haploid-dominant, diploid-dominant, and haplodiplontic (i.e., with haploid and diploid multicellular stages); (3) reproduction that is usually asexual, reproduction that is usually sexual, and reproduction that alternates between sexual and asexual; (4) transient expression of mating-types and fixed sexual assignments; and (5) compulsory outcrossing and compulsory self-fertilization.

**Fig. 9:**
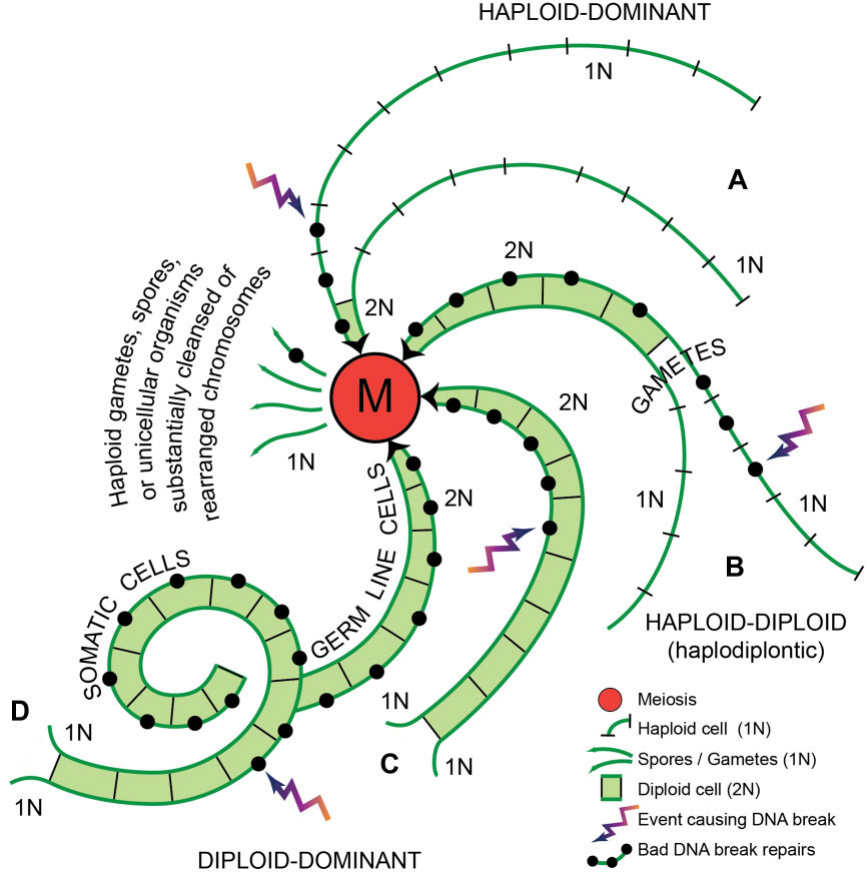
Diagrammatic representation of the most common eukaryotic life cycles. Haploidy is denoted by single lines, diploidy by double lines with green fill, the lightning bolt symbolizes events that cause double-strand DNA breaks, and the black dots signify a break mis-repair that has produced a chromosomal rearrangement. The chromosomal rearrangements are transmitted unchanged through successive mitotic divisions. During meiosis (red circle) the pachytene checkpoint filter reduces the likelihood that chromosomal rearrangements are transmitted to the haploid cells (green arrows), which meiosis produces. Depending on the species, these partially “cleansed” haploid cells can be spores, gametes, or a new haploid organism. Appendix I describes in more detail the eukaryotes whose life histories are here represented.

Fig. 9 depicts the life histories of the most common extant sexually-reproducing eukaryotes, emphasizing how ploidy transitions occur at different positions in different clades. 9A depicts the life cycle of the many morphologically-simple eukaryotes whose principal body form is haploid, 9B of the haplodiplontic plants and macroalgae, which mix multicellular haploid and diploid phases, 9C of the unicellular ciliates and diatoms, which curiously lack a synaptonemal complex, and 9D of the multicellular animals, whose somatic tissues are usually diploid. The separation of somatic and germline lineages, depicted in 9D, does not occur in all animals. Where it does, it allows germline cells to undertake measures to minimize DNA damage (e.g., suppression of cotemporaneous replication and transcription, locating male genitalia outside the body in warm-blooded animals, etc.). Interestingly, in the unicellular ciliates, a differentiation of germline and somatic *nuclei* occurs, to similar effect.

As we have seen, the maintenance of genetic continuity through time is threatened by two disparate types of entropic information loss: changes in the base sequence of a genome's encoded information, and double-strand breaks in the DNA double helix that were incorrectly repaired. The process that we call “sexual reproduction” allows eukaryotes to wage a defensive war against these vulnerabilities. During sexual reproduction, damaged TUs, faulty repair that has rearranged chromosomes, and ploidy are all managed for the good of the next generation. The different problems that sex ministers to, and the eukaryotic solutions to them, can be parsed out roughly as follows:

First, during the course of an individual lifetime, TUs are inevitably lost to mis-repair of random DNA breaks. Diploidy can increase the longevity of an individual organism by masking this loss with a good copy of the same TU. Because a simple redundancy of genes is sufficient to protect *somatic* cells from succumbing to random TU destruction, outcrossing, and meiosis need not be involved.

Second, large-scale chromosomal rearrangements and deletions are caused by the three, previously discussed, low-fidelity end-joining repair pathways that can erroneously connect together the wrong broken ends. Detecting these requires comparing homologous chromosomes obtained from two different individuals. Chromosomal rearrangements can then be filtered out of the germline by the pachytene checkpoint, and this requires meiosis, diploidy, and hence mating at some prior point in time. The filtering does not, however, require outcrossing, and the synaptonemal complex is notably indifferent to the relatedness of the two individuals whose homologs it strives to synapse. The consequences of this checkpoint fall equally upon the offspring of inbred and outcrossed unions, and as we shall see, upon the hybrid offspring of interspecies crosses too.

Third, masking *inherited* deleterious mutations to ensure the health of their future offspring requires, not merely diploidy, but also outcrossing. Otherwise, matings between closely related individuals risk producing offspring that carry two copies of the same deficient genes. The outcrossing requirement can add enormously to the cost and complication of compatible mate procurement. Depending on the species, outcrossing may require sexually dimorphic individuals, a sometimes-elaborate courtship, and—in the case of flowering plants—even recruiting the assistance of other species to serve as flying penises. Historically, much of the debate re the cost of sex (also known as the cost of males) relates to the need to ensure outcrossing.

Fourth, individual alleles can be corrupted by base-changing mutations created by chemical damage, by nonhomologous end-joining having added or deleted a small number of bases in preparing DNA ends for re-ligation, and by base pair mismatches accidentally produced during DNA replication or excision repair of the double helix. As has long been understood, recombination during meiosis lets eukaryotes expose their alleles in new combinations to purifying (and adaptive) selection. The opportunity to remove deleterious alleles from a gene pool instead of merely sweeping them under the carpet of diploidy requires recombination during meiosis, and therefore prior outcrossing. The possibility of escaping Muller's Ratchet first requires that recombination move defective alleles from the homolog provided by one parent to the homolog provided by the other; it then requires that a random segregation first of homologs, and then of sister chromatids, allows some lucky gametes to emerge from meiosis burdened by fewer, or at least different, deleterious alleles.

As illustrated in Fig. 9—in furtherance of their progeny's survival—different organisms schedule meiosis at different times in their life cycle. In haploid-dominant organisms, cell fusion immediately *precedes* meiosis. In these species, meiosis is often brought on by the very circumstances for which it provides a remedy. For instance, starvation in unicellular algae and fungi is often what triggers meiosis and the production of spores, which can disperse to potentially more favorable environments. In several pathogenic haploid protozoans, it is exposure to the DNA break-causing oxidative defense systems of their host, that triggers the haploid pathogen to mate (Bernstein et al. 2018). In diploid-dominants, fertilization immediately *follows* meiosis, creating the diploidy which can extend each offspring's lifespan. Here, diploidy helps ensure that complex multicellular animals can attain reproductive age, even in the face of an inherited burden of deleterious mutations and an ongoing random loss of TUs because of faulty repair of double-strand breaks.

In Appendix I, I describe in more detail the life histories of the different sexual life forms, emphasizing their somewhat different ways of splicing together the eukaryotic toolkit for coping with random base changes, DNA breaks, and the mis-repair thereof. Appendix II provides an overview of the most common modes of *asexual* reproduction—natural experiments which reveal the short and long-term consequences of not having the full complement of genome-protecting measures that sexual reproduction provides; it also illustrates some of the inventive workarounds produced by natural selection, and their limitations.

The benefits described above can explain why losing sexual reproduction would lead to early extinctions. These benefits, plus the consequences of the pachytene checkpoint for speciation (presented below) would seem to provide sufficient explanation for the prevalence and persistence of sexual reproduction in the Eukarya.

### Does the pachytene checkpoint maintain discrete species?

The most generally agreed upon definition of a species is that provided by Ernst Mayr: “species are groups of actually or potentially interbreeding natural populations which are reproductively isolated from other such groups” (Mayr 1942). Some between-species interbreeding does succeed, even in the wild. Yet for the most part, low levels of genetic mixing keep each species’ genome distinct, functionally cohesive, and well-adapted to survive in its own particular habitat. Charles Darwin was greatly perplexed as to how the process of natural selection he envisioned could account for speciation. Indeed, he worried that the very existence of discrete species revealed a flaw in his theory: “Why, if species have descended from other species by insensibly fine gradations, do we not everywhere see innumerable transitional forms? Why is not all nature in confusion instead of the species being, as we see them, well defined?” (chapter 6; Darwin 1859).

Long before the pachytene checkpoint was discovered, the cytogeneticist M.J.D. White had argued that alternate chromosomal layouts, such as inversions and translocations, must somehow be important for the existence of separate species (White 1978). His extensive surveys of fruit flies and grasshoppers consistently found that within those groups, each species was characterized by a unique chromosomal organization, distinguishing it from even its closest relatives. Modern sequence analyses comparing, for example, genomes in chimpanzee *vs.* human, or insect species that occupy overlapping and contiguous habitats (e.g., mosquitos in Africa and fruit flies in the Americas), show the same thing: multiple chromosome inversions and translocations differentiate sibling species (Ayala and Coluzzi, 2005). Moreover, analysis of gamete formation in several sterile hybrids derived from matings between sibling species revealed that germline cell death was occurring in meiosis I, either during or soon after the pachytene stage, suggesting the involvement of the pachytene checkpoint (Li et al. 2009).

Crosses between two species of yeast with a pachytene checkpoint, *Saccharomyces mikatae* and *S. cerevisiae*, provide support for the idea that this checkpoint can cause hybrid sterility. These two yeasts have reciprocal translocations involving three chromosomes, and their hybrids are almost entirely sterile. Notably, when Delneri et al. reengineered the *S. cerevisiae* chromosomes to make them collinear with those of *S. mikatae*, hybrid fertility was significantly restored (Delneri et al. 2003). It is also noteworthy that two other yeasts, *S. cerevisiae and S. paradoxus*, whose genomes have diverged by about 12% and whose hybrids are normally sterile, can be made to produce offspring at about the same rate as non-hybrid crosses by silencing two mismatch repair genes (SGS1 and MSH2) specifically during meiosis, which causes synapsis and recombination to be blocked (Bozdag et al. 2021). From these findings, I conclude that without synapsis, there is no ability to detect mismatched homologs, no pachytene checkpoint, and consequently no ability to create hybrid sterility.

Li et al (2009) were, to my knowledge, the first to lay out the case for the pachytene checkpoint being the cause of sterility in hybrid offspring when individuals with differently organized homologous chromosomes mate. In the first half of this essay, I reviewed evidence that DNA double-strand breaks are common and are the most pernicious destroyer of eukaryotic genomes, so that all eukaryotic cells are constantly involved in DNA break repair. I further argued that the adaptive function of the pachytene checkpoint is to reduce the likelihood of transmitting to the next generation genomes that have lost functional TUs due to accidental break mis-repair. That checkpoint executes its function by culling out gamete-forming cells that contain chromosomal rearrangements, based on whether or not the synaptonemal complex is able to fully synapse a gamete-producing cell's homologs. Li et al. review data showing that the pachytene checkpoint is not equally effective in all species, or even in both sexes of the same species (Li et al. 2009). Regardless, the global consequence of this checkpoint is to increase the odds that matings between individuals of the same species will be those most likely to leave viable descendants. Thus, what has long perplexed Darwinian scholars—how fertility and sterility *could both be adaptive* in the same population—is explicable as an unavoidable side effect of accidental chromosomal reorganization caused by double-strand break repair mistakes, and of how the pachytene checkpoint detects and eliminates gene-destroying mis-repair in meiotic cells.

Closely related species typically differ by multiple chromosomal rearrangements; inversions both large and small are especially common. For example, a comparison of primates reveals that humans have 6 unique large inversions with respect to other primates, chimpanzees have 7 unique to their species, gorillas 6, orangutans 3, and macaques 17, ranging in size from 103 thousand to 91 million bp (Catacchio et al. 2018). Within each species, both homologs carry the same fixed chromosomal layout; analysis of various taxon groupings show that inversions can remain constant for hundreds of thousands, or even millions, of generations (Wellenreuther and Bernatchez 2018).

The pachytene checkpoint, by comparing homologs and eliminating meiocytes with unmatched chromosome pairs, will—during repeated rounds of outcrossing and meiosis—homogenize chromosome structure in a community of interbreeding individuals. But if two subpopulations have attained some amount of divergence in their chromosome structure, this same mechanism will reduce the ability of members of the two subpopulations to pass on intermingled genomes, even if they do inter-breed. Because of this barrier to gene exchange, nascent species, differentiated just by chromosome organization, can begin evolving apart. Thus, the pachytene checkpoint helps to explain what had puzzled Darwin so greatly—why species diverge and are well defined.

Without the pachytene checkpoint constantly plucking out the meiocytes of hybrids, Darwin's fine gradations of intermediates might indeed occur. It is therefore noteworthy that diatoms, which lack key proteins needed to construct the synaptonemal complex (Patil et al. 2015; Hofstatter and Lahr 2019), do exist in what Darwin might well have called “innumerable transitional forms”. Diatoms reproduce sexually, and they have morphologically and genetically distinct species set apart by geographical and habitat adaptations, mate preferences, and various prezygotic reproduction barriers. Nevertheless, an almost bacteria-like hybridization has occurred, such that in under 250 million years an estimated 30 to 100 thousand diatom species and crypto-species have formed; there is such a subtle continuum of morphological features that classification is virtually impossible (Cooper and Masly 2013; Mann and Vanormelingen 2013).

### Can the pachytene checkpoint help to create new species?

As often noted, notwithstanding the title of his great book, Charles Darwin did not explain how new species originate. What he explained instead was how natural selection could shape the inherited traits of extant species, potentially allowing species to gradually diverge further and further from one another.

Alfred Russel Wallace, the co-originator of the theory of evolution, thought that the ability of interspecies crosses to produce only infertile hybrid offspring must somehow be key to speciation. However, since ultimately natural selection rewards an individual's reproductive success, it was a puzzle to those first proponents of evolution by natural selection how something as seemingly maladaptive as hybrid sterility could be selected for. As the previous sections explain, I believe this paradox can be resolved by understanding the critical importance of the pachytene checkpoint for gene heritability—and the idea that this checkpoint creates hybrid sterility as a side effect. However, the classical explanation, arrived at separately by William Bateson, Theodosius Dobzhansky, and Hermann Joseph Muller, proposes a different explanation (for historical reviews see Orr 1996; Pinho and Hey 2010).

The Bateson/Dobzhansky/Muller incompatibilities model stipulates that for one species to give rise to two, subpopulations must be reproductively separated while random mutational change brings about genetic divergence between them. Once two or more factors (produced by two or more alleles) have lost their ability to function compatibly in combination due to this divergence, matings between members of those two subpopulations will produce inviable or sterile offspring. Thenceforth these subpopulations, whether still sequestered or reunited, will constitute reproductively-isolated species, incapable of creating viable hybrid offspring.

Notwithstanding this well-established doctrine, there are circumstances, such as the speciation of fish within the same lake, or of highly mobile birds and winged insects occupying adjacent habitats, or the existence of cryptic species within large continuous plant communities, where it has been hard to believe that such a separation ever occurred. This raises the following question: *could the pachytene checkpoint, reacting to chromosomal rearrangements caused by end-joining DNA break repair mistakes, upon occasion create a reproductive barrier sufficient to initiate speciation from within a population in the absence of geographical or habitat partitioning?*

**Fig. 10:**
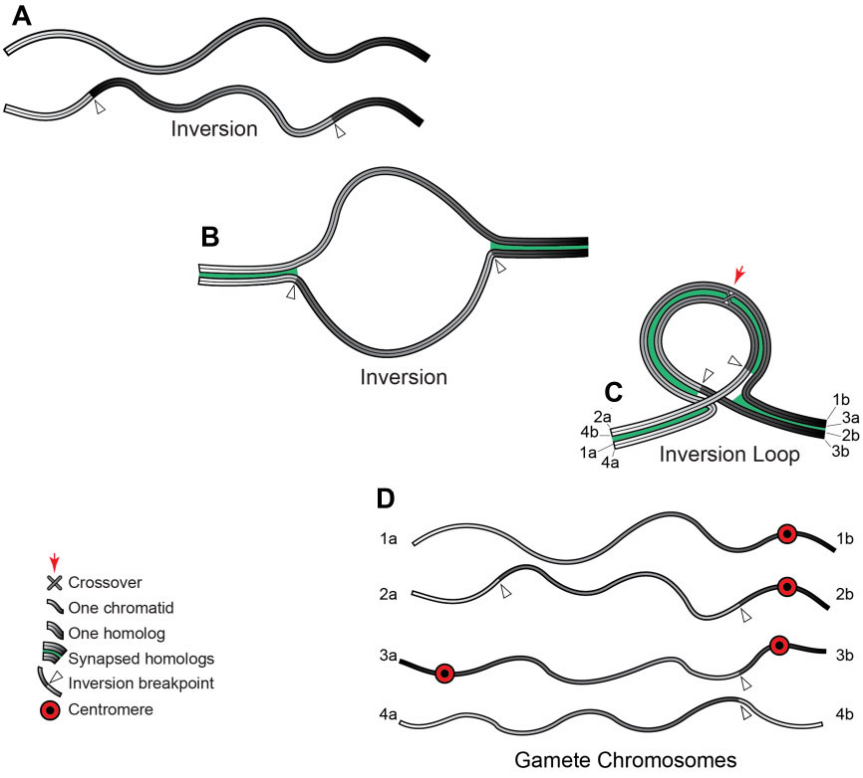
Diagram shows how inversions appear to suppress recombination during meiosis. White-to-black shading indicates position along each chromosome and arrowheads mark inversion end points. Numbers in C mark chromatid ends, to facilitate following their participation (or not) in crossing over. If only one member of a homolog pair carries an inversion (A), synapsis may fail outright (B), or synapsis may occur via an inversion loop (C). Crossing over within an inversion loop during meiosis I (indicated by red arrow in C), produces at the end of meiosis II one non-inverted chromatid (D1a–b), one chromatid with the original inversion (D2a–b), and two recombinant chromatids (D3a–b and D4a–b). If the inversion is paracentric (the centromere is outside the inversion), the recombinant chromatids will have either two or zero centromeres, plus gene duplications or deletions (D3a–b and D4a–b), as diagrammed. If the inversion is pericentric (centromere within the inversion), the recombinant chromatids will carry just duplications and deletions (not shown). In either case, crossing over within an inversion produces chromatids with faulty sets of chromosomes, leading on to nonviable aneuploid zygotes. Thus, alleles located within inversions can only pass from generation to generation via unrecombined chromatids (D1a–b and D2a–b).

As regards that possibility, I believe that this century's most significant discovery was that the traits that distinguish sibling species, and those associated with polymorphisms in an interbreeding population, often map to inversions (see reviews in Wellenreuther and Bernatchez 2018; Fuller et al. 2019; Huang and Rieseberg 2020). In inversion heterozygotes, meiotic crossing-over between the inverted and the non-inverted region of homologous chromatids produces duplications and deletions (and in the case of paracentric inversions, dicentric, and acentric chromatids as well). Fig. 10 illustrates this diagrammatically. As a consequence, only the non-recombining chromatids in a homolog pair can contribute to the production of viable offspring, so that inversions have the effect of suppressing recombination (Sturtevant and Beadle 1936). If traits with survival importance are encoded by alleles grouped within an inversion, those alleles will not be reshuffled during meiosis, but will remain together and be faithfully passed on as a unit, generation after generation.

The yellow monkeyflower *Mimulus guttatus* provides a particularly clear example of traits with adaptive significance being held within an inversion. Here flowering times and annual-to-perennial life-history shifts (and as a consequence, the ability to occupy two quite distinct habitats) are associated with alternative inversion polymorphisms (Lowry and Willis, 2010). Similarly, what was initially classified as a single species of malaria-carrying African mosquito—*Anopheles gambiae*—has since been shown to be a complex of species, differentiated from one another by inversions (Coluzzi et al. 2002; Cohuet et al. 2004; Tripet et al., 2005); six sibling mosquito species of virtually identical morphology differ in breeding site preferences, blood meal hosts, and tolerance of arid conditions, and each species has a distinctive chromosomal configuration (Ayala and Coluzzi, 2005).

Other examples abound. In the fruit fly, *D. pseudoobscura*, the relative frequencies of certain inversions carried on the third chromosome exist in an east-west cline across the southwestern United States; these frequencies have remained stable since at least the 1940s when they were first described, even as markers on other chromosomes segregate freely (for references, see Schaeffer 2008). Certain other inversion combinations in *D. pseudoobscura* exhibit seasonal cycling (Dobzhansky 1948). *D. subobscura* carries numerous inversions whose frequencies form latitudinal clines across a broad territory in Europe. After an accidental introduction of this fly into North and South America, a subset of these inversions rapidly established themselves along similar latitudinal gradients, implying that they too enclose traits under strong adaptive selection (Balanyà et al. 2003). Following on those discoveries, a whole variety of phenotypic traits in other organisms—affecting habitat preference, mimetic coloration, social behavior, migratory behavior, and sexual preference—have been found to map within inversions (e.g., Noor et al. 2001; Brown et al. 2004; Faria and Navarro 2010; Wellenreuther and Bernatchez 2018; Fuller et al. 2019; Huang and Rieseberg 2020).

Assuming that the pachytene checkpoint reduces the fertility of organisms carrying differently-organized homologs—say if one homolog carries an inversion that the other homolog lacks—could this pre-condition result in the formation of a new species, even without geographical separation? In broad brush strokes, the following is a scenario for how speciation might begin, without contravening the principle of adaptive evolution by natural selection.

We know that the between-homolog allele shuffling that meiosis generates will, by chance, occasionally assemble a group of alleles that confers a local fitness advantage. Because allele reshuffling normally occurs at every meiosis, such fortuitous groupings are usually short-lasting. But suppose that a pair of DNA breaks on one homolog is rejoined incorrectly, creating an inversion that locks this propitious suite of alleles together, thus preventing their reshuffling during meiosis. If this occurs without damaging the TUs at the breakpoints, (e.g., as was revealed by DNA sequencing to be the case for six *D. pseudoobscura* inversions; Fuller et al. 2017), no encoded information has been lost and gene expression will be unaffected. The gene order of an internal segment of a chromosome has simply been flipped (Fig. 10A). This newly flipped segment poses no problem during mitotic cell cycles, and during somatic and germline cell expansion the cells function and replicate normally.

However, upon reaching meiosis the newly inverted region will find itself unable to align normally with its homolog (10B). Primary meiocytes carrying one inverted homolog may trip the pachytene checkpoint, bringing down upon themselves arrest or death by apoptosis, and thereby curtail the prospects of this promising assemblage of alleles. But, unless the pachytene checkpoint is 100% efficient, some gametes carrying the inversion will be created. When one of these contributes to a zygote, the inversion can traverse another entire life cycle. Although it will face the same precarious fate at the next meiosis, the beneficial allele assembly within the inversion has avoided meiotic reshuffling, has been carried forward through time, and is now present on one chromosome in every cell, including in *every* germline cell, of at least one organism.

To the extent that the alleles within an inversion improve an organism's ability to prosper in its local environment, it will be selected for. Acting contrariwise, the pachytene checkpoint will reduce the *quantity* of gametes produced by individuals that are inversion heterozygotes (as compared to individuals carrying exclusively collinear homolog pairs). So, to persist in the long run, the phenotypic benefit conferred by the alleles within the inversion must offset that fecundity handicap long enough for homologs carrying identical inversions to be paired in a zygote, perhaps due to matings between offspring of the same lineage. Each such union will create individuals homozygous for the inversion. An accumulation of these inversion homozygotes constitutes a prospective neo-species, capable of mating *inter se* with no risk of tripping the pachytene checkpoint, since their homologs are now all collinear with respect to one another. From this point forward, this genome competes with the parental genome without any reproductive disadvantage imposed by the pachytene checkpoint. Instead, it is the reproductive success of the *hybrid* offspring conceived by matings between each inversion-carrying organism and its parental species that will be disadvantaged by defective homolog synapsis. Furthermore, the potential neo-species has established a genetic beachhead for the entire stretch of genes previously located within the inversion, which now exists as a length of genetic homozygosity on a collinear pair of neo-species’ homologs. Thus, might a first step towards the formation of a new species be taken.

In the above discussion it is important to distinguish between the effects in inversion heterozygotes that inversions have in preventing recombination within each inverted stretch of chromosome, and the culling by the pachytene checkpoint of gamete-producing meiocytes carrying relative inversions, which reduces the total number of gametes produced. Careful egg counts have measured the effect of inversions on the *viability* of different crossover classes in the eggs that have been laid (e.g., Sturtevant and Beadle 1936). However, I know of no quantitative measurements of the effect that inversions and other types of chromosomal rearrangement have on the total quantities of eggs produced. It is the latter numbers that are needed to model the role the pachytene checkpoint plays in speciation.

### How a pachytene checkpoint model for speciation compares to other models

Kirkpatrick and Barton have proposed that when inversions have captured alleles that confer a fitness advantage for local conditions, inversions will be selected for on the basis of that advantage alone, without any need for geographical isolation (Kirkpatrick and Barton 2006). The pachytene checkpoint pathway to speciation, sketched out above and described in greater detail below, begins with that same supposition. Those authors show by mathematical modeling that, in the absence of a countervailing force, an inversion with its captured adaptive alleles will be driven to high frequency (Kirkpatrick and Barton 2006). They propose that it is by capturing both adaptive and deleterious alleles, that an inversion may be stabilized at a low or intermediate frequency. This, they propose, could explain the many polymorphic populations with inversions stably maintained at a wide range of different frequencies, or stably cycling in response to seasonal change. By contrast, in the pachytene checkpoint speciation model that I propose, it is not just the balance and potency of the alleles within an inversion, but that these, in combination with checkpoint culling, will create a robust push-pull mechanism that stabilizes each inversion at its own specific frequency.

My proposed model for speciation driven by the pachytene checkpoint differs from the classical Bateson/Dobzhansky/Muller allelic incompatibilities model in requiring no separation of the diverging species by geography or habitat. Neither genetic drift, nor a genetic bottleneck, nor a lengthy period of reproductive separation is needed while random, genome-wide mutations create genetic incompatibilities, as required by the Bateson/Dobzhansky/Muller allelic incompatibilities model. In pachytene checkpoint-driven speciation, the difference between the parent and the presumptive neo-species is *initially* confined just to allele(s) within relative inversion(s). The “genetic beachhead” described above will be reached only if the inversion-captured trait confers an advantage sufficient to establish itself in the face of pachytene checkpoint culling. But if inversion homozygosity is attained, homolog synapsis and recombination during meiosis will resume between the neo-species’ now collinear chromosomes, while the pachytene checkpoint will depress gene flow between the nascent neo-species and the parental species *for genes on all chromosomes*. This is because thenceforth every gamete that the neo-species contributes to a hybrid will carry one inverted chromosome which, due to its inability to synapse with its homolog, may trigger the pachytene checkpoint.

High resolution sequencing of related species has made it possible to distinguish chromosomal regions with different levels of allelic diversity and sibling species show higher genetic divergence in their relative inversions than in their collinear chromosomes (e.g., Kulathinal et al. 2009; Fuller et al. 2018). Other studies have shown that genes responsible for reproductive isolation—for example, causing gametic incompatibilities, zygote death, different flowering times, and mating preferences—often map to inversions, just as some adaptive traits have been found to do (Wellenreuther and Bernatchez 2018; Huang and Rieseberg 2020). In particular, the discovery within inversions of alleles responsible for hybrid sterility (e.g., Brown et al. 2004; Noor et al. 2001, 2007), has produced a permutation of the original Bateson/Dobzhansky/Muller allelic incompatibilities model, known as “speciation-with-gene-flow” (see Noor et al. 2007; Kelleher and Barbash 2010; Pinho and Hey 2010; Feder et al. 2012; Wang et al. 2020). This model goes as follows: when a single species is split into two separated subpopulations, random mutations across the entire genome of both subpopulations, will gradually begin to differentiate them one from the other. Following recontact, mutually incompatible alleles will be eliminated from the chromosomes that in the two populations are collinear. But because inversions prevent recombination, allelic incompatibilities can persist, and new ones may even arise, within relative inversions. The discovery of barriers to hybridization within inversions, plus sequence data compatible with recombination and the shedding of incompatible alleles outside of inversions, has led to the supposition that, while inversions are accumulating incompatibility alleles, interbreeding between the two future species must to some substantial degree be continuing—hence *speciation-with-gene-flow*. As I will explain below, the pachytene checkpoint model and a slightly different chronology should generate the same twin features, requires no period of subpopulation separation, and appears to better accord with evolutionary histories.

An altogether different model for speciation has been advanced by Jackson and Mistry (2020). They too propose that a meiotic checkpoint reacting to chromosome rearrangements drives speciation. In their model it is not the pachytene checkpoint, but the *spindle assembly checkpoint of meiosis II*, that is the primary driver of new species formation (Jackson and Mistry 2020). The spindle checkpoint helps prevent aneuploidy by arresting cells at metaphase until spindle microtubules have attached correctly to paired sister chromatids (Lara-Gonzalez et al. 2012). Jackson and Mistry argue that chromosomes that have undergone fusions or fissions, causing a change in overall chromosome number, will still pair during meiosis with the chromosomes from which they were derived, although many pairs will permanently trigger the spindle checkpoint, thereby producing gametes only at a reduced frequency. However, should a mutation occur in the chromosomal variant, one that is sufficiently beneficial to offset this fertility reduction, lineages carrying the beneficial mutant can pass through the bottleneck of reduced fertility to form two reproductively-isolated neo-species with different karyotypes. In this way, a new species can form without geographical isolation, much as proposed above for the pachytene checkpoint model.

Jackson and Mistry (2020) show with mathematical modeling that their proposition that chromosomal fusions and fissions drive speciation is feasible. It is notable though that whereas *Homo sapiens* is differentiated from the great apes by having one fewer chromosome, the result of a chromosomal fusion, no such difference in chromosome number distinguishes the various apes from one another (Müller and Wienberg 2001). By contrast, numerous species-specific inversions and translocations differentiate *all* of the various primate species (Müller and Wienberg 2001; Catacchio et al 2018). Likewise, detailed genetic analyses of the *D. pseudoobscura* and *D. melanogaster* genome sequences, which began diverging 25–55 million years ago, reveal no chromosomal fusions or fissions but very many changes in gene order in the same fixed set of chromosomes, inversions being especially common (Richards 2005). In *D. pseudoobscura* the rearrangement endpoints studied correlate with repeat sequences, as would be expected if those chromosomal rearrangements had originated from mistakes in break repair made by a direct annealing break repair pathway (Richards 2005). The inversions in these examples, and the many others that commonly differentiate sibling species, have the potential to trigger the pachytene checkpoint, but not the spindle assembly checkpoint. I therefore conclude that the former checkpoint is much more likely to be the primary meiotic driver of species formation.

### How the pachytene checkpoint can function as a ratchet, driving the fixation of sufficiently adaptive inversions and creating new species

The selective effect that the pachytene checkpoint has on fecundity, acting *in conjunction with adaptive selection*, may alter the genetic makeup of different lineages within a species, without requiring physical separation of the species’ subpopulations. I distinguish four conceptually-distinct phases in the progress towards the formation of a new species, all driven by the pachytene checkpoint, with each successive phase having a larger genetic footprint.

Phase 1: Inception. By culling meiocytes in which an inversion has formed, the pachytene checkpoint reduces the number of inversion-carrying gametes in the gamete population, so that within an interbreeding population most inversions will gradually be extinguished. Since recombination will continue external to the inversion, this lowers the frequency in a gene pool of all those *alleles* that happen to lie within an inversion, unless these alleles confer a benefit sufficient to increase the relative abundance of the individuals carrying the inversion.

Phase 2: Establishment. If the collection of alleles locked within an inversion provides a large enough survival or reproductive advantage, natural selection can act in opposition to the pachytene checkpoint, potentially increasing the prevalence of those individuals carrying the inversion. The multi-generational tug-of-war between inversion-captured fitness edge *vs.* checkpoint-induced meiocyte suppression then determines whether an inversion will be driven to fixation or extinction, and in populations with inversion polymorphisms, can set the frequency of the inversion-carrying chromosome(s). Note that in a freely-interbreeding population, collinear homologs will continue to recombine and segregate at random, even as the pachytene checkpoint continues to eliminate meiocytes that are inversion heterozygotes. Thus, the pachytene checkpoint will give the appearance of affecting just the frequency of the inversion-carrying *chromosome(s)*. Meanwhile, the inversion *per se* will continue to suppress gene flow into and out of the inversion, as already explained.

Phase 3: Fixation. A long-lasting Phase 2 will inevitably generate inversion homozygotes, which can interbreed without the checkpoint handicapping their fecundity, as already explained. From thence forward the pachytene checkpoint creates a (partial) barrier to gene exchange with the parental species, by reducing the fecundity of hybrids whose homologs differ in chromosomal organization. This begins to partition the population into two: the parental species and a neo-species in which the adaptive trait has been fixed by homozygosity. Importantly, in a population that is polymorphic with regard to a chromosomal inversion, the step to inversion homozygosity will occur repeatedly, gradually capturing for any future neo-species much of the parental species’ allelic diversity exterior to the inversion. Once enough inversion homozygotes exist to constitute a viable outbred reproductive population, the critical first step in the fixation of an adaptive trait by means of chromosomal inversion has taken place. During Phase 3 the checkpoint now partially secures the *entire genome* of the inversion-bearing neo-species from genetic mixing with the parental population. As a consequence, other locally-adaptive alleles, in addition to those captured by the original inversion, can be selected for, will be more reliably passed on, and will begin to accumulate, gradually further differentiating the two subpopulations that began as a single species.

Phase 4: Reinforcement. In organisms that must mate to reproduce, including flowering plants that use other species as male gamete carriers, there now arises a benefit to the formation of barriers that prevent the neo-species and the parental species from wasting reproductive effort by mating with one another. These supplemental barriers can include phenotypic and behavioral adaptations of the sort discussed in the final section of this essay. Supplemental reproductive barriers provide less benefit to non-mating species that free spawn into ocean waters, or to the grasses, conifers and flowering plants whose pollen is wind-dispersed, since they are unlikely to prevent gamete wastage. Yet, in both mating and non-mating organisms, the pachytene checkpoint does that thing that was thought to make geographic separation essential for speciation—it permits an accumulation of genome-wide Bateson/Dobzhansky/Muller allelic incompatibilities that will further differentiate two subpopulations, by impeding gene flow between them. Thus, even in sympatry, the pachytene checkpoint will drive an increasing reproductive isolation of emerging *species.*

Mathematical modeling could test and add important quantitative constraints to the above verbal hypotheses. Unfortunately, key real-world information—exactly how much the pachytene checkpoint reduces gamete production in inversion heterozygotes—is as yet lacking. Moreover, the strength of this checkpoint apparently varies between species and even between the two sexes in one species (Li et al. 2009). However, the evolutionary histories that I review next are more consistent with the above sequence of events than with speciation-with-gene-flow.

### Adaptive inversions precede speciation

Three well-studied examples suggest that polymorphic populations in which adaptive inversions have become established are a commonplace precursor to eventual speciation—with the potential for subpopulations carrying a subset, or all, of the adaptive inversions to progress on to full reproductive isolation.

The fruit fly, *Rhagoletis pomonella*, is in the process of adapting to exploit an introduced food source—apples—which became available to it only within the last 150 years. Apples begin ripening earlier than this fruit fly's traditional food, which in the northeastern United States is the fruit of the native hawthorn, and the existence of multiple apple varieties creates a very protracted fruiting season. *Rhagoletis* is accomplishing this adaptation by regulating when it emerges from winter diapause. Different alleles of six enzymes, whose frequencies correlate with the timing of adult eclosion, are trapped in inversions. Natural selection has produced a *Rhagoletis* complex that is polymorphic for these inversions, and for eclosion timing—creating a fruit fly population that can take advantage of an extended fruiting season that includes both their new and their original host plants (Feder et al. 2003). The *Rhagoletis* circumstance resembles what Schaeffer modeled with *Drosophila pseudoobscura* in mind. He demonstrated that in a population that is polymorphic for adaptive inversions, the frequencies of individual adaptive inversions can be stably maintained by selection in a heterogeneous environment (Schaeffer 2008). In the case of *Rhagoletis*, the range of apple ripening times is the heterogeneous environment, and what is being selected upon is eclosion timing (currently determined by genes captured within inversions). Furthermore, one can imagine how, in an apple variety monoculture, one inversion whose marginal fitness was greater than the mean fitness of the overall population might attain neo-species status by the mechanism described in the previous section.

Reconstruction of the evolutionary histories of chromosomal inversions in *D. persimilis* and *D. pseudoobscura*, using more complete sequence comparisons than previously, shows that, like *Rhagoletis* and contrary to widely accepted ideas (e.g., Kulathinal et al. 2009), these inversions existed as polymorphisms in a common ancestor before these sympatric sister species became reproductively isolated (Fuller et al. 2018). As a third example, analysis of the six species of mosquito in the *Anopheles gambiae* complex similarly indicates that an ancient inversion polymorphism predated the diversification of the entire complex into discrete species, which now have different habitat and food specializations (Fontaine et al. 2015).

### Sequence data are consistent with pachytene checkpoint-driven speciation

Beginning with a population carrying adaptive inversions, the pachytene checkpoint should produce the same DNA sequence patterns that speciation-with-gene-flow was thought necessary to explain, as described next.

During the Establishment phase of the checkpoint-driven speciation model (Phase 2), inversion heterozygotes mate at random and collinear homologs recombine freely. This, together with adaptive and purifying selection, aids in the assembly of genomes whose alleles work well together. Randomly-mutated alleles accumulate within inversions due to the recombination suppression therein, but these alleles are transcribed and expressed just as if they resided exterior to an inversion. Maladaptive alleles, *wherever located*, if sufficiently deleterious to cause the demise or reproductive failure of the organism that carries them, will be removed from the gene pool by purifying selection. On the other hand, during a long-lasting Phase 2, locally beneficial, neutral and deleterious-but-survivable alleles will continue to accumulate within adaptive inversions. As pointed out by Fuller and colleagues, inversions are the hotbed for nurturing allelic novelty (Fuller et al. 2018).

During the Fixation phase in the checkpoint-driven speciation model (Phase 3), in inversion *homozygotes*, any alleles detrimental to the survival of the neo-species, which previously had been sheltered within an inversion, will now gradually be eliminated by purifying selection (since recombination can now separate these from the adaptive alleles). On the other hand, because there is no selective advantage to eliminating those alleles that merely create reproductive incompatibilities with the parental species, these would be expected to remain. During a lengthy Phase 2, many such incompatibilities may have accumulated within what were then relative inversions, and persist on what become collinear homologs in Phases 3 and 4. Note also that in the pachytene checkpoint model, allelic divergence across the entirety of both nascent species’ genomes is expected to *follow* (during Phases 3 and 4), *rather than precede*, reproductive isolation. This is opposite to what is postulated in both the speciation-with-gene-flow model and the classical Bateson/Dobzhansky/Muller allelic incompatibilities model.

During the Reinforcement phase of the checkpoint-driven speciation model (Phase 4), allelic incompatibilities and, in species that must mate to reproduce, also “speciation genes” that reduce *mating* between members of sibling species, are expected to develop. This means that the original chromosomal reorganization is unlikely to remain the only impediment preventing sister species from successful hybridization. For example, the *Saccharomyces* yeasts consist of six species which readily hybridize and whose hybrids produce virtually no viable spores. Three of these species (*S. cerevisiae, S. mikatae*, and *S. paradoxus*) are distinguished by reciprocal translocations, implying that they likely originated by chromosomal reorganization. Engineering the *S. cerevisiae* genome to be collinear with *S. mikatae* partially restores hybrid fertility, but only partially (Delneri et al. 2003). This result implicates the pachytene checkpoint in providing part of the barrier that prevents these sibling yeast species from hybridizing, but shows that this reproductive barrier has been further reinforced. Genetic analysis of two sunflower species, *Helianthus petiolaris* and *H. annuus*, which grow together but hybridize only occasionally, reveals the same thing. Suppressed gene flow between collinear and rearranged chromosomes accounts for roughly half of the reproductive barrier between these two species, with the rest being due to incompatible alleles and speciation genes distributed across many chromosomes (Rieseberg et al., 1999; Rieseberg and Blackman, 2010).

It is notable that sister species that occupy overlapping or contiguous habitats consistently carry more numerous inversions than sister species that are geographically isolated (Noor et al. 2001; Brown et al. 2004; Castiglia, 2013; Hooper and Price 2017). For the latter, during Phase 4, there has been no selective advantage that would drive an accumulation of supplemental reproductive barriers. That the barriers that form to reproductively isolate contiguous sister species should involve inversions may be because inversions are the usual birthplace for new allelic diversity, and hence for new speciation genes, or because inversions *per se* depress hybrid formation due to the culling effect of the pachytene checkpoint, or both. The speciation genes analyzed to date encode proteins with multiple amino acid changes, suggestive of alleles protected from recombination within long-lasting relative inversions (see references in Fuller et al. 2018; and 2020).

To summarize, speciation-with-gene-flow is *not* required to explain how it came to be that the reproductive barriers separating sibling species are located within relative inversions, while at the same time regions outside of the inverted regions carry the genetic signatures of recombination and purifying selection. In checkpoint-driven speciation this duo of characteristics can arise sequentially. *Crucially, the pachytene checkpoint itself is the vehicle that tends to drive genomes that contain adaptive inversions towards speciation.* Perhaps this is why asexual species that have abandoned meiosis tend not to give rise to new species, but instead sit on the tips of unbranched twigs on the Tree of Life (Bell 1982).

For completion, two other “fast tracks” to speciation involving chromosomal organization deserve mention, if only to point out how they sidestep the pachytene checkpoint. As noted in Appendix II, many species have arisen from hybridization between two sexual species. Some of these evade the checkpoint that would doom their descendants by simply avoiding meiosis altogether and reproducing asexually. Other interspecies hybrids overcome hybrid sterility because a mitotic accident has doubled their ploidy (Stebbins 1958). In these, duplication of the chromosomes inherited from both parental species automatically protects the new hybrid species and its offspring from destruction by the pachytene checkpoint; it also strongly isolates the new species from its two parental species, not just by the meiotic checkpoint, but also because crosses between the hybrid and either of the parental species will produce mostly sterile triploids. Very many domesticated plant species arose as interspecies hybrids that diploidized: these include, among others, durum wheat, cotton, potatoes, tobacco, yeast varieties used in baking, and many ornamentals. I note that the above fast track to new species formation is far simpler than auto-polyploidization, in which four-way homolog synapsis and crossing over will cause mis-segregation at anaphase of meiosis I and low fertility. But see Morgan et al. (2021) for how some plants have overcome this obstacle.

### Understanding the interplay between recombination, the pachytene checkpoint, and ultimately speciation, will require an improved understanding of the synaptonemal complex

For simplicity the above section was written as if inversions are the only chromosomal reorganization that inhibits recombination, and that this is due simply to the non-viability of gametes in which crossing over has occurred between an inverted and a non-inverted region of homologous chromatids (as shown in Fig. 10D). Reality is more complicated and less well understood. In inversion heterozygotes, crossing over *is* suppressed within inversions, as expected, but recombination is also highly suppressed just outside inversion breakpoints. In *Drosophila* recombination suppression is absolute for 2 million bp beyond an inversion breakpoint, after which crossover frequencies increase gradually for the next 15–30 million bp (Herickhoff et al. 1993; Navarro and Ruiz 1997). Visualized by light microscopy, even chromosomes containing inversions within inversions appear to synapse surprising well with their non-inverted homologs by contorting themselves into pretzel-like shapes (Gong et al. 2005). Perhaps though, undetected by light microscopy, synapsis fails adjacent to inversion breakpoints as, for example, it is seen to do around translocation breakpoints in tomato meiocytes (Herickhoff et al. 1993). Indeed, perhaps in every type of chromosome structure heterozygote, synapsis, and recombination are faulty near chromosome reorganization break points (Gong et al. 2005; Sherizen et al. 2005; Fuller et al. 2019).

More mysteriously, crossover recombination in one position affects crossover location elsewhere on the same chromosome, and even on other chromosomes in the same cell (Joyce and McKim 2011; Gray and Cohen 2016). This setting of the number and distribution of crossovers is, by some yet-to-be-understood mechanism, due to an interaction between HORMADs and Pch2/PCH2/PCH-2/Trip13, which is the very same interaction that also creates the pachytene checkpoint (e.g., Zanders and Alani 2009; Joyce and McKim 2010; Joyce and McKim 2011; Deshong et al. 2014; Gao and Colaiácovo 2018).

Importantly, it is as yet unclear what underlying structure the pachytene checkpoint is surveilling, but see Rhoades et al., 2021. The lifting of this checkpoint requires synaptonemal complex disassembly, which in organisms as unrelated as flies and budding yeast requires, not just the conserved meiotic AAA^+^ family ATPase (PCH2), but also the histone-deacetylase, Sir2 (San-Segundo and Roeder 1999; Joyce and McKim 2010). Every mutant that disrupts homolog synapsis does not necessarily trigger the pachytene checkpoint, but as few as two inversion breakpoints can do so (Mitra and Roeder 2007; Joyce and McKim 2009, 2010). This seems to imply that the pachytene checkpoint may rely on global homolog synapsis to bring chromosomes together for comparison, but that it reads homolog mismatch locally. The involvement of Sir2 suggests that chromatin structure is somehow involved.

On top of the complex regulation imposed by synaptonemal complex biochemistry, sometimes other complicating cell biology affects which chromosomes can pass into gametes (e.g., the presence of meiotic drive genes, the preferential segregation of the dicentric products of inversions crossovers into polar bodies during oogenesis, and inversions within inversions, which relieve recombination suppression). The pachytene checkpoint may respond to some of the above, and not others. Clearly, deciphering the mechanistic basis for crossover assurance, for crossover interference, for pachytene checkpoint surveillance, and understanding the connection between sexual reproduction and speciation, will ultimately require a *molecular* understanding of meiosis and the synaptonemal complex, as forecast by Lynch et al. (2014) and Lenormand et al. (2016).

### How the pachytene checkpoint helps to drive eukaryotic diversification and sexual differentiation

In making long TUs usable by ensuring they can be faithfully inherited, the pachytene checkpoint may also have accelerated the diversification of the Eukarya. As explained above, once inversion homozygotes appear in a population of inversion heterozygotes, the pachytene checkpoint has the effect of helping secure the reproductive isolation of subpopulations with relative inversions. This initial barrier to gene flow makes additional *adaptive* traits in emerging neo-species heritable. By making hybrid offspring a reproductive dead end, the pachytene checkpoint also facilitates the formation of additional barriers to gene flow, further consolidating the reproductive barrier between what become sister species.

In organisms that must secure mates to produce zygotes, physical, auditory, chemical, and visual cues that focus sexual attention on same-species individuals confer a profound benefit by preventing *gamete* wastage. For a review see Coyne and Orr (1998); and for a case study, Ortiz-Barrientos et al. (2004). Courtship and sexual displays have two opposite and equally important functions. On the one hand, they are a means of seduction, to ignite a mutual attraction between two compatible members of the same species that is sufficiently potent to overcome distance, scarcity of mates, and inhibition so as to set in motion that peculiar and intimate joint act that culminates in gamete fusion. However, courtship must simultaneously repulse, or at least arouse little interest in members of sibling species with whom sex would produce mostly infertile offspring. The tree frog's piercing spring cry, the Luna moth's perfume, the reef squid's dance of lights are not summons to just anyone.

Deterring inter-species romance must be a special challenge in those crowded tropical ecosystems whose species had so engrossed Charles Darwin and Alfred Russel Wallace. In rainforests where intense solar influx and plentiful water make for bountiful habitats and high carrying capacity, sibling species must often live cheek by jowl. The bizarre ballets and ostentatiously beautiful costuming of New Guinea's birds-of-paradise, different in each species, surely arose from this need to catch the eye of none but appropriate partners on the crowded jungle dance floor (https://www.youtube.com/watch?v=rX40mBb8bkU). To avoid squandering precious eggs on an unsuitable mate, female discernment is critical (McPeek and Gavrilets 2006). The lock-and-key combination of hard-to-seduce females needing special male courtships, songs, or visual identifiers to woo them creates a high barrier to cross-species promiscuity.

Within-species mating is rewarded by offspring that have not lost genes as a consequence of error-prone break-repair, that do not carry chromosomal reorganizations which in and of themselves might cause disease, that have a layout of introns and exons (and hence of developmental patterns and eventual phenotypes) that closely matches those of their parents, and that produce a high quotient of viable gametes. In sharp contrast, mating between individuals from different species produces hybrids of low or no fertility as unmatched chromosome arrangements trip the pachytene checkpoint. This stark difference in reproductive success constitutes a powerful motor driving selection for distinctive courtships, exclusive mating-type attractants, and unique lures to tempt species-appropriate pollinators. In this view, the primary utility of visually-striking plumage, for example, is *not* as a surrogate for overall fitness in the competition between same-sex individuals for mates, as is often suggested. Instead, it is a means to signal species identity to potential sexual partners and to discourage imprudent unions that would generate mostly sterile offspring.

I therefore submit that the pachytene checkpoint, which helps guard each species' genetic inheritance against the damage inflicted by unavoidable errors in double-strand break repair, as a side-effect catalyzes the creation of, as Darwin so elegantly wrote, “endless forms most beautiful and most wonderful” (Darwin 1859).

## Conclusions

I propose that the paramount adaptive value of sexual reproduction lies in making it possible for eukaryotes to pass to their offspring, undamaged and unabridged, their ancient treasure troves of fine-tuned, delicate, and intron-laden transcription units with which to construct complex life forms. How fitting then that what so profoundly, urgently, and thrillingly affects our macro world to ensure sexual reproduction—the ibex's horn-clashing fight to secure his mate and the bower bird's artistic labors to seduce one, the perfume and nectar-baited flower to entice pollinators, and the enthralling sweetness and longing of falling in love—should exist to cherish and defend what at the molecular level choreographs bodies and behaviors. Yet, if sexual reproduction is the guardian of genome integrity, how could it be otherwise? We who negotiate the macro world are DNA's avatars. Those long, well-ordered nucleotide sequences that bring each of us into existence must ordain that what is essential for their own continuance is simultaneously of utmost concern and delight to us, lest together we perish from the Earth.

## Supplementary Material

obac008_Supplemental_Files

## Appendix I

### Sexual eukaryotes

Fig. 9 diagrams the most common ways that sexual eukaryotes order mating, meiosis, and the haploid and diploid phases of their lives. The genes needed for synaptonemal complex formation occur throughout the Eukarya, although with differences whose significance for the various eukaryotic lifestyles are as yet not understood (Loidl 2016).

### Haplo-dominant organisms

9A represents the haploid-dominant life cycle typical of many unicellular or morphologically simple multicellular eukaryotes—amoebae, cellular slime molds, lower fungi, unicellular and colonial algae with few cells. In these, only the zygote is diploid and it lives for just one cell cycle. The zygote divides by meiosis and the resulting haploid cells divide mitotically to produce either an exponentially-increasing population of new unicellular organisms, or a multicellular organism composed of haploid cells.

Haploid cells are exposed to direct selection on genetic defects that diploidy would mask. Therefore, *unicellular* haploid-dominant organisms that have lost a TU to faulty break repair, or that have suffered a significantly deleterious mutation, are likely to be eliminated directly by purifying selection. Thus, pachytene checkpoint-induced apoptosis would not be required to filter out TU-destroying mis-repairs whose manifestation is chromosomal reorganization. However, as explained in the main text, mutation, end-joining break repair, and a variety of other repair and replication mistakes can create alleles that are viable, but that have diminished function. These suboptimal alleles tend to be passed on and accumulate as congenital defects. Key to a species being able to eliminate this class of alleles is meiotic recombination. Therefore, for haplo-dominant *unicellular* organisms, the adaptive raison d'etre for mating, meiosis and chromosome synapsis is probably just recombination. Because outcrossing is essential if recombination is to defeat Muller's Ratchet, mating type differences are important even in these simple organisms.

In *multicellular* haploid-dominant organisms, somatic cells with mis-repaired break damage will be prone to the same potential problems that are described in the main text for multicellular diploid organisms—tissue death, tumors, TU destruction etc. If multicellular haploid-dominant organisms make lots of gamete-producing cells, one would expect the pachytene checkpoint to arrest or kill those carrying chromosomal rearrangements, since they flag potential TU destruction. Thus, in multicellular haploid-dominant organisms, the function of mating, meiosis and chromosome synapsis would be expected to include both recombination and the culling of meiocytes that are chromosome rearrangement heterozygotes. (This paragraph is written in the subjunctive because I am not aware of any systematic comparison of synaptonemal complex function in multicellular *vs.* unicellular haploid-dominant eukaryotes.)

### Diplo-dominant multicellular organisms

9D depicts the multicellular diploid-dominant animals. Their life cycles are virtually the inverse of the haploid-dominants: haploid gametes which live not even one full cell cycle and then fuse to produce a diploid zygote from which complex diploid bodies form by successive rounds of mitotic division and cell differentiation. Usually only the gametes are haploid, although in a few species (e.g., pinworms, thrips, bees, wasps, and ants) it is not just the sperm, but also the sperm delivery vehicle—a short-lived male organism—which is haploid. In many animal embryos there is an early separation of somatic and germline precursor cells, with only germline cells retaining meiotic capabilities (as indicated in 9D).

When a lengthy period of diploidy is part of a life cycle, as for the organisms represented by 9B and 9D (and in some cases this period is even prolonged by clonal expansion), DNA breaks occur, are repaired and mis-repaired, and the mis-repairs are passed on by mitosis and therefore accumulate. The TU wreckage caused by the mis-repair of double-strand breaks will be masked by diploidy, which lets complex multicellular organisms live longer than they could if haploid. This advantage may have been what led to the evolution of diploid-dominance in animals (9D) and to the prolongation of the diploid phase that occurred as land plants and marine algae evolved greater complexity (9B; and see below).

Diploidy also masks deleterious alleles, but only when the two homologs carry different alleles. Therefore, different mating types or sexes are advantageous in that they promote outcrossing and population mixing. But diploidy is a bandage and not a fix. Only meiosis is able to bring about the allelic shuffling and genome filtering that creates the opportunity for some offspring to begin life with renewed genomes.

### Haplodiplontic organisms

9B depicts the life cycle that characterizes the land plants. Plants are haplodiplontic, which means they obligatorily alternate multicellular haploid and multicellular diploid phases. The mature diploid entity produces haploid spores by meiosis (green lines with small arrow heads). These haploid cells divide mitotically to produce a multicellular haploid structure, which produces haploid gametes by *mitosis* (note position of GAMETE label in 9B). During plant evolution a gradual shift occurred from the haploid phase being most prominent to the inverse. In the ancient lineages of liverworts, hornworts, and mosses the haploid stage is dominant, with the diploid stage being parasitic on it. In club mosses, ferns, horsetails, gymnosperms, and angiosperms, the diploid stage is dominant with a haploid stage that is small and either free living (club mosses, ferns, horsetails) or parasitic on the diploid stage (gymnosperms and angiosperms). In the flowering plants—angiosperms—the last major plant lineage to appear, meiosis occurs within the flower to produce the haploid spores, which develop into either a male or a female haploid gamete-producing structure by just three mitotic divisions. Fusion of sperm and egg then creates the diploid zygote from which the embryonic portion of the seed develops. Angiosperms require a curious “double fertilization”: the endosperm, that part of the seed that will nourish the growing embryo and the newly germinated plant, is triploid and requires fusion of one haploid male gamete with two haploid sisters of the egg cell nucleus. Although the endosperm does not contribute genetically to the next generation, a seed's requirement for it impedes shifts to asexual reproduction, as explained in Appendix II.

The life-cycle structure of the cellular slime molds and higher fungi is also represented by 9B, although these eukaryotes use a somewhat different way of ensuring that their somatic cells carry duplicate gene copies: when a haploid cell encounters another of its own kind, and of opposite mating type, somatic cell fusion takes place, but without nuclear fusion. The organism then continues growing as a binucleate entity until an appropriate time when the two nuclei in binucleate cells fuse, undergo meiosis and produce haploid spores. The binucleate somatic cells provide the same beneficial masking of deleterious mutants and broken TUs that diploidy provides, extending the lives of individual cells and organisms which might otherwise have succumbed to genetic damage had they remained haploid.

### Diplo-dominant unicellular organisms

9C represents the lives of ciliates and diatoms, rapidly-reproducing and enormously abundant organisms. Unlike most unicellular eukaryotes, they are diploid-dominant. Both diatoms and ciliates practice sex with outcrossing. Diatoms undergo multiple cycles of mitotic division as diploid cells, followed by meiosis, exchange of gametes and fusion to restore diploidy before resuming reproduction by mitosis (Cooper and Masey 2013).

Ciliate reproduction is superficially more complicated because each cell has, in addition to a transcriptionally-inert diploid germline nucleus, a highly polyploid transcriptionally-active somatic nucleus. It was in a ciliate, *Paramecium aurelia*, where it was first shown that DNA damage is cumulative and that after many rounds of mitotic division the members of a clone lose vigor, cease dividing and die, but that mating can restore vigor and the ability to resume mitotic proliferation (Smith-Sonneborn et al. 1974). That the germline nucleus is exempt from the routine, DNA-breaking task of transcription, the polyploidy of the somatic nucleus used for transcription, and that their introns are few and tiny (15 to <100 bp; Bondarenko and Gelfand, 2016; Pan et al. 2019), helps explain why ciliates may undergo up to 200 consecutive mitotic divisions before dying (Smith-Sonneborn et al. 1974). Upon coming together to mate, both conjugal cells undergo meiosis, and then each passes one haploid germline nucleus to its partner; the two haploid nuclei immediately fuse, restoring diploidy. In each newly mated cell, the other three haploid products of meiosis and the old polypoid somatic nucleus degenerate. The rejuvenation brought about by mating and nuclear exchange presumably depends on the new diploid germline nucleus comprising new sets of reshuffled alleles; thus, outcrossing and meiotic recombination are important aspects of sexual reproduction for these organisms. New polyploid somatic nuclei are made by copying the mitotic sisters of the revitalized diploid germline nucleus. Purifying selection during the many subsequent mitotic cycles must be what purges genetic defects from the gene pools of these prolific unicellular organisms. However, given the polyploidy of their somatic nuclei, it must do so with far less efficacy than it does in haploid-dominant unicellular species.

It is noteworthy that ciliates and diatoms (or at least pennate diatoms) are missing some canonical synaptonemal complex proteins, and that electron microscopy reveals either no synaptonemal complex at all, or degenerate lateral elements (Chi et al. 2014; Patil et al. 2015). Gene inventories imply that meiotic recombination *does* occur, initiated by a Spo11 ortholog and carried out by homologous recombination (Chi et al. 2014; Patil et al. 2015). In ciliates, as in other species, inversions will have the power to protect adaptive allele combinations from recombination. But, due to the lack of a pachytene checkpoint, chromosome rearrangement heterozygotes cannot be filtered out. As pointed out in the main text, diatoms exist in innumerable transitional forms, as one might predict for organisms lacking a pachytene checkpoint to cull out viable meiocytes arising from hybridization between lineages with different karyotypes.

### Algae have tried it all

The algae, which are a phenomenally diverse group of eukaryotes of ancient origin, employ almost all of the life cycle options represented in Fig. 9 (Brodie et al. 2017; Umen and Coelho 2019). For example, unicellular and colonial freshwater algae in the Volvox family are haploid-dominant and reproduce as diagrammed in 9A; diatoms, which are unicellular brown algae, are diploid-dominant, as diagrammed in 9C (see above discussion); sea lettuce (Ulva) has separate multicellular haploid and multicellular diploid phases with virtually identical morphologies, while the various kelp species alternate multicellular diploid and multicellular haploid phases, but with the haploid and diploid phases having altogether dissimilar morphologies. Like land plants, the diploid (sporophyte) phase produces haploid spores by meiosis and the haploid (gametophyte) phase at maturity produces the gametes, as diagrammed in 9B.

Many algae further increase their chances of reproductive success by clonal propagation of their diploid somatic tissue: for example, in *Ectocarpus*, the diploid sporophytes produce spores by both meiosis and by mitosis (Coelho et al. 2020). The spores produced by mitosis are clones of their diploid parent, and so merely enlarge the sporophyte population. This is analogous to the many land plants in which suckers, bulbs, rhizomes, etc. expand their diploid somatic lineage, while also producing seeds by sexual reproduction, and to a few animals (e.g., *Hydra*) which reproduce both via somatic buds and sexually. It will be interesting to discover how, during algal evolution, the synaptonemal complex may have changed to incorporate additional functions.

## Appendix II

### Asexual eukaryotes

This appendix provides an overview of the most common modes of asexual reproduction. Its aim is to illustrate some of the ways in which eukaryotes manage without the full repertoire of genome-preserving tools provided by sexual reproduction, and the consequences. (For other surveys of asexuality see Schön et al. 2009; Mirzaghaderi and Hörandl 2016; Galis and Alphen 2020).

### Self-fertilization

“Selfing” is the term used when male and female gametes derived from the same individual fuse. Both self-fertilizing animals and self-pollinating flowers produce their gametes by meiosis (Brandeis 2018). Thus, just as in outcrossing organisms, gamete-producing cells that are chromosome structure heterozygotes can be culled. However, selfing produces fully homozygous offspring, putting the F1 generation at risk for the genetic diseases resulting from deleterious recessive alleles (Charlesworth and Willis 2009). For this reason, it is not surprising that for many organisms, self-fertilization is a fallback strategy, letting these organisms produce possibly inferior offspring in circumstances where they would otherwise produce none. Thus, in some flowering plants that are self-compatible hermaphrodites, stamens or stigma change shape or move as they age, bringing gametes into contact only after the opportunities for cross-pollination have waned (Goodwillie and Weber 2018). Others minimize self-pollination by having male and female gametes mature at different times, with eggs that escape early fertilization by outcrossing remaining receptive to later fertilization by their own pollen (Goodwillie and Weber 2018). An analogous strategy is seen in the self‐fertile but preferentially outcrossing freshwater snail, *Physa acuta.* As compared to individuals with access to mates, solitary snails delay reproduction for about two weeks before resorting to self-fertilization of their own eggs (Tsitrone et al. 2003).

Numerous species that are occasional selfers have given rise to species of obligate selfers—confirmed hermaphrodites that have abandoned mating altogether. Continuous selfing gradually creates allelic homozygosity and eliminates deleterious alleles as the homozygous individuals carrying them die or fail to reproduce. The genome of the predominantly self-fertilizing nematode, *C. elegans*, has been shaped by this process, and thus can produce viable offspring both by self-fertilization and by mating with the rare males that appear in *C. elegans* populations. By contrast, its normally outcrossing relative, *Caenorhabditis remanei*, produces offspring that suffer acutely from diminished viability when inbred (Dolgin et al. 2007). Likewise, the hermaphroditic sea squirt, *Corella inflata*, which normally fertilizes its own eggs within a brood chamber, produces equally viable offspring from selfing and outcrossing. In contrast, *C. willmeriana*, a sibling species that reproduces by broadcast spawning but which otherwise has very similar life-history and traits, shows greatly reduced embryo survival when self-fertilized (Cohen 1996).

Since *obligate* selfing can eliminate both mis-repaired chromosomes carrying wrecked TUs (via the pachytene checkpoint), and deleterious alleles (by repeated recombination and purifying selection), why is obligate selfing not just as successful a long-term strategy as sexual reproduction with outcrossing? As reviewed in the main text, new mutations appear extremely slowly, but they are the raw material for evolutionary adaptation. In a large outcrossing population, many different lineages act as a collection basin for allelic diversity, creating a genetic reservoir, which when conditions change natural selection can draw upon. An obligate hermaphrodite may have a perfect set of alleles for the life it is currently living, and will thrive so long as its environment does not change, but as a species it lacks the allelic heterogeneity needed for further adaptation and to give rise to new species.

### Facultative automixis (parthenogenesis with meiosis): backup option 1

Various animals can reproduce parthenogenetically (without mating) by generating new individuals from unfertilized eggs. In automixis, haploid female pronuclei fuse after completing meiosis and the resultant diploid cell then proceeds to develop. This is a contingency option making reproduction possible when potential mates are scarce due to geography, or when one's life is so extraordinarily short that finding a mate in time might be impossible. Several species of shark and various reptiles, including Komodo dragons and various small lizards and snakes, preferentially reproduce sexually, but in a pinch can produce offspring by automixis (Cole 1975; Watts et al. 2006; Chapman et al. 2007, 2008; Lampert 2008; Booth et al. 2012). Mayflies, which may only live minutes, are invertebrate facultative automicts (Funk et al. 2010; Hiruta et al. 2010). In automixis the two female pronuclei, being the products of meiosis, have passed through the pachytene filter. Thus, automixis should impose no more risk of transmitting rearranged chromosomes with broken TUs than does sexual reproduction. However, at every meiosis, recombination and random segregation of homologous chromosomes will expose new subsets of deleterious mutations to homozygosity, which carries the potential risk of expressing deleterious genes. In Mayflies, for example, offspring lose 10–22% of their variation per parthenogenetic generation (Funk et al. 2010). Since subsequent outcrossing can restore lost allelic diversity, this reproductive strategy is sustainable in the long run.

Flowering plants appear unable to produce seeds by simple automixis; this is likely because the endosperm (the tissue that nourishes the embryo inside the seed) is triploid and requires the fertilization of two female pronuclei by one haploid male gamete (see Appendix I).

### Facultative apomixis (parthenogenesis without meiosis): backup option 2

Some short-lived invertebrates reproduce both sexually, and parthenogenetically *without* meiosis. During apomictic reproduction, diploid primary oocytes develop directly into zygotes and thence into new individuals. There is no meiosis, no homolog synapsis, no recombination, no pachytene checkpoint, no reduction divisions, and therefore no necessary fusion with another gamete. Offspring produced by apomixis are full genetic clones of their mother. Among animals, apomicts are usually seasonally or cyclically asexual. Cyclical apomixis is not meiosis abandoned, but meiosis temporarily skipped (often during circumstances that permit explosive population increase). Aphids, for example, reproduce clonally throughout the summer when food is plentiful, but resort to sex to produce their overwintering eggs (Simon et al. 2002).

Without the pachytene checkpoint, apomicts cannot avoid creating a larger fraction of oocytes with mis-repaired breaks than if their eggs were produced meiotically. But when food is in unlimited supply, the *absolute number* of viable offspring produced without the delays and complications of mating and meiosis may well exceed the number that could be produced by sexual reproduction. Similarly, when mates are nowhere to be found, a small number of viable children is better than no children at all. Thus, facultative apomixis should be understood as a reproductive strategy that may succeed, even though it risks producing a significant number of progeny that are unhealthy.

Indeed, offspring produced by facultative parthenogenesis, whether by automixis or apomixis, fare notably less well than their sexually-produced kin (Lamb and Willey 1979; Carballa and Rivera 2007). Whereas inbreeding depression in facultative *automicts* results from unmasking homozygous recessive deleterious mutations, in facultative *apomicts* those exceeding damaging DNA break repair mistakes that would normally be filtered out by the pachytene checkpoint are now obligatorily passed on too. The cockroach, *Nauphoeta cinerea*, illustrates the dramatic difference that sexual *vs*. asexual reproduction can make for a facultative apomict (Corley and Moore 1999): over twice as many mated as unmated females gave birth, with mated females producing more than twice as many broods of eight times the size. Moreover, second generation parthenogenetic offspring are vanishingly rare and third generation offspring non-existent. Perhaps, further study will reveal what makes the *Nauphoeta* genome so prone to end-joining repair mistakes.

### Obligatory apomixis can lead to evolutionarily short lives

*Obligate* apomictic invertebrates commonly arise from hybridizations between species that are able to reproduce both sexually and asexually, often as facultative apomicts (Otto and Whitton 2000; Neaves and Baumann 2011; Lenormand et al. 2016). For example, although most lineages of the water flea, *Daphnia pulex*, are cyclically parthenogenetic, numerous obligatorily parthenogenetic lineages have arisen by hybridization with *D. pulicaria*. Sequence analysis of 11 cyclically parthenogenetic isolates and 11 obligate asexual isolates suggest that the average age of the extant asexual lineages is only about 22 years (Tucker et al. 2013). It is important to note that, despite their short existence, the genomes of the completely asexual *Daphnia* have already accumulated high levels of chromosomal rearrangements and deletions. This is what would be expected if hybridization is producing mis-synapsis, and mistakes that would normally have been screened out of the gene pool by the pachytene checkpoint are instead being passed on. Apomixis in these hybrid water fleas may have been selected for as a way to evade the pachytene checkpoint, but this very evasion may also seal their fates as short-lived species.

### Some obligate apomicts subsist in marginal habitats

The *obligate apomictic plants* are virtually all polyploids of hybrid origin, which curiously inhabit marginal ecosystems, such as deserts and glaciated terrain, where their sexual relatives do not live (Bell 1982; Asker and Jerling 1992; Kearney 2005; Hörandl 2009). This odd combination of traits may be due to the interaction of their genetic inheritance with the pachytene checkpoint. Their mixed parentage has presumably equipped these hybrids to colonize habitats that neither of the parental genomes by itself had the genes to exploit (Kearney 2005). However, as explained in the main text, species differ by chromosomal organization. Therefore, interspecies hybrids have trouble aligning their chromosomes during synaptonemal complex formation. In response to this, the pachytene checkpoint will turn synapsis failure into a failure to produce viable gametes; this gives a great selective advantage to mutants that evade this perilous checkpoint by avoiding meiosis altogether and reproduce asexually. Although natural selection may have produced obligatory apomixis as an immediate solution to interspecies hybridization, that very solution may be what condemns these hybrid plant species to a short and brutish existence. Without the synaptonemal complex, neither recombination nor the pachytene checkpoint exist, leaving these asexual lineages unable to escape Muller's Ratchet and unable to filter out genomes that have lost TUs to break mis-repair. Being polyploids, they presumably carry at least twice as many copies of most genes as either of their parental species, and this polyploidy should delay when in the life of each species their genetic problems become manifest. Yet, in a head-to-head competition, in an environment for which the sexual and asexual plants are equally well adapted, the sexual species, being better able to avoid passing on newly acquired genetic defects, would presumably outlast its asexual competitor. This might explain why obligate apomictic plants are found in barren habitats where they manage to survive, but where they have not had to compete with their sexual cousins.

Apomictic flowering plants must surmount a further problem: whereas an unreduced and unfertilized gametophyte cell can give rise to the seed's zygote by mitotic division, the endoderm normally requires fertilization by a haploid male gamete to create its normal triploid genotype (with 2 maternal +1 paternal chromosome sets). Artificially selecting for hybrid apomicts produces a very high percentage of non-developing seed due to failure of the endosperm to form (Barke et al. 2018). Some naturally-occurring apomictic plants are small-seeded species where the embryo can survive without endosperm; others survive because they are fertilized by non-hybrid *pollen* from one of the two parental species, which allows the endosperm to form with the correct contribution of one paternal genome plus the diploid maternal contribution; yet others have managed to evolve complex genetic work-arounds (Hojsgaard and Hörandl 2019).

### Some obligate apomicts are saved by high levels of ploidy

At least 90 species of salamanders, frogs, and fresh water fish are obligate polyploid apomicts (i.e., they reproduce without meiosis). These lineages of vertebrate animals, like most obligate apomicts, originated by interspecies hybridizations. The Ambystomatid salamanders are the oldest lineage of vertebrate apomicts. They survive as ploidy-variable females (triploid through pentaploid), the descendants of hybridizations amongst at least four species (Hedges et al. 1992). As noted in Appendix I, increasing ploidy will mask assorted allelic defects produced by mutation, replication errors etc., as well as TU loss due to break mis-repair. If, during one lifetime, 1/100 genes in a genome are normally ruined by chromosomal reorganization or mutation, for a ploidy level of 2N, 3N, 4N, or 5N the odds that *the same gene* in any cell will have been destroyed drops to 1/100^2^; 1/100^3^; 1/100^4^ ; and 1/100^5^. Thus, even without the meiotic pachytene filter there is a good chance that some eggs in every clutch will retain intact copies of all their TUs. It is then purifying selection, rather than the pachytene checkpoint, that filters the genome in each generation. An all-female lineage of polyploid Ambystomatid salamanders appears to have perpetuated itself in this way for between 2 and 4 million years, via purely mitotic divisions.

### The evolutionary longevity of the Bdelloid rotifers: infrequent or unconventional sex?

The most ancient of the apparently obligate apomictic invertebrates—the bdelloid rotifers—may be unique amongst eukaryotes in having found an actual *replacement* for sex. Extant bdelloids reproduce as parthenogenetic females, producing diploid eggs by mitosis, with no cytological evidence of meiosis, or chromosome synapsis, or any confirmed sightings of males. Yet, bdelloids have avoided the early extinction typical of obligate apomicts, with females being found in 30–40 million year old amber and genetic evidence implying that they may be twice that age (Mark Welch and Meselson 2000). Moreover, they have diversified into 4 families, 19 genera, and 400–500 morphologically-distinct species (Mark Welch et al. 2009).

Bdelloid rotifers are ubiquitous invertebrates, living in fresh water habitats, including in some, such as puddles and leaf litter, that are ephemeral. Intermittent desiccation inflicts acute DNA breakage, which bdelloids have evolved the ability to survive. Strikingly, both somatic and germline cells (even oocytes in G1 of the cell cycle) are able to withstand levels of ionizing radiation that produces hundreds of double-strand breaks per cell, damage levels well beyond what kills other eukaryotes (Gladyshev and Meselson 2008; Gladyshev and Arkhipova 2010). The monogonont rotifers, a sister taxon to the bdelloid rotifers, are facultatively asexual and lack the bdelloids’ resistance to both desiccation and high levels of ionizing radiation.

In view of the argument that meiosis and sex is what allows most eukaryotic species to contend with Muller's Ratchet and the inevitable errors resulting from mis-repair of double-strand breaks, how have bdelloids persisted, and even diversified, apparently without males, mating, selfing, homolog synapsis, or meiosis (Mark Welch and Meselson 2000)? Perhaps bdelloid males do exist and mating does occur, but surreptitiously and only rarely (Laine et al. 2021). Alternatively, there is reason to suspect that bdelloids may be resorting to something analogous to DNA transformation, that ancient rescue mechanism used by Eubacteria and Archaea where DNA is exchanged directly (Eyres et al. 2015). Might this alternative way of obtaining DNA to mask damage be what enables bdelloids to abandon sexual reproduction without the usual long-term bad consequences?

The bdelloid species sequenced to date are all degenerate tetraploids, due to an ancient hybridization event (Mark Welch et al. 2008; Hur et al. 2009; Flot et al. 2013; Nowell et al. 2018). Perhaps it was that hybridization between two species—that by making avoidance of the pachytene checkpoint necessary—set these organisms on the path to compulsory asexuality. Tetraploidy would have temporarily provided supplemental sets of genes with which to mask damaged ones. However, all bdelloid genomes are unusual in that their chromosomes include *thousands* of genes acquired by horizontal gene transfer—from bacteria especially, but also from an enormous assortment of eukaryotic species (Gladyshev et al. 2008; Flot et al. 2013; Eyres et al. 2015; Nowell et al. 2018). Many of the genes that have been nabbed from other organisms encode full-length proteins (mostly enzymes), which the bdelloids are transcribing and translating (Mark Welch et al. 2008).

Among the various bdelloid species, some have taken up lives in perpetually aquatic habitats. Those species that have not done this, and which must continue to contend with repeated cycles of desiccation, and therefore higher levels of DNA breakage, have *smaller* genomes, but which amazingly contain about twice as many genes as those bdelloid species that have escaped routine desiccation (60,000 to 65,000 genes in 200 million bp genomes, *vs.* 25,000 to 35,000 genes in 400–500 million bp genomes; Nowell et al. 2018). That is, in bdelloid species that routinely suffer desiccation, natural selection has selected for shorter TUs, making each TU less susceptible to double-strand breaks. But, in addition, it has selected for genomes consisting of about double the usual number of genes. It may be that having a backup of redundant genetic information is key to these bdelloids being able to survive onslaughts of DNA breakage, irrespective of cell cycle phase.

While half of their foreign genes were clearly acquired millions of years ago, prior to the divergence into the current-day bdelloid species, it is also clear that acquisition of new foreign genes is ongoing, that it is highest in those species that are resistant to desiccation, and that morphologically-distinct species are differentiated by hundreds of acquired foreign genes (Eyres et al. 2015). Although the rate of acquisition and domestication of foreign genes (not more than 13 new genes per million years) is not believed to be fast enough to substitute for sex (Eyres et al. 2015), this acquisition does reveal that these tough little Argonauts scavenge genes from the genetic flotsam and jetsam of dead organisms that turn up in their watery surrounds. Bdelloid ovaries are immediately adjacent to and envelop their stomachs. Dead organisms sucked into their digestive tracks are the likely source of the DNA from which homologous recombination and/or non-homologous end-joining recruit compatible sequences into germline chromosomes. That the foreign genes which bdelloids have incorporated are from organisms that are their common food sources supports the plausibility of this idea.

Whole genome comparisons of 11 wild-caught individuals of the best-studied bdelloid species (*Adineta vaga)* show allelic diversity patterns that are *incompatible* with clonal inheritance alone (Vakhrusheva et al. 2020): homologous alleles are present in close to Hardy-Weinberg ratios and different genes are assorting at random. This is strong evidence that somehow genetic exchange between individual members of this species is occurring at levels comparable to what sex and recombination usually accomplish. Furthermore, studies of another bdelloid species (*Macrotrachella quadricornifera)* revealed that the lengths of exchanged DNA can be large (up to 150,000 bp; Laine et al. 2021). Meselson and colleagues therefore conclude that mating must be occurring, and that the lack of observing it is merely due to its infrequency and to searching for males in all the wrong places (Laine et al. 2021). Yet, to this author, transfer of DNA by means other than sexual intercourse does *not* seem to be ruled out (Eyres et al. 2015). Whatever mechanism lets bdelloids incorporate DNA from foreign species, should also let them incorporate DNA from other bdelloids, and this may be how they obtain supplemental genes to enlarge their genomes. Many of the indispensable benefits that sexual eukaryotes obtain by sex with outcrossing, bdelloids may be enjoying by what is, in effect, necrophilia.
