# Supplementary material for: Does the Pachytene Checkpoint, a Feature of Meiosis, Filter Out Mistakes in Double-Strand DNA Break Repair and as a side-Effect Strongly Promote Adaptive Speciation?
Source: Integr Org Biol. 2022 Apr 8;4(1):obac008. doi: 10.1093/iob/obac008 (PMC8998493; doi:10.1093/iob/obac008)
Supplement: obac008_Supplemental_Files [file obac008_supplemental_files.zip › Figure_S1_legend.docx]

**Supplemental Figure S1. Interphase chromatin before DNA replication.** “Miller spreads” are made by lysing nuclei and dispersing their chromatin in distilled water adjusted to pH 8.5, followed by low-speed centrifugation of chromatin onto a TEM grid. This produces a 2-D projection of a greatly expanded interphase nucleus. In regions of light dispersal, as shown here, the beads-on-a-string strands of nucleosomal chromatin—the so-called 10 nm fiber—trace out crenate patterns with randomly-folded side-branches of no particular repeat length (see also non-transcribed chromatin in Figs 2A and 2B.) Strands lie as one might expect of a slightly stiff crumpled-up ball chain, with what may be occasional points of inter-strand affinity. In chromatin spreads from interphase nuclei, I do not see the thicker twisted strands observed in mitotic chromosomes (Fig 6), even in regions of poor dispersal, nor any discernible repeat pattern as might be expected from the dissolution of a higher order helical structure. The disposition of interphase chromatin in Miller spreads appears consistent with random folding of the 10 nm fiber (Nishino et al., 2012), and the looped domains in Figs 5, 7 and 8 are drawn to imply this. Bar shows 2 microns.
