# Supplementary material for: Does the Pachytene Checkpoint, a Feature of Meiosis, Filter Out Mistakes in Double-Strand DNA Break Repair and as a side-Effect Strongly Promote Adaptive Speciation?
Source: Integr Org Biol. 2022 Apr 8;4(1):obac008. doi: 10.1093/iob/obac008 (PMC8998493; doi:10.1093/iob/obac008)
Supplement: obac008_Supplemental_Files [file obac008_supplemental_files.zip › Figure_S2_legend.docx]

**Supplemental Figure S2**. **Transcription units on paired homologs.** After S-phase, it is normal to observe two identical sister chromatid TUs laying side-by-side or in close proximity (e.g., Fig 2). It is exceedingly rare to find four identical TUs side-by-side, as shown here. These four are presumably replicated homologs that managed to find each other in the nucleus, perhaps as a consequence of break repair and direct annealing prior to replication. Almost identical lengths (about 1.09 microns) and tiny intron lariats with attached spliceosomes on the 5’ ends of the transcripts establish these four as identical TUs. This chromatin preparation was from a *Drosophila* embryo 100 min after cycle 14 began (21° C).
