# Supplementary figures and images for: Does the Pachytene Checkpoint, a Feature of Meiosis, Filter Out Mistakes in Double-Strand DNA Break Repair and as a side-Effect Strongly Promote Adaptive Speciation?
Source: Integr Org Biol. 2022 Apr 8;4(1):obac008. doi: 10.1093/iob/obac008 (PMC8998493; doi:10.1093/iob/obac008)

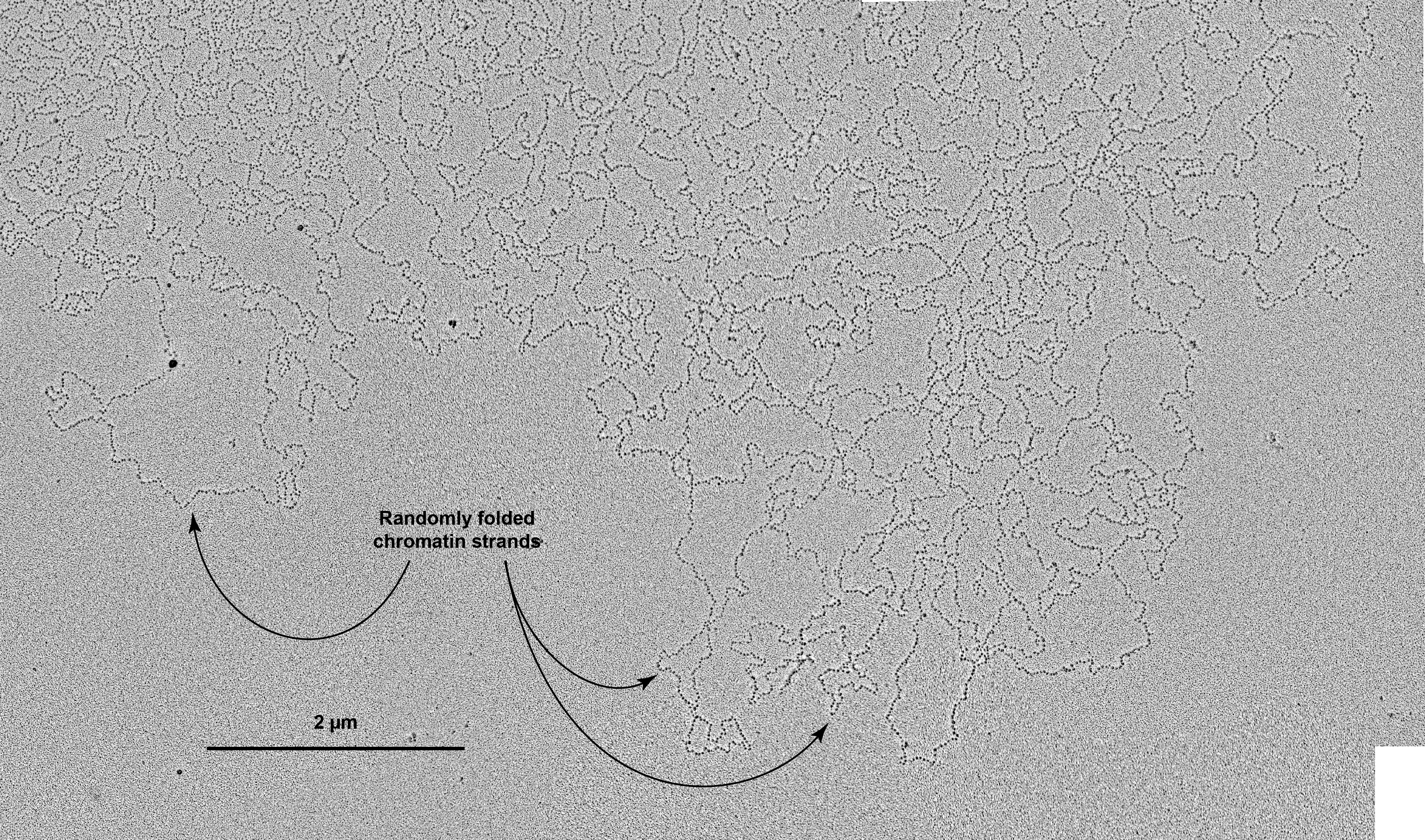

Supplement: obac008_Supplemental_Files [file obac008_supplemental_files.zip › Fig_S1_final_300.tif]

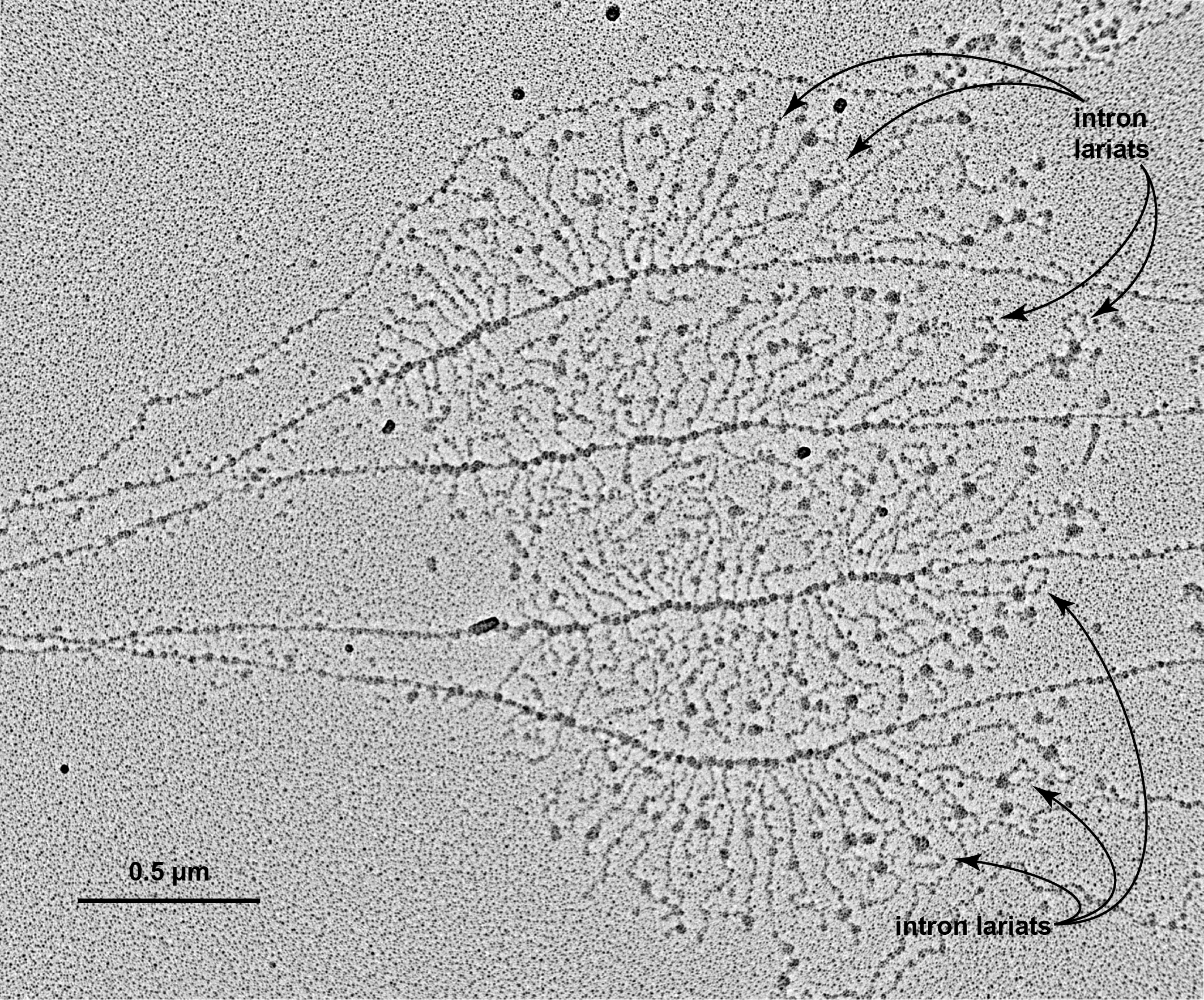

Supplement: obac008_Supplemental_Files [file obac008_supplemental_files.zip › Fig_S2_final_300.tif]
